# Comprehensive Gene Expression Profiling Reveals Synergistic Functional Networks in Cerebral Vessels after Hypertension or Hypercholesterolemia

**DOI:** 10.1371/journal.pone.0068335

**Published:** 2013-07-16

**Authors:** Wei-Yi Ong, Mary Pei-Ern Ng, Sau-Yeen Loke, Shalai Jin, Ya-Jun Wu, Kazuhiro Tanaka, Peter Tsun-Hon Wong

**Affiliations:** 1 Department of Anatomy, National University of Singapore, Singapore; 2 Department of Physiology, National University of Singapore, Singapore; 3 Department of Pharmacology, National University of Singapore; 4 Neurobiology and Ageing Research Program, Life Sciences Institute, National University of Singapore, Singapore; Massachusetts General Hospital/Harvard Medical School, United States of America

## Abstract

Atherosclerotic stenosis of cerebral arteries or intracranial large artery disease (ICLAD) is a major cause of stroke especially in Asians, Hispanics and Africans, but relatively little is known about gene expression changes in vessels at risk. This study compares comprehensive gene expression profiles in the middle cerebral artery (MCA) of New Zealand White rabbits exposed to two stroke risk factors i.e. hypertension and/or hypercholesterolemia, by the 2-Kidney-1-Clip method, or dietary supplementation with cholesterol. Microarray and Ingenuity Pathway Analyses of the MCA of the hypertensive rabbits showed up-regulated genes in networks containing the node molecules: UBC (ubiquitin), P38 MAPK, ERK, NFkB, SERPINB2, MMP1 and APP (amyloid precursor protein); and down-regulated genes related to MAPK, ERK 1/2, Akt, 26 s proteasome, histone H3 and UBC. The MCA of hypercholesterolemic rabbits showed differentially expressed genes that are surprisingly, linked to almost the same node molecules as the hypertensive rabbits, despite a relatively low percentage of ‘common genes’ (21 and 7%) between the two conditions. Up-regulated common genes were related to: UBC, SERPINB2, TNF, HNF4A (hepatocyte nuclear factor 4A) and APP, and down-regulated genes, related to UBC. Increased HNF4A message and protein were verified in the aorta. Together, these findings reveal similar nodal molecules and gene pathways in cerebral vessels affected by hypertension or hypercholesterolemia, which could be a basis for synergistic action of risk factors in the pathogenesis of ICLAD.

## Introduction

Atherosclerotic stenosis of large arteries at the base of the brain or intracranial large artery disease (ICLAD) is a major cause of stroke especially in Asians, Hispanics and Africans [1], and is possibly the most common vascular lesion in the world [2]. It affects the middle cerebral artery (MCA), intracranial portion of the internal carotid artery, vertebrobasilar artery and the posterior and anterior cerebral arteries [1]. ICLAD carries a poor prognosis in terms of subsequent vascular event and death, and there is 25 - 30% incidence of recurrence in the 2 years after stroke [3], [4]. The disease is also prevalent among 53% of vascular dementia and 18% of Alzheimer’s disease patients of Asian ethnicity [1], [5].

The risk factors for ICLAD include hypertension, diabetes, hypercholesterolemia and cigarette smoking [6], and a strong association is found between asymptomatic ICLAD presenting as intracranial stenosis or calcification with large artery stiffness, and patients with untreated hypertension [7]. Arterial stiffness is a major determinant of increased systolic blood pressure, and is associated with lesions in intracranial arteries [8]. Prolonged elevation of blood pressure leads to reduction in vessel cross sectional area, increased wall thickness and accelerated plaque formation [9], [10]. Moreover, hypertension is thought to drive the atherosclerotic changes from larger to smaller vessels, and from extracranial- to intracranial vessels [11], [12]. Hypercholesterolemia is also a risk factor for ICLAD [6], and ischemic stroke from both extracranial and intracranial large-artery atherothromboembolism is associated with increased dietary intake of saturated fat, physical inactivity, obesity, and diabetes [13]. Reduction of cholesterol levels with statin treatment delays the progression of lesions in patients with ICLAD [14]. Increased lipoprotein is an independent biochemical risk factor for the development of ICLAD [15], and increased serum cholesterol is associated with elevated levels of oxidized low density lipoprotein [16]. The latter inhibits nitric oxide in endothelial cells to induce vasospasm [17] or increases tissue factor activity in these cells, to promote thrombosis [18]. Other factors that could contribute to ICLAD include increased oxidative stress in vessel walls [19]. A combination of hypercholesterolemia and hypertension may result in greater damage to vessels [9], [20].

Epidemiological studies indicate that there is increased risk of a second stroke especially in the first 1 or 2 years of post-stroke event [3], [4], [21], [22]. The reasons for this are not fully understood, but almost certainly involve gene expression changes at the vascular level that drive the atherothrombotic process. Thus far, however, there have been no studies to delineate global gene expression or gene network profiles in large intracerebral arteries at risk of atherothrombosis.

The present study was carried out to compare gene expression and morphological changes in intracranial vessels of rabbits, after exposure to hypertension and/or hypercholesterolemia. These conditions were induced by mostly non-genetically based methods, to reduce possible confounding effects during microarray analysis. The middle cerebral artery (MCA) was chosen for study, as this vessel is often affected in ICLAD [1], [23], [24], [25].

## Materials and Methods

### Animals

Male New Zealand White rabbits were used as it is the gold standard in atherosclerosis studies [26]. Although it is possible to produce hypertension in rats and mice, it is difficult to produce hypercholesterolemia in these animals [27]. The very small size of the MCA in rats and mice also hinders gene expression analyses of these vessels. Rabbits were approximately 8 weeks old (young adults) and weighed 2.0–2.5 kg each at the start of the experiments. Two sets of experiments were carried out: i) to determine gene expression changes in the MCA after hypertension, and ii) to determine gene expression changes in the MCA after hypercholesterolemia plus sham operation, and gene expression changes in the MCA after hypertension plus hypercholesterolemia. The first set of experiments were carried on 6 rabbits with the Goldblatt 2-Kidney 1-Clip (2K1C) method used to induce hypertension and fed with normal diet, vs. 6 sham operated controls on a normal diet. The second set of experiments were carried out on 6 rabbits on a high cholesterol diet with sham operation, 6 rabbits with 2K1C to induce hypertension plus a high cholesterol diet, and 6 rabbits on a normal diet.

The 2K1C procedure to induce hypertension was carried out as previous described [28]. In brief, animals were anesthetized with ketamine (75 mg/kg)/xylazine (10 mg/kg) cocktail followed by isoflurane maintenance, and the left renal artery exposed. The artery was partially occluded by attachment of a U-shaped silver ‘clip’ with a 0.6 mm slot. The clip was in left in place until the animals were sacrificed. Sham operated animals received the same surgical procedures as the 2K1C group, except that the renal artery was not partially occluded after its exposure. Animals that were subsequently treated with high cholesterol diet were allowed to recover from surgery for 1 week before treatment with diet containing cholesterol. Rabbits on this diet were fed with GPR diet +1% cholesterol (Glen Forrest Stockfeeders, Australia). Sham operated control rabbits were fed with GPR diet without cholesterol. All procedures including animals were approved by the Institutional Animal Care and Use Committee of the National University of Singapore, and carried out in accordance with guidelines of the National Advisory Committee for Laboratory Animal Research.

### Measurement of Body Weight, Mean Arterial Pressure and Serum Total Cholesterol

Rabbits were anaesthetized by intramuscular injection of ketamine/xylazine cocktail, followed by mean arterial pressure measurements, and collection of blood. Mean arterial pressure was recorded from the ‘middle’ ear artery (Powerlab 4/30, AD Instruments, CO, USA), and blood samples obtained for cholesterol analysis, at 0, 4, 10 and 12 weeks. Approximately 3 mL of blood was withdrawn from the artery and collected in BD Vacutainer® Serum Tubes with Clot activator and silicone-coated interior (Becton Dickinson, NJ, USA). Whole blood was centrifuged at 1,000 g for 15 min, and the serum transferred to new vials and kept frozen at −80°C till analysis. Serum total cholesterol levels were measured by a fluorometric assay (Ex/Em 535/587 nm, BioVision Inc., CA, USA). Samples were analyzed in triplicates and read with a microplate reader (Infinite® i-control, Tecan Trading AG, Switzerland).

### Tissue Harvesting and RNA Extraction

The 2K1C rabbits on normal diet, sham operated control rabbits on normal diet, or hypercholesterolemia plus sham operated rabbits and hypertension plus hypercholesterolemia rabbits were sacrificed 12 weeks after surgery. Sham operated control rabbits or untreated controls on a normal diet were sacrificed after a similar time. Animals were deeply anaesthetized by ketamine/xylazine cocktail and euthanized by intravenous injection of pentobarbital (250 mg/kg). The brains were removed and hemisected. The left half of the brain was immersed in 4% paraformaldehyde in 0.1M phosphate buffer in preparation for histology or electron microscopy (see below). The right MCA was identified and quickly stripped off the surface of the brain without any underlying cortical tissue, immersed in RNAlater® (Ambion, TX, USA), frozen in liquid nitrogen and stored in −80°C till further analysis. Total RNA was extracted using TRizol reagent (Invitrogen, CA, USA) according to the manufacturer’s protocol. Extracted RNA was purified using the RNeasy® Micro Kit (Qiagen, CA, USA). The cerebral neocortex, hippocampus, liver, kidney, aorta and other organs were also removed and snap frozen or stored in paraformaldehyde for future analyses.

### DNA Microarray Analysis

Ten µL of total RNA from the MCA of four rabbits from each group were submitted to Genomax Technologies, Singapore. RNA quality was confirmed using an Agilent 2100 Bioanalyzer. cRNA was then generated and labeled using the one-cycle target labeling method, and hybridized to the 1-colour Agilent Rabbit Microarray (G2519F-020908; Agilent Technologies, CA, USA), according to the manufacturer’s protocol. Data was collected and exported into GeneSpring v11 software (Agilent Technologies) for analysis, using a parametric test based on the cross gene error model. Differentially expressed genes (DEGs) are those that show significantly increased or decreased expression compared to sham-operated controls using one-way ANOVA with Tukey HSD post-hoc test and corrected for multiple comparisons using Benjamini Hochberg FDR. *P*<0.01 was considered significant. In this study, to reduce false positives, only DEGs with greater than 4-fold change (or in the case of common genes between two data sets, greater than 4-fold change in at least one data set) were presented and used in IPA network analyses.

### Network Analyses

The gene sets were analyzed using the Ingenuity Pathway Analysis (IPA) software (Ingenuity® Systems, www.ingenuity.com). Gene identifiers and corresponding expression values of up-regulated or down-regulated DEGS with more than 4-fold change was uploaded into IPA application. Each identifier mapped to its corresponding object in Ingenuity’s Knowledge Base, and was overlaid onto a global molecular network developed from information contained in the Ingenuity Knowledge Base. “Focus Genes” (Network Eligible genes) are defined as DEGs that have at least one other molecule in the Knowledge Base that interacts with it to form a “network”. The latter shows interactions between focus genes and ‘node molecules’ in the network, and how they work together at the molecular level.

### Electron Microscopy

The left half of the brain was dissected out, fixed in 4% paraformaldehyde and 0.1 M phosphate buffer, and kept at 4°C. Blocks containing the MCA were osmicated, dehydrated in an ascending series of ethanol and acetone and embedded in Araldite. Thin sections were cut, mounted on Formvar coated copper grids and stained with lead citrate. They were viewed using a Jeol 1010 electron microscope (Jeol, Tokyo, Japan).

### Quantitative Real-time PCR

The mRNA of one of the node molecules identified by IPA, HNF4A, was further verified in the aorta by real-time RT-PCR. This was necessary, as only a small amount of RNA could be extracted from the rabbit MCA. Purified RNA from the descending aorta of 4 hypercholesterolemia plus sham, 4 hypertension plus hypercholesterolemia and 4 untreated control rabbits were reverse-transcribed with the High-Capacity cDNA Reverse Transcription Kit (Applied Biosystems, CA, USA). Reaction conditions were 25°C for 10 min, 37°C for 120 min and 85°C for 5 sec. Real-time PCR amplification was performed using the 7500 Real time PCR system with TaqMan® Universal PCR Master Mix and probes. The PCR conditions were initial incubation of 50°C for 2 min and 95°C for 10 min, followed by 40 cycles of 95°C for 15 sec and 60°C for 1 min. Rabbit TaqMan® probe for HNF4A was purchased from Applied Biosystems. Rabbit ß-actin TaqMan® probe was used as an internal control. The fold change in expression was calculated using the 2-delta delta CT method as described previously [29]. Possible significant differences between the means were analyzed, using one-way ANOVA with Bonferroni’s multiple comparison post hoc test. *p*<0.05 was considered significant.

### Western Blot Analysis

Aorta samples were homogenized in 10 volumes of ice-cold lysis buffer (150 mM sodium chloride, 50 mM Tris–hydrochloride, 0.25 mM EDTA, 1% Triton X-100, 0.1% sodium orthovanadate, and 0.1% protease inhibitor cocktail, pH 7.4), followed by centrifugation at 10,000 g for 10 min at 4°C. The supernatant was then collected, and protein concentrations measured using the Bio-Rad protein assay kit. The homogenates (20 µg) were resolved in 10% SDS-polyacrylamide gels under reducing conditions and electrotransferred to a polyvinylidene difluoride (PVDF) membrane. Non-specific binding sites on the PVDF membrane were blocked by incubation in 5% non-fat milk in tris-buffered saline-0.1% Tween 20 (TBST) for 1 h. The PVDF membrane was incubated overnight at 4°C with a mouse monoclonal anti-HNF4A antibody (K9218, Abcam, Cambridge, UK) diluted 1∶500 in 5% non-fat milk/TBST. After washing with TBST, the membrane was incubated with horseradish peroxidase-conjugated secondary anti-mouse IgG (Pierce, IL, USA) for 1 h at room temperature. Immunoreactivity was visualized using a chemiluminescence substrate (Millipore, MA, USA). Loading controls were carried out by incubating the blots at room temperature for 30 min with stripping buffer (100 mM 2-mercaptoethanol, 2% SDS, and 62.5 mM Tris-hydrochloride, pH 6.7), followed by reprobing with a mouse monoclonal antibody to ß-actin (Sigma, MO, USA; diluted 1∶10,000 in TBST). Exposed films containing blots were scanned and densities of the bands normalized to those of ß-actin. Possible significant differences between the values from treated and control rabbits were analyzed, using one-way ANOVA with Bonferroni’s multiple comparison post hoc test. *p*<0.05 was considered significant.

### Histochemistry and Immunohistochemistry

Aorta samples were sectioned at 40 µm using a freezing microtome. Sections were processed for histochemistry using Masson’s Trichrome histochemical stain, or immunoperoxidase staining. The latter sections were incubated in a blocking solution composed of 5% donkey serum (Vector) and 0.1% Triton X-100 for 1 h, followed by incubation with mouse monoclonal antibody to HNF4A (diluted 1∶100 in PBS) overnight. The sections were then washed three times in PBS and incubated with biotinylated anti-mouse secondary antibody. Immunoreaction product was visualized using an avidin-biotinylated horseradish peroxidase kit (Vector Laboratories, Burlingame, USA). Histochemically or immunohistochemically stained sections were mounted on glass slides and viewed using a light microscope (IX70, Olympus, Japan).

## Results

### 1. Body Weight, Mean Arterial Pressure and Serum Total Cholesterol Levels

**Figure 1:**
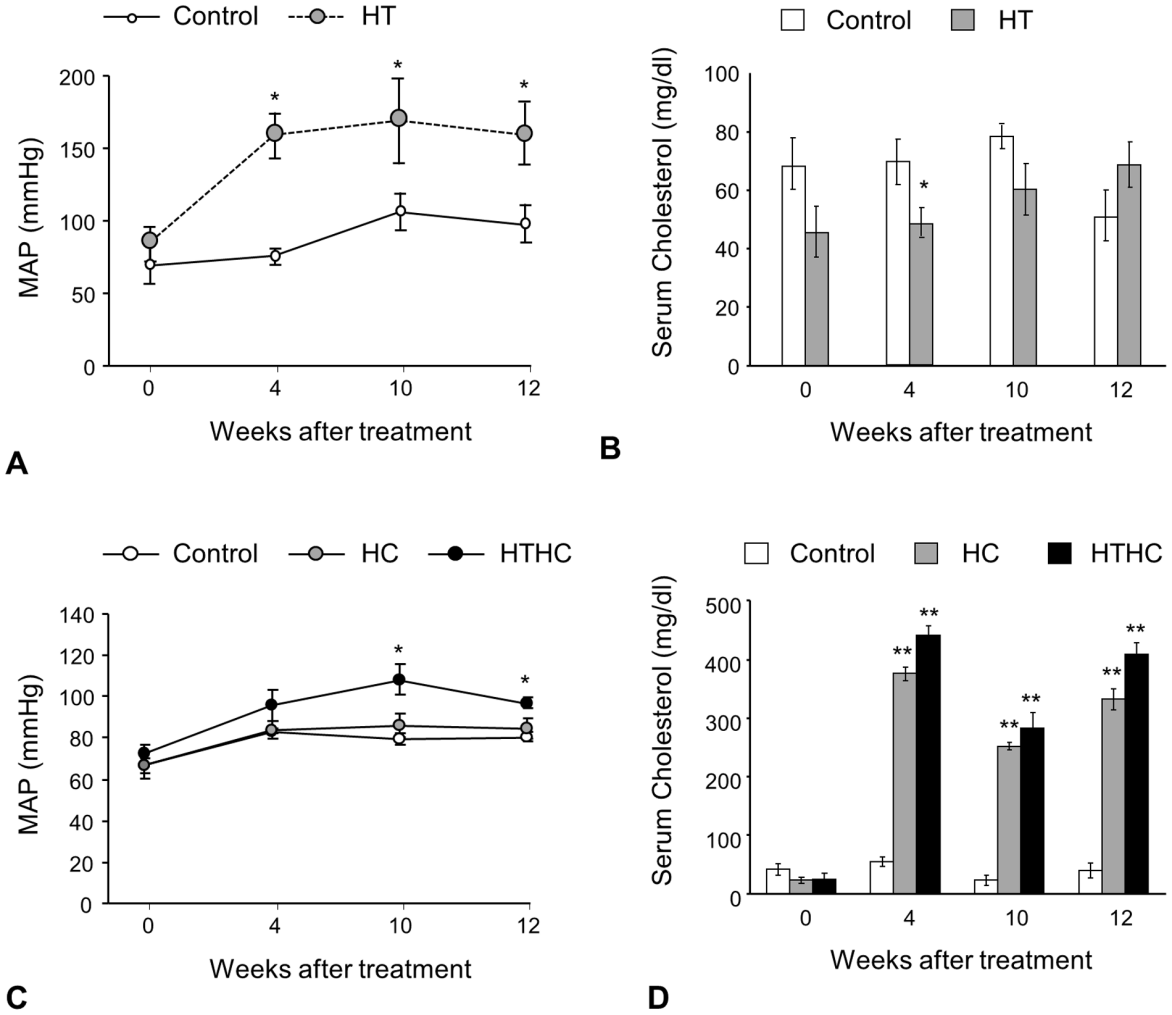
Mean arterial pressure and serum cholesterol levels in rabbits. (A) Mean arterial pressure in hypertension only rabbits. (B) Serum cholesterol levels in hypertension only rabbits. (C) Mean arterial pressure in hypercholesterolemia plus sham- and hypertension plus hypercholesterolemia rabbits. (D) Serum cholesterol levels in hypercholesterolemia plus sham- and hypertension plus hypercholesterolemia rabbits. H: Hypertension only. HC: Hypercholesterolesterolemia plus sham operation. HTHC: Hypertension plus hypercholesterolemia. MAP: mean arterial pressure. Data are expressed as mean ± SEM. *p<0.05, **p<0.01 vs. control (Student’s t-test in A,B; repeated measure ANOVA followed by Tukey test in C,D).

The average body weight was not significantly different between the 2K1C and sham operated groups (data not shown),but mean arterial pressure of 2K1C group was markedly higher than that of sham group at 4, 10, 12 weeks after the surgery (Figure 1A). The serum total cholesterol level remained at a low level (<100 mg/dl) for both groups throughout the experiment, and no difference was found between the groups, except for a slightly lower value in the 2K1C group on week 4 (Figure 1B). The average body weight among all groups was not significantly different (data not shown).

Increased mean arterial pressure was found in the 2K1C plus cholesterol-fed rabbits (Figure 1C), and markedly elevated serum total cholesterol levels (>200mg/dl) were found in both the hypercholesterolemia plus sham- and 2K1C plus hypercholesterolememia groups at 4, 10 and 12 weeks, compared to control rabbits on a normal diet (Figure 1D).

### 2. Microarray Analyses

#### 2.1. Microarray analyses of the hypertension only group

**Figure 2:**
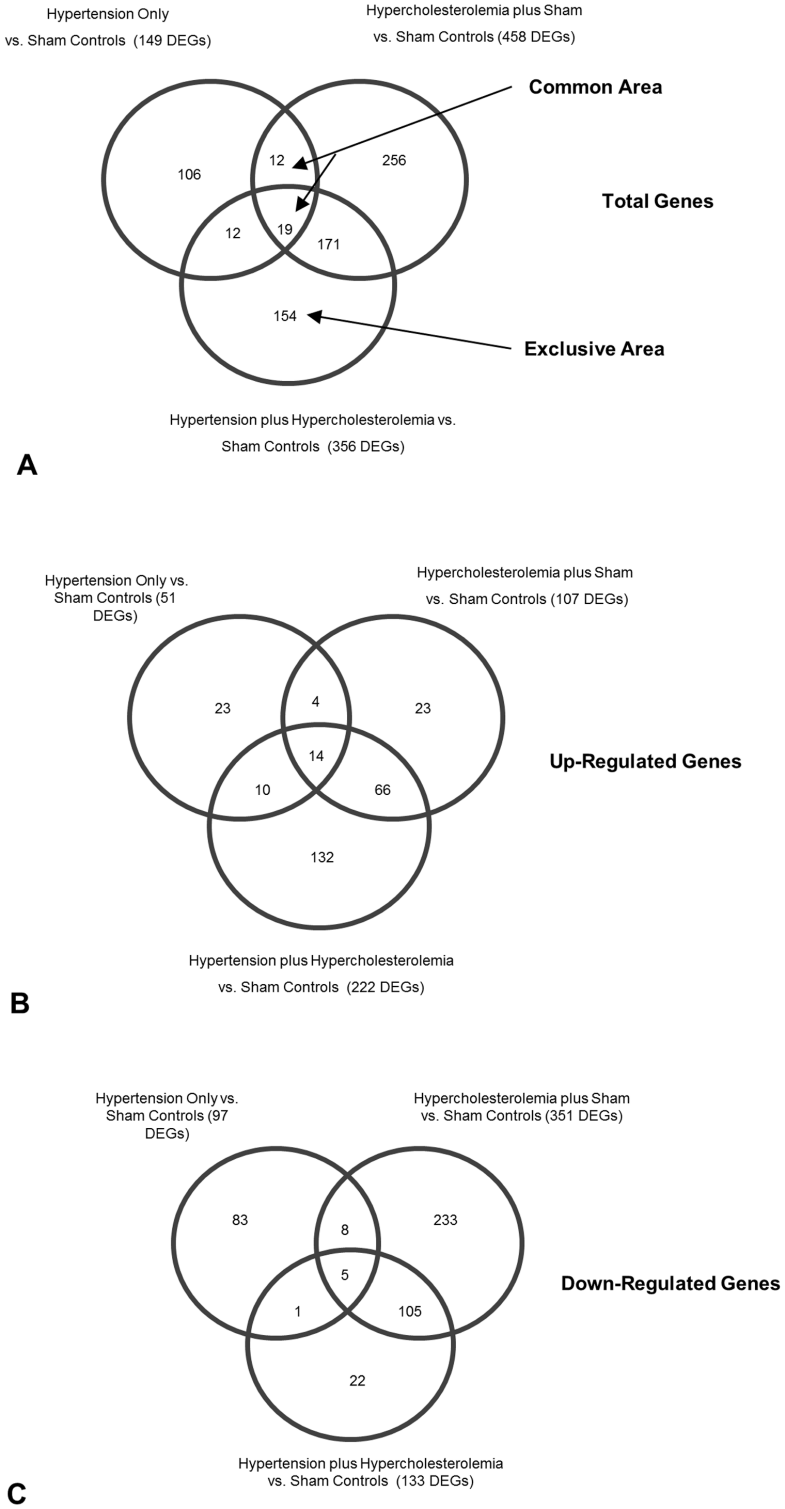
Venn diagram of DEGs in the MCA of hypertension only rabbits; hypercholesterolemia plus sham operated rabbits; and hypertension plus hypercholesterolemia rabbits; all vs. sham operated control rabbits. A: total number of genes, B: up-regulated genes C: down-regulated genes (One gene which is common between the Hypertension only- and Hypertension plus hypercholesterolemia group was both up- and down-regulated, and omitted).

The gene expression profile in the MCA of the hypertension only group was compared with that of sham operated controls on a normal diet. After unknown genes and repeated probes of the same genes were omitted, 51 up-regulated and 97 down-regulated genes (greater than 4-fold change) were found in the MCA (Figure 2). Among the highly up-regulated genes in the MCA of the hypertension only group compared to sham controls were FAM167A, CERS3 and FAM53C (Table 1). Among the highly down-regulated genes were FOXN1, NSRP1 and THUMPD3 (Table 2). The panel of genes was imported into IPA to analyze network interactions.

**Table 1:** Up-regulated genes in the MCA of ‘hypertension only’ rabbits vs. sham controls with greater than 4-fold change.

Gene	Gene Symbol	Corrected *p*-value	Hypertension only	Hyperchole-sterolemia plus sham	Hypertension plus hyperchole-sterolemia
			Fold Change	Fold Change	Fold Change
family with sequence similarity 167, member A	FAM167A	0.00017	14.04	2.44	5.11
ceramide synthase 3	CERS3	0.00486	11.44	−1.50	3.68
family with sequence similarity 53, member C	FAM53C	<0.00001	9.87	2.48	4.03
tubulin tyrosine ligase-like family, member 5	TTLL5	0.00005	8.16	−1.18	1.86
olfactory receptor, family 1, subfamily J, member 1	OR1J1	0.00002	8.14	3.83	−1.85
Interstitial collagenase Precursor (EC 3.4.24.7)(Matrix metalloproteinase-1)(MMP-1)	MMP1	0.00149	8.12	2.20	6.33
Nibrin-like	LOC100352398^∧^	0.00171	7.92	4.71	3.74
TAF15 RNA polymerase II, TATA box binding protein (TBP)-associated factor, 68kDa	TAF15^∧, +^	0.00004	7.45	4.71	4.65
zinc finger, DHHC-type containing 23	ZDHHC23	0.00001	7.18	3.22	5.64
ankyrin and armadillo repeat containing	ANKAR^∧, +^	0.00006	7.05	7.99	5.23
N(alpha)-acetyltransferase 25, NatB auxiliary subunit	NAA25^∧, +^	<0.00001	6.43	8.91	18.66
corticotropin releasing hormone binding protein	CRHBP	0.00005	6.38	2.23	4.02
Cytochrome P450 1A2 (EC 1.14.14.1)(CYPIA2)(Cytochrome P450 isozyme 4)(Cytochrome P450 LM4)(Cytochrome P450-PM4)	CYP1A2	0.00004	6.15	1.73	−1.90
KIAA0232	KIAA0232	0.00002	6.13	1.77	1.44
Fanconi anemia, complementation group C	FANCC^∧, +^	<0.00001	6.10	4.25	6.39
NEDD8 activating enzyme E1 subunit 1	NAE1	0.00009	6.10	3.26	−2.34
Zinc finger protein 638	LOC100345662	0.00005	6.01	1.02	−2.87
Uncharacterized protein C1orf50	C1orf50	0.00006	5.81	3.80	8.01
Mix paired-like homeobox	MIXL1	0.00093	5.80	3.54	4.64
Serine proteinase inhibitor, clade B, member 2 (Predicted)	SERPINB2^∧, +^	0.00005	5.63	6.99	9.10
IDI1 protein-like	LOC100346274^∧, +^	0.00042	5.51	4.87	6.67
B-cell receptor-associated protein 31	LOC100356247	0.00464	5.45	2.83	1.09
sperm associated antigen 6	SPAG6	0.00139	5.39	1.31	2.69
meprin A, beta	MEP1B^∧, +^	0.00291	5.37	5.12	5.85
G protein-coupled receptor kinase interacting ArfGAP 1	GIT1	<0.00001	5.22	2.76	3.91
ciliary rootlet coiled-coil, rootletin	CROCC	0.00003	5.08	2.33	2.79
SRY (sex determining region Y)-box 2-like	LOC100341629	0.00001	5.05	1.43	−7.56
Fibroblast growth factor binding protein 1-like	LOC100353835^∧, +^	0.00122	5.01	4.01	4.63
ankyrin repeat and SOCS box-containing 4	ASB4^∧^	0.00027	4.98	4.06	3.51
Double zinc ribbon and ankyrin repeat-containing protein 1	DZANK1^∧, +^	<0.00001	4.87	5.04	7.37
CD46 molecule, complement regulatory protein	CD46	0.00230	4.85	3.14	−1.11
programmed cell death 11	PDCD11	0.00003	4.85	−1.62	−1.11
signal-induced proliferation-associated 1 like 3	SIPA1L3^∧, +^	0.00001	4.80	8.45	6.50
relaxin/insulin-like family peptide receptor 2	RXFP2^∧, +^	0.00029	4.79	5.62	6.96
PHD and ring finger domains 1	PHRF1	0.00002	4.69	1.13	1.17
guanylate cyclase 2D, membrane (retina-specific)	GUCY2D^∧, +^	0.00008	4.64	4.11	8.34
EH domain binding protein 1	EHBP1	0.00171	4.60	2.08	−1.19
testis-specific kinase 2	TESK2^∧^	0.00571	4.50	5.11	2.41
Golgin subfamily B member 1	LOC100340420	0.00001	4.32	−2.30	−1.39
coiled-coil domain containing 54	CCDC54	0.00669	4.25	2.96	4.62
solute carrier family 39 (zinc transporter), member 12	SLC39A12	0.00006	4.23	2.48	4.96
l(3)mbt-like (Drosophila)	L3MBTL1	0.00011	4.21	1.80	1.38
chemokine (C-C motif) ligand 1	CCL1	<0.00001	4.18	1.13	1.37
ribonuclease, RNase A family, 1 (pancreatic)	RNASE1^∧, +^	<0.00001	4.17	5.62	4.82
DIS3 mitotic control homolog (S. cerevisiae)-like 2	DIS3L2^∧^	0.00024	4.17	4.32	3.96
Cold shock domain containing E1, RNA-binding	CSDE1	0.00002	4.16	1.89	2.99
antigen p97 (melanoma associated) identified by monoclonal antibodies 133.2 and 96.5 (MFI2)	MFI2	0.00386	4.04	2.47	1.09
Glucose-fructose oxidoreductase domain containing 2-like	LOC100351150^∧, +^	0.00001	4.01	6.34	12.42
coiled-coil domain containing 89	CCDC89	0.00238	4.01	1.64	3.00

^∧^Differentially expressed genes that are common between ‘hypertension only’ and ‘hypercholesterolemia plus sham’ groups (both vs. sham controls).^+^Differentially expressed genes that are common among ‘hypertension only’, ‘hypercholesterolemia plus sham’, and ‘hypertension plus hypercholesterolemia’ groups (all vs. sham controls).

**Table 2:** Down-regulated genes in the MCA of ‘hypertension only’ rabbits vs. sham controls with greater than 4-fold change.

Gene	Gene Symbol	Corrected *p*-value	Hypertension only	Hyperchole-sterolemia plus sham	Hypertension plus hyperchole-sterolemia
			Fold Change	Fold Change	Fold Change
forkhead box N1	FOXN1^∧, +^	0.00048	−26.20	−56.89	−45.12
nuclear speckle splicing regulatory protein 1	NSRP1	<0.00001	−24.13	−1.56	−1.23
THUMP domain containing 3	THUMPD3	<0.00001	−17.18	1.04	1.31
AT rich interactive domain 2 (ARID, RFX-like)	ARID2	<0.00001	−16.92	−2.86	−2.29
Ribosomal protein S3a-like	LOC100354966^∧^	<0.00001	−16.54	−5.74	−1.83
NADH dehydrogenase [ubiquinone] 1 alpha subcomplex assembly factor 5	NDUFAF5	0.00007	−15.92	−2.60	−1.44
ADAM metallopeptidase with thrombospondin type 1 motif, 17	ADAMTS17^∧^	0.00001	−15.85	−7.25	−3.06
peptidylprolyl isomerase G (cyclophilin G)	PPIG^∧^	<0.00001	−12.61	−6.20	−1.81
hematopoietic prostaglandin D synthase	HPGDS^∧, +^	0.00043	−12.38	−21.44	−13.63
cyclin H	CCNH	<0.00001	−10.89	−3.52	−1.34
LYR motif containing 7	LYRM7	0.00001	−10.41	−1.63	−1.65
male-specific lethal 2 homolog (Drosophila)	MSL2	0.00022	−9.72	−2.22	−2.10
secreted frizzled-related protein 4	SFRP4	<0.00001	−9.32	−1.07	−1.11
ankyrin 2, neuronal	ANK2	0.00002	−9.32	−1.39	−1.31
Ras-related protein Rab-7a	RAB7A	0.00001	−9.20	−1.18	−1.35
NLR family, pyrin domain containing 5	NLRP5	<0.00001	−8.60	1.51	2.02
Integrin-linked kinase-associated serine/threonine phosphatase	ILKAP	0.00001	−8.57	1.49	1.39
Histone H3.3B-like	LOC100341225	0.00209	−8.43	−1.36	−2.17
membrane-spanning 4-domains, subfamily A, member 1	MS4A1	0.00073	−7.86	−3.98	−2.53
sorting nexin 9	SNX9	0.00016	−7.82	−1.54	−1.78
protein tyrosine phosphatase, non-receptor type 23	PTPN23	0.00003	−7.80	1.69	1.30
large subunit GTPase 1 homolog (S. cerevisiae)	LSG1^∧^	<0.00001	−7.69	−20.38	−1.93
coiled-coil domain containing 59	CCDC59	<0.00001	−7.66	−2.29	−1.40
Protein SLX4IP	SLX4IP	0.00008	−7.64	−1.90	−1.79
ribosomal protein S12	RPS12	0.00079	−7.57	−1.56	−1.28
breast carcinoma amplified sequence 2	BCAS2	0.00001	−7.39	−1.24	−1.18
Secreted protein, acidic, cysteine-rich (osteonectin)	SPARC	0.00030	−7.26	−2.00	−1.62
mitochondrial ribosomal protein L15	MRPL15^∧, +^	<0.00001	−6.97	−23.01	−9.57
Translocase of inner mitochondrial membrane domain-containing protein 1	TIMMDC1	0.00021	−6.72	1.06	−1.07
olfactory receptor, family 4, subfamily D, member 2	OR4D2	0.00009	−6.61	−1.25	1.18
tumor necrosis factor, alpha-induced protein 8	TNFAIP8	0.00148	−6.59	−2.12	−2.30
LIM domains containing 1	LIMD1	0.00007	−6.10	−2.35	−1.61
Neurolysin, mitochondrial Precursor	NLN	0.00072	−6.05	−1.23	−1.39
Osteoglycin	OGN	0.00004	−5.96	−1.58	−1.49
Ribosomal protein L35-like	LOC100339185	0.00176	−5.85	−3.09	−1.69
transient receptor potential cation channel, subfamily V, member 5 (TRPV5)	TRPV5	0.00002	−5.80	−1.90	−1.49
Glycine cleavage system protein H (aminomethyl carrier)	GCSH	<0.00001	−5.68	−1.84	−1.61
translocase of outer mitochondrial membrane 5 homolog (yeast)	TOMM5^∧^	0.00014	−5.67	−4.05	−1.60
SEC24 family, member A (S. cerevisiae)	SEC24A	0.00205	−5.62	−2.86	−2.06
matrilin 2	MATN2	<0.00001	−5.55	1.73	1.69
ubiquitin specific peptidase 46	USP46	0.00039	−5.54	−2.52	−1.45
purine-rich element binding protein A	PURA	0.00034	−5.54	1.21	1.12
hydroxyprostaglandin dehydrogenase 15- (NAD)	HPGD	0.00366	−5.51	−2.30	−2.60
parkinson protein 7	PARK7	<0.00001	−5.44	1.27	1.07
IL2-inducible T-cell kinase	ITK	0.00003	−5.37	1.07	1.25
ribosomal protein L26	RPL26^∧^	0.00016	−5.27	−14.60	−2.81
endothelial PAS domain protein 1	EPAS1	0.00279	−5.24	−2.30	−1.70
ATP-sensitive inward rectifier potassium channel Kir6.2 (KIR62)	KIR62	0.00032	−5.21	−1.25	1.08
Kruppel-like factor 12	KLF12	0.00008	−5.14	−1.25	−1.15
TBC1 domain family, member 8B (with GRAM domain)	TBC1D8B	0.00101	−5.13	−1.47	−1.01
Nucleophosmin 1 isoform 1 (Predicted)	NPM1	0.00001	−5.12	−1.40	−1.40
BUD31 homolog (S. cerevisiae)	BUD31	0.00343	−5.08	−1.20	−1.30
poly(A) polymerase alpha	PAPOLA	<0.00001	−5.03	−2.29	−1.26
ribosomal protein L37a	RPL37A	<0.00001	−4.98	1.23	1.10
splicing factor 3a, subunit 3, 60 kDa	SF3A3	0.00003	−4.91	−1.55	−1.36
C1q and tumor necrosis factor related protein 1	C1QTNF1	0.00019	−4.90	−3.22	−1.22
family with sequence similarity 107, member B	FAM107B	0.00140	−4.88	−3.41	−2.58
integral membrane protein 2C	ITM2C	0.00015	−4.87	1.64	1.30
Alpha-1-B glycoprotein	A1BG	0.00121	−4.87	2.01	1.19
Histone deacetylase complex subunit SAP18-like	LOC100356364	0.00003	−4.86	−1.85	−1.65
phosphorylase kinase, beta (PHKB	PHKB	0.00033	−4.83	−1.23	−1.12
Vacuolar protein sorting-associated protein 51 homolog	VPS51	0.00004	−4.74	2.15	1.65
Uncharacterized protein C11orf57	C11orf57	0.00365	−4.65	−1.71	−1.25
exostoses (multiple)-like 3	EXTL3	0.00001	−4.65	−3.53	−4.65
Ferritin heavy chain Fragment (Ferritin H subunit)	FTH1	0.00003	−4.64	1.06	−1.09
Uncharacterized protein C15orf62, mitochondrial Precursor	C15orf62	0.00018	−4.58	1.21	1.48
lysine (K)-specific demethylase 4A	KDM4A	<0.00001	−4.57	2.48	2.70
NADH dehydrogenase (ubiquinone) 1 beta subcomplex, 6, 17kDa	NDUFB6	0.00116	−4.56	−1.76	−1.55
p21 protein (Cdc42/Rac)-activated kinase 4	PAK4	0.00002	−4.56	−1.16	−1.05
armadillo repeat containing, X-linked 3	ARMCX3	0.00161	−4.49	−1.40	−1.37
Transmembrane tyrosine kinase receptor	c-kit	0.00981	−4.48	−2.92	−2.29
leucine rich repeat (in FLII) interacting protein 2	LRRFIP2	0.00001	−4.47	1.23	1.14
cardiac calcium channel beta-subunit (CAB2)	CAB2	0.00008	−4.46	1.34	1.20
HERPUD family member 2	LOC100350332	0.00003	−4.42	−1.71	−1.80
family with sequence similarity 177, member A1	FAM177A1^∧^	0.00016	−4.41	−10.75	−1.95
acyl-CoA synthetase long-chain family member 4	ACSL4	0.00397	−4.40	−2.03	−1.94
interleukin-1 receptor-associated kinase 1 binding protein 1	IRAK1BP1	0.00001	−4.38	−2.94	−1.75
polyamine modulated factor 1 binding protein 1	PMFBP1	<0.00001	−4.34	2.06	2.81
Proteasome (prosome, macropain) 26s subunit, non-ATPase, 4	PSMD4^∧, +^	0.00005	−4.34	−17.67	−11.67
dpy-19-like 1 (C. elegans)	DPY19L1	<0.00001	−4.32	1.14	1.10
mastermind-like 2 (Drosophila)	MAML2	0.00134	−4.29	−1.57	1.05
choline phosphotransferase 1	CHPT1	0.00014	−4.26	−1.63	−1.47
Aggrecanase-2	ADAMTS-11^∧, +^	0.00005	−4.26	−9.31	−6.51
nuclear protein localization 4 homolog (S. cerevisiae)	NPLOC4	0.00342	−4.25	−2.86	−1.62
B-cell translocation gene 1, anti-proliferative	BTG1	0.00030	−4.24	−1.80	−1.81
galactokinase 2	GALK2	0.00002	−4.22	1.67	1.54
N(alpha)-acetyltransferase 50, NatE catalytic subunit	NAA50	0.00132	−4.21	−1.38	−1.99
Tubulin polyglutamylase complex subunit 2-like	LOC100353679	0.00044	−4.19	1.09	−1.02
Glycine N-methyltransferase Fragment (EC 2.1.1.20)	GNMT	0.00006	−4.17	−2.35	−1.18
Protein FAM210B	FAM210B	0.00130	−4.16	1.36	1.38
RNA pseudouridylate synthase domain containing 4 [	RPUSD4	0.00011	−4.11	1.05	1.04
nucleotide-binding oligomerization domain containing 2	NOD2	0.00111	−4.09	−1.39	−1.44
cysteine conjugate-beta lyase 2	CCBL2	0.00688	−4.07	−1.10	−1.03
BRCA2 and CDKN1A interacting protein	BCCIP^∧^	<0.00001	−4.06	−14.00	−2.58
major facilitator superfamily domain containing 9	MFSD9	0.00383	−4.04	−1.26	−1.52
Olfactory receptor 572 (Predicted)	OLFR572	0.00007	−4.03	1.30	2.27
neuroplastin	NPTN	0.00010	−4.02	−1.19	−1.19

^∧^ Differentially expressed genes that are common between ‘hypertension only’ and ‘hypercholesterolemia plus sham’ groups (both vs. sham controls).^+^Differentally expressed genes that are common among ‘hypertension only’, ‘hypercholesterolemia plus sham’, and ‘hypertension plus hypercholesterolemia’ groups (all vs. sham controls).

**Figure 3:**
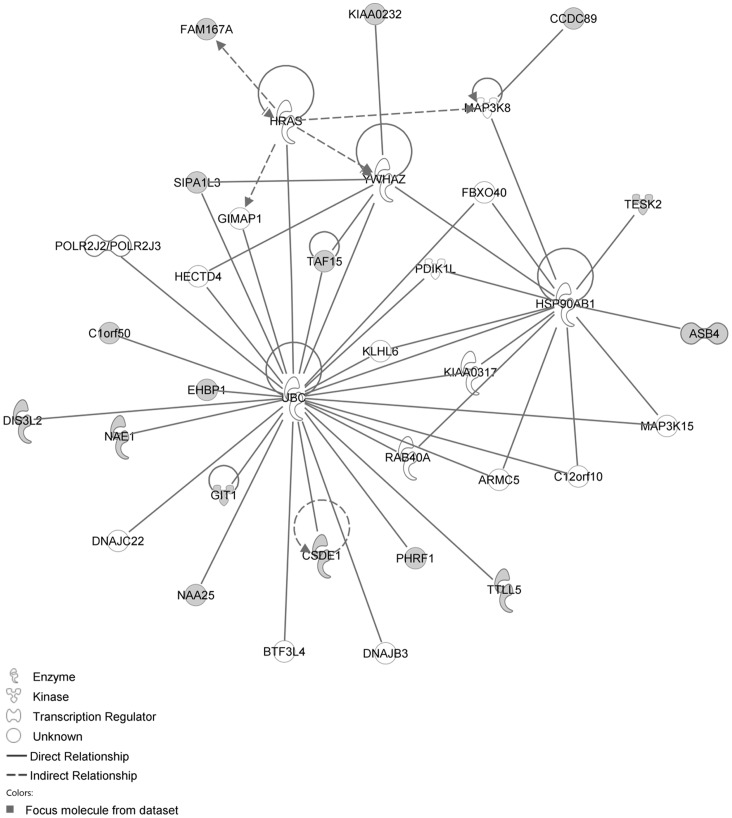
IPA network showing the network with the largest number of up-regulated focus genes in the MCA of the hypertension only group, compared with sham operated controls. Nodes are displayed using various shapes that represent functional classes of gene products. Focus genes in this network are indicated in grey nodes. Solid and dotted lines indicate direct and indirect interactions, respectively.

**Figure 4:**
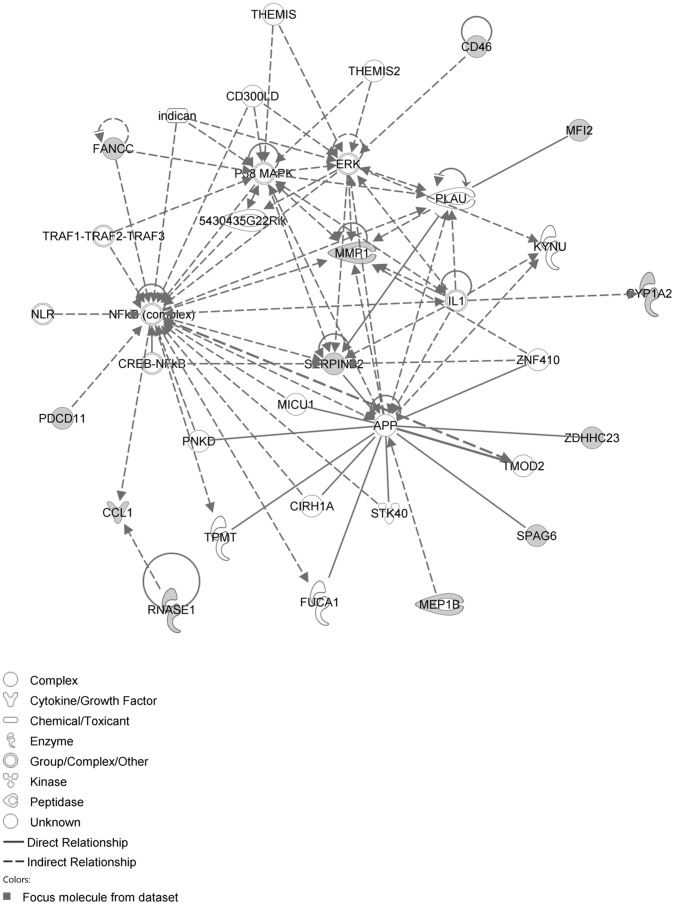
IPA network showing the network with the second largest number of up-regulated focus genes in the hypertension only group, compared with sham operated controls.

The IPA network with the ‘largest number of up-regulated focus genes’, contained 16 focus genes with functions in Cancer, Connective Tissue Disorders, Skeletal and Muscular Disorders. Focus genes in this network were ASB4, C1orf50, CCDC89, CSDE1, DIS3L2, EHBP1, FAM167A, GIT1, KIAA0232, NAA25, NAE1, PHRF1, SIPA1L3, TAF15, TESK2 and TTLL5. They were related to the ‘node molecule’, UBC (ubiquitin) (Figure 3, Table 1). The network with the second largest number of up-regulated focus genes had 12 focus genes, with functions in Cell-mediated Immune Response, Cellular Development, Cellular Function and Maintenance. Focus genes were CCL1, CD46, CYP1A2, FANCC, MEP1B, MFI2, MMP1, PDCD11, RNASE1, SERPINB2, SPAG6 and ZDHHC23; they were related to P38 MAPK, ERK, NFkB, SERPINB2, MMP1 and APP (Figure 4, Table 1).

**Figure 5:**
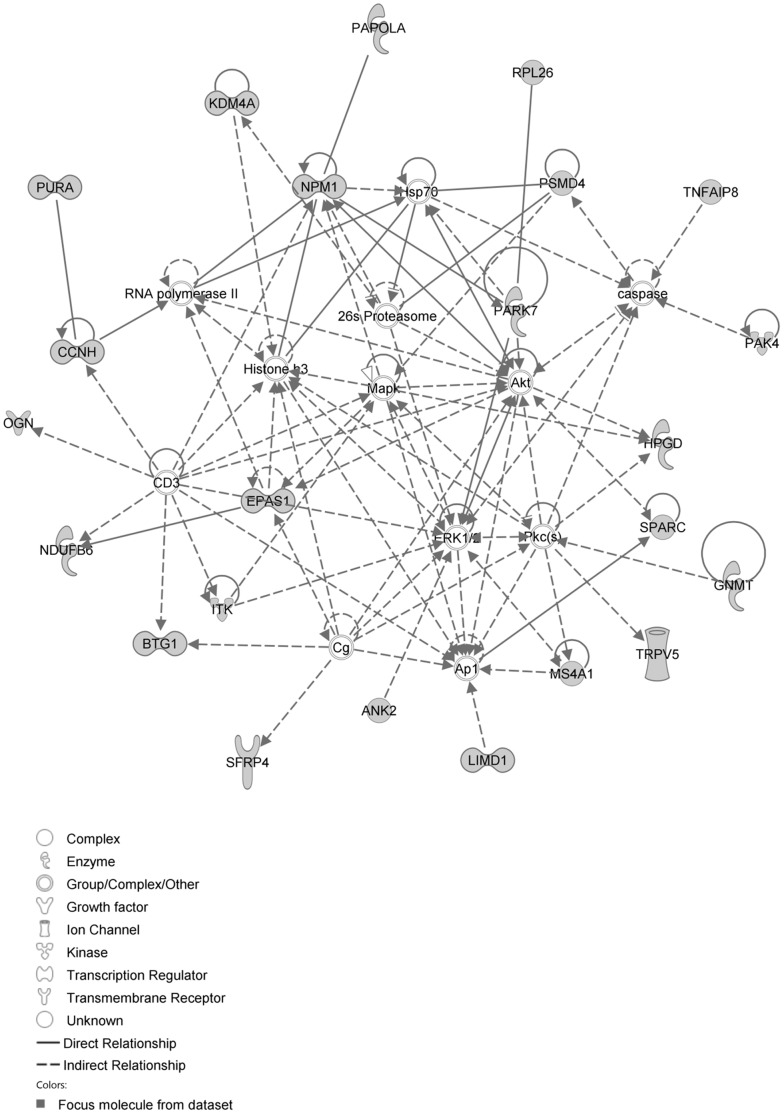
IPA network showing the network with the largest number of down-regulated focus genes in the hypertension only group, compared with sham operated controls.

**Figure 6:**
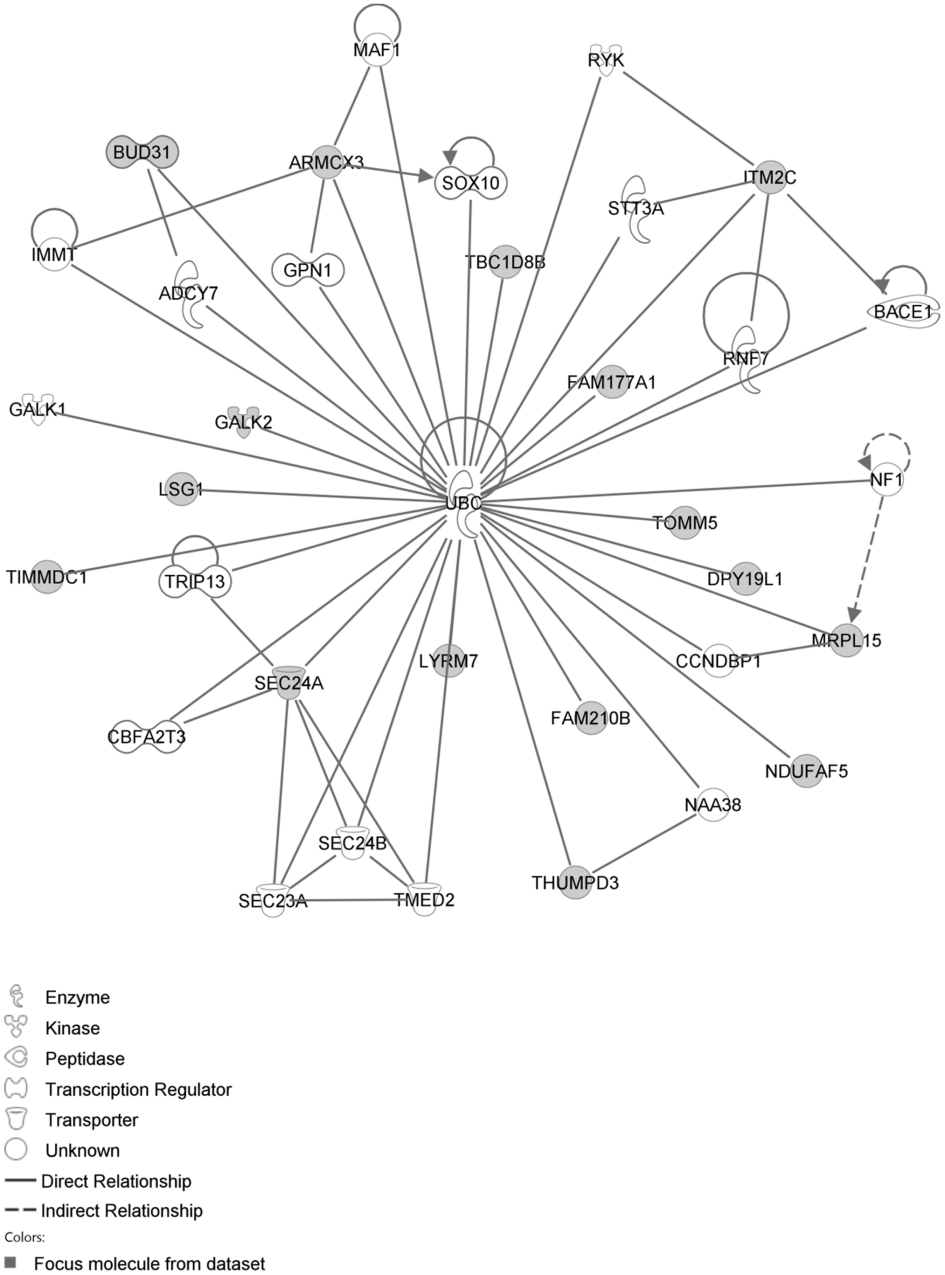
IPA network showing the network with the second largest number of down-regulated focus genes in the hypertension only group, compared with sham operated controls.

The network with the largest number of down-regulated focus genes contained 23 focus genes with functions in Cardiovascular System Development and Function, Organismal Development, Cell Morphology. Focus genes in this network were ANK2, BTG1, CCNH, EPAS1, GNMT, HPGD, ITK, KDM4A, LIMD1, MS4A1, NDUFB6, NPM1, OGN, PAK4, PAPOLA, PARK7, PSMD4, PURA, RPL26, SFRP4, SPARC, TNFAIP8 and TRPV5. They were related to MAPK, ERK1/2, Akt, 26s proteasome, histone H3 and PKC (Figure 5, Table 2). The network with the second largest number of down-regulated focus genes had 16 focus genes with functions in Cell Death and Survival, Embryonic Development, Cellular Development. Focus genes were ARMCX3, BUD31, DPY19L1, FAM177A1, FAM210B, GALK2, ITM2C, LSG1, LYRM7, MRPL15, NDUFAF5, SEC24A, TBC1D8B, THUMPD3, TIMMDC1 and TOMM5; they were related to UBC (ubiquitin)(Figure 6, Table 2).

#### 2.2. Microarray analyses of the hypercholesterolemia plus sham group

The gene expression profile in the MCA of the hypercholesterolemia plus sham group was compared with that of sham controls on a normal diet. After unknown and repeated genes were omitted, 107 up-regulated and 351 down-regulated genes (greater than 4-fold change) were found (Figure 2). Among the highly up-regulated genes in the MCA of the hypercholesterolemia plus sham group compared to sham controls were SLFN14, CA1, and LOC100357902 (Table 3). Among the highly down-regulated genes were LOC100125984, PFDN5 and CUL3 (Table 4).

**Table 3:** Up-regulated genes in the MCA of ‘hypercholesterolemia plus sham’ rabbits vs. sham controls with greater than 4-fold change.

Gene	Gene Symbol	Corrected *p*-value	Hyperchole-sterolemia plus sham	Hypertension only	Hypertension plus hyperchole-sterolemia
			Fold Change	Fold Change	Fold Change
schlafen family member 14	SLFN14	0.00565	14.04	2.01	16.33
carbonic anhydrase I	CA1	0.00531	10.02	2.38	10.07
Gap junction alpha-3 protein-like	LOC100357902	0.00689	9.62	2.92	9.96
EPH receptor A1	EPHA1	0.00008	8.98	3.03	20.31
N(alpha)-acetyltransferase 25, NatB auxiliary subunit	NAA25^∧, +^	<0.00001	8.91	6.43	18.66
transmembrane protein 212	TMEM212	0.00039	8.84	3.48	7.93
family with sequence similarity 46, member C	FAM46C	0.00016	8.51	1.72	6.92
signal-induced proliferation-associated 1 like 3	SIPA1L3^∧, +^	0.00001	8.45	4.80	6.50
SP110 nuclear body protein	SP110	<0.00001	8.42	2.06	15.47
Uncharacterized protein C11orf71	C11orf71	0.00446	8.33	2.97	9.22
ankyrin and armadillo repeat containing	ANKAR^∧,^^+^	0.00006	7.99	7.05	5.23
teneurin transmembrane protein 4	TENM4	0.00004	7.56	3.08	3.55
sterol O-acyltransferase 2	SOAT2	0.00003	7.18	3.01	5.94
Serine proteinase inhibitor, clade B, member 2 (Predicted)	SERPINB2^∧, +^	0.00005	6.99	5.63	9.10
Fc receptor-like A	FCRLA	0.00083	6.76	1.94	5.14
tetraspanin 33	TSPAN33	0.00078	6.37	3.97	5.53
Glucose-fructose oxidoreductase domain containing 2-like	LOC100351150^∧, +^	0.00001	6.34	4.01	12.42
CKLF-like MARVEL transmembrane domain containing 2	CMTM2	0.00071	6.28	2.38	7.79
solute carrier family 9 (sodium/hydrogen exchanger), member 8	SLC9A8	0.00175	6.14	3.55	10.32
spectrin, alpha, erythrocytic 1 (elliptocytosis 2)	SPTA1	0.00375	6.03	2.75	8.20
kinesin family member 13B	KIF13B	0.00002	6.02	2.35	9.02
aminolevulinate, delta-, synthase 2	ALAS2	0.00072	5.86	1.97	5.93
protease, serine, 38	PRSS38	0.00058	5.74	1.95	6.51
UHRF1 binding protein 1	UHRF1BP1	0.00074	5.70	2.63	4.69
Bardet-Biedl syndrome 5	LOC100342443	0.00004	5.62	2.76	8.15
relaxin/insulin-like family peptide receptor 2	RXFP2^∧, +^	0.00029	5.62	4.79	6.96
V-set and immunoglobulin domain containing 2	VSIG2	0.00168	5.53	3.51	4.60
REC8 homolog (yeast)	REC8	<0.00001	5.52	1.93	4.31
Gonadotropin-releasing hormone receptor	GnRHR	0.00008	5.47	3.11	10.39
transmembrane and coiled-coil domains 4	TMCO4	0.00007	5.42	2.43	4.28
Serine incorporator 1	LOC100357075	0.00015	5.37	2.50	4.74
transmembrane protein 173	TMEM173	0.00141	5.36	2.88	4.70
solute carrier family 9 (sodium/hydrogen exchanger), member 5	SLC9A5	0.00057	5.36	2.33	4.19
MLX interacting protein	MLXIP	0.00069	5.36	3.02	4.09
BCL2-like 10 (apoptosis facilitator)	BCL2L10	0.00018	5.34	1.67	4.64
follicle stimulating hormone receptor	FSHR	0.00023	5.31	1.24	8.27
zinc finger, CW type with PWWP domain 2	ZCWPW2	0.00011	5.29	2.31	5.21
Mitogen-activated protein kinase kinase kinase 4	MAP3K4	0.00196	5.19	3.17	4.93
PSMC3 interacting protein	PSMC3IP	0.00003	5.12	1.49	3.23
meprin A, beta	MEP1B^∧, +^	0.00291	5.12	5.37	5.85
testis-specific kinase 2	TESK2^∧^	0.00571	5.11	4.50	2.41
Double zinc ribbon and ankyrin repeat-containing protein 1	DZANK1^∧, +^	<0.00001	5.04	4.87	7.37
nuclear receptor subfamily 1, group D, member 1	NR1D1	0.00562	4.98	2.35	6.11
corin, serine peptidase	CORIN	0.00006	4.93	3.59	4.63
deltex homolog 3 (Drosophila)	DTX3	0.00013	4.92	3.47	4.56
IDI1 protein-like	LOC100346274^∧, +^	0.00042	4.87	5.51	6.67
RNA binding motif protein 6	RBM6	0.00003	4.85	1.73	6.15
TNF receptor-associated factor 3 interacting protein 1	TRAF3IP1	0.00043	4.80	1.29	5.56
TAF15 RNA polymerase II, TATA box binding protein (TBP)-associated factor, 68 kDa	TAF15^∧, +^	0.00004	4.71	7.45	4.65
Nibrin-like	LOC100352398^∧^	0.00171	4.71	7.92	3.74
LY6/PLAUR domain containing 5	LYPD5	0.00004	4.70	2.68	7.31
Aquaporin 1 (Colton blood group)	AQP1	0.00372	4.69	1.44	2.33
4-hydroxyphenylpyruvate dioxygenase-like	HPDL	0.00008	4.68	2.22	8.45
Mast cell-expressed membrane protein 1	MCEMP1	0.00003	4.67	1.74	8.27
amyloid beta (A4) precursor protein-binding, family B, member 3	APBB3	<0.00001	4.66	1.82	3.33
chymase like protein	LOC100008644	0.00009	4.58	1.84	8.36
transmembrane protease, serine 11E	TMPRSS11E	<0.00001	4.57	1.26	6.76
carboxypeptidase A5	CPA5	0.00007	4.56	3.29	9.11
Fc receptor-like 3	FCRL3	0.00022	4.52	3.78	-1.16
Carbonic anhydrase 2 (EC 4.2.1.1)	CA2	0.00964	4.51	1.67	4.68
pleckstrin homology domain containing, family G (with RhoGef domain) member 6	PLEKHG6	0.00011	4.47	3.23	9.24
BMS1-like, ribosome assembly protein	LOC100339194	0.00003	4.46	3.51	3.99
potassium channel tetramerisation domain containing 19	KCTD19	0.00004	4.40	1.51	6.56
G protein-coupled receptor 52	GPR52	0.00010	4.40	2.26	5.75
cyclin B3	CCNB3	0.00012	4.39	1.68	6.05
Cytochrome P450, family 4, subfamily A, polypeptide 5	CYP4A5	0.00044	4.39	3.27	6.74
kelch-like 17 (Drosophila)	KLHL17	<0.00001	4.38	2.72	3.65
WD repeat domain 13	WDR13	0.00063	4.37	2.31	3.14
O-sialoglycoprotein endopeptidase-like	LOC100348675	0.00039	4.36	3.87	3.71
Dmx-like 2	DMXL2	0.00657	4.36	1.23	3.79
CD2 molecule	CD2	0.00002	4.35	1.47	8.04
NADH dehydrogenase (ubiquinone) Fe-S protein 1, 75 kDa	LOC100341941	0.00022	4.34	2.69	5.46
DIS3 mitotic control homolog (S. cerevisiae)-like 2	DIS3L2^∧^	0.00024	4.32	4.17	3.96
general transcription factor IIE, polypeptide 2, beta 34 kDa	GTF2E2	0.00360	4.29	2.33	3.63
doublesex and mab-3 related transcription factor 2	DMRT2	0.00002	4.28	1.81	7.32
ribonuclease, RNase A family, 1 (pancreatic)	RNASE1^∧, +^	0.00010	4.26	3.23	3.15
Paralemmin 2	PALM2	0.00116	4.26	2.43	3.57
Fanconi anemia, complementation group C	FANCC^∧, +^	<0.00001	4.25	6.10	6.39
collagen, type VII, alpha 1	COL7A1	0.00600	4.25	2.00	2.98
Serine/threonine kinase 23, muscle-specific serine kinase 1 70 (Predicted)	SRPK3	0.00027	4.23	1.35	8.51
O-linked N-acetylglucosamine (GlcNAc) transferase (UDP-N-acetylglucosamine:polypeptide-N-acetylglucosaminyl transferase	OGT	<0.00001	4.20	1.98	3.65
SP100 nuclear antigen	SP100	0.00039	4.19	3.36	3.25
Uncharacterized protein C1orf51	C1orf51	0.00039	4.19	2.02	3.10
Islet amyloid polypeptide Precursor; Fragment (Amylin)	IAPP	0.00004	4.17	1.40	5.78
family with sequence similarity 19 (chemokine (C-C motif)-like), member A4	FAM19A4	0.00024	4.17	1.65	5.97
MORN repeat containing 4	MORN4	<0.00001	4.14	1.45	4.53
leucine-rich repeat-containing G protein-coupled receptor 5	LGR5	0.00019	4.14	3.32	5.36
spleen tyrosine kinase	SYK	0.00072	4.13	1.52	4.88
guanylate cyclase 2D, membrane (retina-specific)	GUCY2D^∧, +^	0.00008	4.11	4.64	8.34
Pregnancy-zone protein	PZP	0.00099	4.11	1.20	4.60
methyl-CpG binding domain protein 6	MBD6	<0.00001	4.11	2.26	3.11
TERF1 (TRF1)-interacting nuclear factor 2	TINF2	0.00011	4.10	3.47	6.41
ankyrin repeat and SOCS box-containing 4	ASB4^∧^	0.00027	4.06	4.98	3.51
tripartite motif-containing 65	TRIM65	0.00079	4.05	2.63	4.87
inhibitor of kappa light polypeptide gene enhancer in B-cells, kinase epsilon	IKBKE	0.00003	4.04	1.93	3.39
coiled-coil domain containing 54	CCDC54	0.00196	4.04	3.11	4.57
arylformamidase	AFMID	0.00100	4.03	1.65	5.81
3-oxo-5-alpha-steroid 4-dehydrogenase 2-like	LOC100343882	0.00403	4.02	2.74	3.56
Fibroblast growth factor binding protein 1-like	LOC100353835^∧, +^	0.00122	4.01	5.01	4.63
ATPase, Cu++ transporting, alpha polypeptide	ATP7A	0.00404	4.01	1.59	2.58
2,3-bisphosphoglycerate mutase (BPGM)	BPGM	0.00098	4.00	1.21	4.18

^∧^ Differentially expressed genes that are common between ‘hypertension only’ and ‘hypercholesterolemia plus sham’ groups (both vs. sham controls).^+^Differentially expressed genes that are common among ‘hypertension only’, ‘hypercholesterolemia plus sham’, and ‘hypertension plus hypercholesterolemia’ groups (all vs. sham controls).

**Table 4:** Down-regulated genes in the MCA of ‘hypercholesterolemia plus sham’ rabbits vs. sham controls with greater than 4-fold change.

Gene	GeneSymbol	Corrected *p*-value	Hyperchole-sterolemia plus sham	Hypertension only	Hypertension plus hyperchole-sterolemia
			Fold Change	Fold Change	Fold Change
beta tropomyosin (LOC100125984)	LOC100125984	<0.00001	−97.92	−2.30	−17.13
prefoldin subunit 5	PFDN5	<0.00001	−84.52	−1.78	−6.79
cullin 3	CUL3	<0.00001	−73.37	1.05	−6.10
family with sequence similarity 184, member B	FAM184B	<0.00001	−68.05	−1.32	−25.72
crystallin, alpha A (CRYAA)	CRYAA	<0.00001	−60.93	−1.50	−10.81
forkhead box N1	FOXN1^∧, +^	0.00048	−56.89	−26.20	−45.12
Tumor necrosis factor receptor superfamily, member 11b	TNFRSF11B	<0.00001	−55.91	1.69	−33.58
Glutamate dehydrogenase 1-like	LOC100351029	<0.00001	−55.82	1.18	−3.96
3′(2′), 5′-bisphosphate nucleotidase 1	BPNT1	<0.00001	−52.27	1.05	−25.01
interferon-related developmental regulator 1	IFRD1	<0.00001	−51.14	1.33	−4.37
manganese superoxide dismutase	SOD-2	<0.00001	−46.42	−1.23	−14.80
GC-rich promoter binding protein 1	GPBP1	<0.00001	−42.73	−2.55	−3.71
microtubule associated tumor suppressor 1	MTUS1	<0.00001	−41.71	−1.88	−20.40
ubiquinol-cytochrome c reductase, Rieske iron-sulfur polypeptide-like 1	UQCRFS1P1	<0.00001	−40.99	1.08	−23.74
Lupus La protein	SSB	<0.00001	−40.45	−1.63	−10.68
transmembrane protein 109 (TMEM109)	TMEM109	<0.00001	−39.14	−1.07	−4.26
Progesterone receptor membrane component 1	LOC100357097	<0.00001	−38.66	1.04	−21.91
Solute carrier family 9, subfamily A (NHE2, cation proton antiporter 2), member 2	SLC9A2	0.00007	−36.54	−2.62	−7.21
Obg-like ATPase 1	OLA1	<0.00001	−35.58	1.04	−2.87
Chaperonin containing TCP1, subunit 2 (beta)	CCT2	<0.00001	−35.56	−1.91	−30.96
androgen-induced 1	AIG1	0.00002	−35.48	−3.30	−14.63
endoplasmic reticulum lectin 1	ERLEC1	<0.00001	−34.54	1.35	−2.93
glyceraldehyde-3-phosphate dehydrogenase, spermatogenic	GAPDHS	<0.00001	−33.66	1.01	−32.91
transmembrane protein 38B	TMEM38B	<0.00001	−31.53	1.05	−18.80
Alpha-tubulin N-acetyltransferase	ATAT1	<0.00001	−29.43	1.37	−2.20
OTU domain containing 6A	OTUD6A	0.00011	−28.99	−3.59	−5.70
chaperonin containing TCP1, subunit 2 (beta) (CCT2)	CCT2	<0.00001	−28.67	−1.23	−4.57
Lysosomal-associated membrane protein 2-like	LOC100350946	<0.00001	−26.85	−1.78	−15.88
tumor protein, translationally-controlled 1 (TPT1)	TPT1	0.00008	−26.77	−1.45	−1.80
Tyrosine 3-monooxygenase/tryptophan 5-monooxygenase activation protein, beta polypeptide-like	LOC100358299	<0.00001	−26.07	−1.13	−3.37
AHA1, activator of heat shock 90kDa protein ATPase homolog 2 (yeast)	AHSA2	0.00081	−23.77	−3.66	−5.22
mitochondrial ribosomal protein L15	MRPL15^∧, +^	<0.00001	−23.01	−6.97	−9.57
zinc finger protein 638	ZNF638	<0.00001	−22.80	−1.10	−2.82
proteasome (prosome, macropain) 26s subunit, ATPase, 6	PSMC6	<0.00001	−22.71	−1.36	−3.80
v-Ki-ras2 Kirsten rat sarcoma viral oncogene homolog	KRAS	<0.00001	−22.11	−1.18	−15.47
myosin IE	MYO1E	<0.00001	−21.50	1.35	−2.84
hematopoietic prostaglandin D synthase	HPGDS^∧, +^	0.00043	−21.44	−12.38	−13.63
insulin induced gene 2	INSIG2	<0.00001	−21.07	−3.78	−4.71
Putative homeodomain transcription factor 2	PHTF2	<0.00001	−20.84	−1.69	−3.07
Cytoplasmic beta-actin	LOC100009506	<0.00001	−20.51	−1.19	−7.80
TRAF family member-associated NFKB activator	TANK	0.00001	−20.39	−1.28	−5.66
zinc finger protein 642	ZFP69	0.00058	−20.24	−1.58	−12.02
SWI/SNF related, matrix associated, actin dependent regulator of chromatin, subfamily a, member 5	SMARCA5	<0.00001	−19.50	1.08	−8.08
Transmembrane protein 14C-like	LOC100357487	0.00875	−18.42	1.43	−1.74
zinc finger protein 326	ZNF326	<0.00001	−18.36	1.09	−6.29
DCN1, defective in cullin neddylation 1, domain containing 4 (S. cerevisiae)	DCUN1D4	<0.00001	−18.27	−1.65	−15.90
PHD finger protein 6	PHF6	0.00001	−17.91	−1.84	−3.80
CD302 molecule	CD302	<0.00001	−17.51	−1.14	−4.14
ORM1-like 3 (S. cerevisiae)	ORMDL3	<0.00001	−17.49	−1.24	−16.75
large subunit GTPase 1 homolog (S. cerevisiae)	LSG1^∧^	0.00001	−17.14	1.16	−2.15
FGGY carbohydrate kinase domain containing	FGGY	0.00007	−16.26	−2.04	−3.77
Eukaryotic translation initiation factor 2 subunit 1	EIF2S1	0.00001	−16.08	−2.04	−14.40
ribophorin I	RPN1	0.00002	−15.81	−1.30	−4.51
periphilin 1	PPHLN1	<0.00001	−15.71	1.14	−2.19
mitochondrial ribosomal protein L1	MRPL1	<0.00001	−15.71	−1.06	−12.00
tyrosine 3-monooxygenase/tryptophan 5-monooxygenase activation protein, theta polypeptide (YWHAQ)	YWHAQ	<0.00001	−15.13	−1.44	−2.31
Sin3A-associated protein, 30 kDa	SAP30	<0.00001	−14.99	−1.09	−16.96
ribosomal protein L26	RPL26^∧^	0.00016	−14.60	−5.27	−2.81
amyloid beta precursor protein (cytoplasmic tail) binding protein 2	APPBP2	<0.00001	−14.30	−1.11	−2.02
nucleolar protein 10	NOL10	<0.00001	−14.02	1.08	−5.15
TIA1 cytotoxic granule-associated RNA binding protein-like 1	TIAL1	0.00001	−13.80	2.34	−4.88
Titin	TTN	0.00001	−13.60	−2.26	−6.90
asparagine-linked glycosylation 9, alpha-1,2-mannosyltransferase homolog (S. cerevisiae)	ALG9	<0.00001	−13.51	−1.33	−2.12
Aryl hydrocarbon receptor nuclear translocator (ARNT protein)(Dioxin receptor, nuclear translocator)(Hypoxia-inducible factor 1 beta)(HIF-1 beta)	ARNT	<0.00001	−13.43	1.04	−5.12
BRCA2 and CDKN1A interacting protein	BCCIP^∧^	<0.00001	−13.26	−1.00	−2.51
WW domain containing adaptor with coiled-coil	WAC	0.00001	−13.20	−3.12	−11.91
Uncharacterized LOC100347346	LOC100347346	0.00002	−13.05	1.33	−2.30
Tweety homolog 1 (Drosophila)	TTYH1	0.00108	−12.82	−5.16	−27.02
ubiquitin specific peptidase 16	USP16	<0.00001	−12.55	1.16	−1.82
UDP-glucuronosyltransferase	UGT2C1	<0.00001	−12.36	2.01	−2.07
Heterogeneous nuclear ribonucleoprotein C (hnRNP C)	HNRNPC	<0.00001	−12.22	−1.33	−7.49
Nucleolar protein 11	NOL11	0.00032	−11.98	2.09	−13.01
guanylate cyclase 1, soluble, beta 3	GUCY1B3	0.00010	−11.97	−2.52	−6.79
SWI/SNF related, matrix associated, actin dependent regulator of chromatin, subfamily d, member 3	SMARCD3	<0.00001	−11.91	−1.18	−2.86
up-regulated during skeletal muscle growth 5 homolog (mouse)	USMG5	<0.00001	−11.86	−1.47	−2.36
endothelial differentiation gene 7 protein	LOC100009484	0.00562	−11.73	−1.86	−7.39
cysteine-rich protein 3	CRIP3	<0.00001	−10.99	1.45	−4.26
interferon induced with helicase C domain 1	IFIH1	0.00001	−10.96	1.21	−5.60
Solute carrier family 2, facilitated glucose transporter member 3 Fragment (Glucose transporter type 3, brain)(GLUT-3)	SLC2A3	0.00019	−10.95	−1.81	−11.60
eukaryotic translation initiation factor 3, subunit M	EIF3M	<0.00001	−10.81	−1.38	−2.09
PDLIM1 interacting kinase 1 like	PDIK1L	0.00010	−10.76	−2.49	−2.61
F-box and WD repeat domain containing 7	FBXW7	<0.00001	−10.71	1.36	−2.36
IQ motif containing GTPase activating protein 3	IQGAP3	0.00007	−10.54	1.25	−9.37
caudal type homeobox 1	CDX1	0.00001	−10.42	−1.18	−6.08
RUN and FYVE domain containing 1	RUFY1	0.00004	−10.38	−1.02	−2.46
lysine (K)-specific demethylase 4A	KDM4A	<0.00001	−10.29	−2.03	−3.47
RWD domain containing 3	RWDD3	<0.00001	−10.16	1.03	−2.04
syntrophin, alpha 1 (dystrophin-associated protein A1, 59 kDa, acidic component) (SNTA1)	SNTA1	<0.00001	−9.97	−1.04	−5.98
secretion regulating guanine nucleotide exchange factor	SERGEF	0.00011	−9.83	−1.08	−1.71
dehydrogenase/reductase (SDR family) member 13	DHRS13	<0.00001	−9.82	1.02	−4.23
SWI/SNF related, matrix associated, actin dependent regulator of chromatin, subfamily a, member 1	SMARCA1	<0.00001	−9.77	1.18	−2.16
RIO kinase 2	LOC100347237	<0.00001	−9.56	−1.53	−3.14
PDZD4 protein (Predicted)	PDZD4	0.00005	−9.45	−1.47	−3.15
adaptor-related protein complex 4, sigma 1 subunit	AP4S1	<0.00001	−9.37	−1.09	−2.92
neurocalcin delta	NCALD	0.00037	−9.22	−1.93	−6.13
Bcl2 modifying factor	BMF	<0.00001	−9.18	−1.04	−4.55
signal recognition particle 54 kDa	SRP54	<0.00001	−9.16	1.22	−1.88
Cytochrome P450, family 7, subfamily A, polypeptide 1	CYP7A1	<0.00001	−9.16	1.93	−3.27
ring finger protein 38	RNF38	0.00023	−9.14	−3.33	−4.93
Zinc finger protein 800 (Predicted)	ZNF800	0.00004	−9.12	−1.81	−2.07
DnaJ (Hsp40) homolog, subfamily B, member 4	DNAJB4	<0.00001	−9.11	−1.86	−4.76
claudin 16	CLDN16	0.00014	−9.09	−1.23	−3.61
WW domain containing E3 ubiquitin protein ligase 1	WWP1	<0.00001	−9.02	−1.11	−2.33
Actin, alpha skeletal muscle (Alpha-actin-1)	ACTA1	0.00001	−8.93	−1.11	−3.08
TANK-binding kinase 1	TBK1	0.00002	−8.82	−1.66	−2.80
IQ motif and Sec7 domain 3	IQSEC3	0.00193	−8.73	−2.18	−3.18
karyopherin alpha 4 (importin alpha 3)	KPNA4	<0.00001	−8.63	−1.27	−1.92
MACRO domain containing 2	MACROD2	0.00003	−8.51	−1.57	−2.19
transmembrane protein 70	TMEM70	0.00001	−8.48	−1.36	−3.70
SRY (sex determining region Y)-box 5	SOX5	0.00036	−8.38	−1.20	−2.75
ring finger protein 222	RNF222	0.00001	−8.36	−1.78	−6.02
Annexin A2	LOC100357671	0.00008	−8.32	1.11	−3.60
enabled homolog (Drosophila)	ENAH	0.00036	−8.30	1.27	−2.87
cell division cycle 37 homolog (S. cerevisiae)-like 1	CDC37L1	0.00001	−8.25	−2.44	−3.73
zinc finger RNA binding protein	ZFR	<0.00001	−8.22	−1.06	−2.45
protein kinase C, beta (PRKCB), transcript variant II	PRKCB	0.00026	−8.21	3.41	−3.02
tetratricopeptide repeat domain 21B	TTC21B	<0.00001	−8.19	1.01	−3.64
shroom family member 3	SHROOM3	<0.00001	−7.95	−3.82	−4.60
phospholamban (LOC100009299)	PLN	0.00002	−7.94	−2.00	−4.25
Thioredoxin interacting protein	TXNIP	0.00017	−7.93	−1.22	−2.37
Destrin	LOC100358081	<0.00001	−7.84	−1.07	−2.32
Programmed cell death 6 interacting protein	LOC100341540	0.00018	−7.83	−1.88	−3.78
DEAH (Asp-Glu-Ala-His) box polypeptide 9	DHX9	<0.00001	−7.81	1.34	−2.39
MAX dimerization protein 1	MXD1	0.00035	−7.73	−1.51	−3.29
Acidic (leucine-rich) nuclear phosphoprotein 32 family, member E	LOC100349957	0.00032	−7.65	−1.50	−2.24
protein kinase N2	PKN2	0.00001	−7.59	−1.47	−3.08
5′-3′ exoribonuclease 1	XRN1	0.00029	−7.58	−1.69	−2.98
NADP-dependent oxidoreductase domain-containing protein 1	NOXRED1	0.00140	−7.55	−3.36	−3.35
adenosine deaminase, tRNA-specific 2, TAD2 homolog (S. cerevisiae)	ADAT2	0.00032	−7.55	1.09	−2.38
NADH dehydrogenase-like	LOC100347823	<0.00001	−7.55	2.02	−7.93
Cold shock domain containing E1, RNA-binding	CSDE1	0.00001	−7.53	−2.74	−3.37
IQ motif containing E	IQCE	<0.00001	−7.52	1.75	−5.18
POU class 2 homeobox 3	POU2F3	<0.00001	−7.48	1.89	−4.78
H6 family homeobox 2	HMX2	0.00121	−7.46	−1.58	−1.86
Kruppel-like factor 1 (erythroid)	KLF1	<0.00001	−7.43	1.07	−3.10
AP2 associated kinase 1	AAK1	0.00571	−7.42	−2.56	−3.14
zinc finger protein 330	ZNF330	0.00012	−7.41	−1.48	−8.54
NudC domain containing 2	NUDCD2	<0.00001	−7.37	−1.41	−2.72
spermatogenesis associated 24	SPATA24	0.00015	−7.37	−1.43	−3.75
GC-rich sequence DNA-binding factor candidate isoform 1 (Predicted)	RA_m005_jsm426FEr	0.00019	−7.35	1.03	−1.46
gap junction protein, delta 3, 31.9 kDa	GJD3	<0.00001	−7.33	1.35	−4.40
zinc finger, MYM-type 1	ZMYM1	0.00017	−7.33	−1.60	−3.17
cytochrome P450 IIC16 mRNA (CYP2C16)	CYP2C16	<0.00001	−7.31	2.43	−3.15
ASF1 anti-silencing function 1 homolog A (Predicted)	ASF1A	<0.00001	−7.31	−1.11	−2.33
protein phosphatase 1, regulatory (inhibitor) subunit 8	PPP1R8	<0.00001	−7.27	1.09	−4.24
ADAM metallopeptidase with thrombospondin type 1 motif, 17	ADAMTS17^∧^	0.00001	−7.25	−15.85	−3.06
myosin, light chain 6B, alkali, smooth muscle and non-muscle	MYL6B	0.00002	−7.23	−1.58	−4.66
bone morphogenetic protein 7 (bmp7)	BMP7	<0.00001	−7.23	−1.14	−2.05
clathrin, light chain A	CLTA	<0.00001	−7.22	−1.05	−2.84
pancreatic polypeptide receptor 1 (PPYR1)	PPYR1	0.00003	−7.22	−1.10	−4.12
sal-like 1 (Drosophila)	SALL1	<0.00001	−7.20	−1.03	−3.00
Crystallin, zeta-like 1 (Predicted)	CRYZL1	<0.00001	−7.20	−1.58	−2.86
junctional adhesion molecule 2	JAM2	0.00121	−7.11	−2.54	−3.40
potassium channel tetramerisation domain containing 15	KCTD15	<0.00001	−7.11	−1.16	−3.93
WW domain binding protein 4 (formin binding protein 21)	WBP4	<0.00001	−7.08	−1.63	−2.77
actin, alpha 1, skeletal muscle	ACTA1	0.00003	−7.04	−1.28	−2.10
host cell factor C1 regulator 1 (XPO1 dependent)	HCFC1R1	0.00001	−6.97	−1.52	−3.01
integrin, beta 8 (ITGB8)	ITGB8	0.00098	−6.93	−1.98	−2.79
OTU domain containing 7A	OTUD7A	<0.00001	−6.87	−1.30	−2.70
THAP domain containing 6	THAP6	0.00024	−6.86	−2.75	−2.73
sialic acid binding Ig-like lectin 14	SIGLEC14	<0.00001	−6.80	1.14	−4.41
nucleolar protein 8	NOL8	0.00005	−6.74	−1.62	−2.14
epithelial cell transforming sequence 2 oncogene	ECT2	0.00003	−6.73	1.12	−2.57
WD repeat domain 61	WDR61	0.00029	−6.72	−1.07	−1.47
Ribosomal protein L7-like	LOC100339887	0.00001	−6.70	−1.23	−5.10
kinesin family member 20A	KIF20A	0.00003	−6.68	−2.21	−5.16
ELOVL family member 5, elongation of long chain fatty acids (FEN1/Elo2, SUR4/Elo3-like, yeast)	ELOVL5	<0.00001	−6.66	−1.34	−2.32
phospholipase A2, group IVC (cytosolic, calcium-independent)	PLA2G4C	0.00006	−6.55	−1.01	−3.36
complement component 1, q subcomponent-like 3	C1QL3	0.00293	−6.49	−2.42	−6.91
ribosomal protein L3-like	RPL3L	<0.00001	−6.49	1.11	−2.23
actin, beta-like 2	ACTBL2	0.00008	−6.44	−3.04	−2.71
proteolipid protein 1 (PLP1)	PLP1	0.00339	−6.44	−1.79	−6.50
DnaJ (Hsp40) homolog, subfamily C, member 6	DNAJC6	0.00010	−6.43	−2.42	−6.45
zinc finger and BTB domain containing 44	ZBTB44	0.00001	−6.43	−1.92	−2.58
poliovirus receptor-related 3	PVRL3	<0.00001	−6.30	1.08	−2.09
protein phosphatase, EF-hand calcium binding domain 1	PPEF1	0.00008	−6.28	−1.45	−2.25
Acidic ribosomal phosphoprotein P0	36B4	0.00003	−6.27	1.43	−4.94
peptidylprolyl isomerase G (cyclophilin G)	PPIG^∧^	<0.00001	−6.20	−12.61	−1.81
mediator complex subunit 19	MED19	0.00002	−6.16	−1.27	−2.30
complement component 1, r subcomponent-like	C1RL	<0.00001	−6.15	1.42	−1.33
homeobox C11	HOXC11	0.00026	−6.13	−1.10	−3.83
Cell division cycle 37 protein	LOC100344117	0.00041	−6.12	1.19	−3.81
glycine-N-acyltransferase-like 3	GLYATL3	0.00001	−6.10	−1.23	−2.57
family with sequence similarity 177, member A1	FAM177A1^∧^	<0.00001	−6.05	−1.32	−1.70
poly(rC) binding protein 3	PCBP3	0.00109	−6.03	−1.27	−3.94
KIAA0305-like	LOC100358165	<0.00001	−6.02	−1.11	−1.64
dymeclin	DYM	<0.00001	−5.97	−1.57	−3.07
inositol polyphosphate phosphatase-like 1	INPPL1	0.00005	−5.96	−1.14	−4.87
enolase 2 (gamma, neuronal)	ENO2	<0.00001	−5.92	1.21	−2.24
bactericidal/permeability-increasing protein-like 3	BPIFB6	0.00001	−5.92	−1.79	−6.83
klotho (KL)	KL	0.00379	−5.89	−2.06	−2.74
ELMO/CED-12 domain containing 2	ELMOD2	0.00024	−5.87	−2.02	−2.22
Proteasome (prosome, macropain) 26s subunit, non-ATPase, 4	PSMD4^∧, +^	<0.00001	−5.84	−1.49	−9.66
pleckstrin homology domain interacting protein	PHIP	0.00039	−5.84	−2.72	−3.73
NCK-associated protein 1	NCKAP1	<0.00001	−5.79	−1.16	−1.71
ATPase family, AAA domain containing 2	ATAD2	0.00002	−5.78	1.13	−2.53
low density lipoprotein-related protein 1 (LRP1)	LRP1	0.00009	−5.78	−1.98	−2.03
ISL LIM homeobox 2	ISL2	0.00007	−5.75	−1.36	−4.24
cell adhesion molecule 1	CADM1	0.00002	−5.75	1.10	−3.03
Ribosomal protein S3a-like	LOC100354966^∧^	<0.00001	−5.74	−16.54	−1.83
Filamin A, alpha	FLNA	<0.00001	−5.73	1.88	−3.35
integrin, alpha 10	ITGA10	0.00014	−5.71	−1.72	−2.44
SERTA domain containing 4	SERTAD4	0.00006	−5.69	1.01	−2.01
Poly(A) binding protein, cytoplasmic 4 (inducible form)	PABPC4	0.00009	−5.67	1.10	−4.16
immunoglobulin superfamily, member 9	IGSF9	0.00223	−5.66	−2.79	−3.20
guanine nucleotide binding protein (G protein), gamma 3	GNG3	<0.00001	−5.64	1.07	−4.14
nerve growth factor receptor	NGFR	<0.00001	−5.62	2.10	−3.05
Intraflagellar transport 140-like	LOC100355837	<0.00001	−5.61	3.58	−8.45
Ribosomal protein L12-like	LOC100353722	0.00007	−5.60	−1.03	−3.99
ring finger and CCCH-type zinc finger domains 2	RC3H2	<0.00001	−5.59	1.15	−1.86
Small proline-rich protein 3	SPRR3	<0.00001	−5.59	2.41	−1.85
hypoxia inducible factor 1, alpha subunit (basic helix-loop-helix transcription factor) (HIF1A)	HIF1A	0.00001	−5.59	1.16	−2.44
ATG4 autophagy related 4 homolog C (S. cerevisiae)	ATG4C	0.00003	−5.54	−1.12	−1.29
XK, Kell blood group complex subunit-related, X-linked	XKRX	0.00006	−5.51	−1.43	−1.35
ankyrin repeat and SOCS box-containing 10	ASB10	<0.00001	−5.49	−1.64	−2.80
aminoadipate aminotransferase	AADAT	0.00005	−5.48	−1.41	−1.78
zinc finger, ZZ-type containing 3	ZZZ3	0.00007	−5.44	−1.49	−2.11
transgelin 3	TAGLN3	0.00003	−5.41	−1.12	−1.93
SWI/SNF-related, matrix-associated actin-dependent regulator of chromatin, subfamily a, containing DEAD/H box 1	SMARCAD1	<0.00001	−5.41	1.04	−2.04
actinin, alpha 2	ACTN2	0.00001	−5.36	1.37	−3.35
synaptotagmin XII	SYT12	0.00001	−5.36	1.14	−6.91
UDP glycosyltransferase 8	UGT8	0.00127	−5.36	−1.08	−2.71
RasGEF domain family, member 1A	RASGEF1A	0.00001	−5.35	1.29	−2.64
hematopoietic SH2 domain containing	HSH2D	0.00001	−5.31	−3.19	−3.85
3-hydroxyisobutyrate dehydrogenase Fragment (HIBADH)(EC 1.1.1.31)	HIBADH	0.00001	−5.27	1.30	−2.30
Vacuolar protein sorting 4 homolog A (S. cerevisiae)	VPS4A	0.00028	−5.27	−1.17	−3.45
Synaptosomal-associated protein 25	LOC100340057	0.00007	−5.26	1.05	−2.54
MAPK scaffold protein 1	LAMTOR3	0.00002	−5.24	1.03	−2.10
Ellis van Creveld syndrome	EVC	0.00258	−5.24	−1.67	−3.16
topoisomerase (DNA) II beta 180 kDa	TOP2B	<0.00001	−5.23	−1.03	−1.58
RAB23, member RAS oncogene family	RAB23	0.00066	−5.20	−2.40	−2.59
N-myristoyltransferase 2	NMT2	0.00005	−5.19	1.14	−1.51
transmembrane emp24 protein transport domain containing 4	TMED4	0.00077	−5.19	−1.21	−3.23
general transcription factor IIF, polypeptide 2, 30 kDa	GTF2F2	0.00007	−5.17	−1.26	−1.92
ribosomal protein L32	RPL32	0.00002	−5.17	−1.92	−1.46
syntaxin 2	STX2	0.00003	−5.16	−1.46	−2.73
succinate-CoA ligase, alpha subunit	SUCLG1	<0.00001	−5.14	−1.02	−1.93
activating transcription factor 1	ATF1	0.00020	−5.14	−2.13	−3.43
serum amyloid protein A (LOC100009259)	LOC100009259	0.00151	−5.09	1.30	−3.10
peroxisomal biogenesis factor 1	PEX1	<0.00001	−5.06	−1.07	−1.71
interleukin-1 receptor-associated kinase 1 binding protein 1	IRAK1BP1	<0.00001	−5.06	−1.31	−2.74
ubiquitin specific peptidase 3	USP3	0.00011	−5.06	−1.16	−2.32
BEN domain containing 6	BEND6	0.00046	−5.04	−1.29	−3.94
Ribosomal protein S14-like	LOC100343884	0.00002	−5.03	1.67	−2.65
HNF1 homeobox B	HNF1B	0.00233	−5.02	−1.62	−3.13
Ribosomal protein S7-like	LOC100345715	0.00012	−4.96	−1.91	−4.17
GTPase activating protein (SH3 domain) binding protein 2	G3BP2	<0.00001	−4.96	−1.63	−2.98
insulin induced gene 1	INSIG1	0.00148	−4.93	−1.34	−3.58
discs, large homolog 2 (Drosophila)	DLG2	0.00140	−4.92	−1.24	−3.97
syntrophin, gamma 1	SNTG1	0.00002	−4.90	−1.42	−3.33
cell division cycle 73, Paf1/RNA polymerase II complex component, homolog (S. cerevisiae)	CDC73	0.00714	−4.89	−1.83	−2.18
family with sequence similarity 26, member F	FAM26F	0.00573	−4.88	−1.47	−2.67
galactoside 2-L-fucosyltransferase (RFT-I)	RFT-I	0.00014	−4.85	−1.47	−2.96
Ribosomal protein L36a-like	LOC100358069	0.00001	−4.82	−1.04	−4.28
microtubule associated monoxygenase, calponin and LIM domain containing 1	MICAL1	0.00010	−4.81	−1.87	−4.30
Signal peptidase complex subunit 2 homolog	LOC100339691	<0.00001	−4.81	1.37	−3.46
Eukaryotic translation initiation factor 1	LOC100345195	0.00015	−4.81	−1.22	−4.44
Poly(rC) binding protein 2-like	LOC100357382	<0.00001	−4.79	1.04	−2.27
kynureninase (L-kynurenine hydrolase)	KYNU	0.00143	−4.73	1.09	−2.92
glutamate receptor, ionotropic, N-methyl D-aspartate-like 1B	GCOM2	0.00001	−4.72	−1.28	−1.74
NADH dehydrogenase (ubiquinone) Fe-S protein 5, 15 kDa (NADH-coenzyme Q reductase)	LOC100342714	0.00018	−4.70	1.30	−3.33
Signal sequence receptor, beta-like	LOC100342387	0.00006	−4.66	1.96	−3.48
family with sequence similarity 107, member B	FAM107B	0.00026	−4.66	−1.36	−2.73
Kinesin family member 5B-like	LOC100352693	0.00002	−4.64	1.24	−3.27
tRNA aspartic acid methyltransferase 1	TRDMT1	0.00300	−4.63	−1.58	−2.39
prostaglandin reductase 2	PTGR2	0.00345	−4.62	−3.40	−2.54
zinc finger CCCH-type containing 7A	ZC3H7A	0.00017	−4.62	−1.72	−2.16
guanine nucleotide binding protein (G protein), beta polypeptide 3	GNB3	0.00016	−4.62	−1.21	−2.80
GS homeobox 2	GSX2	0.00414	−4.59	−1.08	−1.32
CD164 molecule, sialomucin-like	LOC100346891	0.00024	−4.58	−2.13	−3.13
hect domain and RLD 5	HERC5	0.00147	−4.58	1.41	−3.94
non imprinted in Prader-Willi/Angelman syndrome 1	NIPA1	0.00486	−4.58	−1.55	−3.40
numb homolog (Drosophila)	NUMB	<0.00001	−4.58	−1.11	−3.22
G protein-coupled receptor 68	GPR68	0.00006	−4.57	1.92	−2.43
listerin E3 ubiquitin protein ligase 1	LTN1	0.00001	−4.57	−1.31	−3.33
methyltransferase like 10	METTL10	0.00033	−4.56	1.88	−1.47
CD83 molecule	CD83	0.00003	−4.55	1.76	−3.16
Aldosterone synthase Fragment (EC 1.14.15.4)	CYP11B2	0.00001	−4.53	2.52	−3.03
luteinizing hormone beta polypeptide (LHB)	LHB	0.00001	−4.52	1.46	−2.78
Two pore channel 3	TPCN3	0.00008	−4.52	1.47	−3.83
actin binding LIM protein 1	ABLIM1	0.00010	−4.52	−1.61	−2.20
short coiled-coil protein	SCOC	0.00010	−4.52	−1.12	−2.70
anti-Mullerian hormone receptor, type II (AMHR2)	AMHR2	<0.00001	−4.50	1.15	−2.55
family with sequence similarity 13, member C	FAM13C	0.00108	−4.49	−1.91	−3.43
ring finger protein 168, E3 ubiquitin protein ligase	RNF168	<0.00001	−4.48	−1.29	−2.85
MCG49427-like	LOC100343268	<0.00001	−4.46	−1.65	−1.99
Aggrecanase-2	ADAMTS-11^∧, +^	0.00387	−4.44	−1.79	−4.02
NECAP endocytosis associated 2	LOC100357803	0.00001	−4.43	1.37	−3.64
DRAK1 (RDRAK1)	RDRAK1	0.00005	−4.43	−1.64	−3.03
thymopoietin	TMPO	0.00001	−4.42	−1.05	−1.77
serine threonine kinase 39 (STE20/SPS1 homolog, yeast)	STK39	0.00026	−4.41	−2.06	−5.10
Crystallin, zeta	LOC100348912	<0.00001	−4.39	1.45	−1.57
Myeloid/lymphoid or mixed-lineage leukemia 3	MLL3	0.00008	−4.39	−1.32	−2.18
discs, large homolog 3 (Drosophila)	DLG3	0.00008	−4.39	−1.32	−3.99
golgin, RAB6-interacting	GORAB	0.00047	−4.37	−1.57	−2.86
Short coiled-coil protein-like	LOC100354452	0.00008	−4.36	−1.44	−2.77
glycerol kinase 5 (putative)	GK5	0.00019	−4.35	−1.74	−2.42
regulatory factor X, 2 (influences HLA class II expression)	RFX2	0.00003	−4.34	−1.12	−2.22
synaptosomal-associated protein, 25 kDa	SNAP25	0.00087	−4.33	1.08	−7.28
MYB binding protein (P160) 1a	MYBBP1A	0.00246	−4.32	−1.79	−2.30
chromatin modifying protein 2B	CHMP2B	0.00002	−4.31	−1.18	−2.23
protein arginine methyltransferase 3	PRMT3	0.00005	−4.31	−1.57	−1.91
Laminin, alpha 2	LAMA2	<0.00001	−4.30	1.71	−3.04
family with sequence similarity 98, member B	FAM98B	0.00294	−4.29	−2.05	−2.96
ankyrin repeat domain 44	ANKRD44	<0.00001	−4.26	1.36	−2.44
family with sequence similarity 175, member A	FAM175A	0.00028	−4.26	−1.59	−2.22
crumbs homolog 1 (Drosophila)	CRB1	0.00016	−4.23	3.73	−1.72
TBC1 domain family, member 15	TBC1D15	0.00297	−4.23	−1.87	−6.40
Zinc finger, FYVE domain containing 21-like	LOC100354231	0.00016	−4.20	2.03	−3.51
Homeobox protein	HEX	0.00003	−4.19	−1.18	−2.79
TPA-induced transmembrane protein	TTMP	0.00179	−4.18	−1.69	−2.76
RAB3 GTPase activating protein subunit 2 (non-catalytic)	RAB3GAP2	<0.00001	−4.17	−1.25	−2.52
sarcolemmal associated protein-3 (LOC100009199)	SLAP	0.00065	−4.17	−1.68	−2.65
family with sequence similarity 175, member B	FAM175B	0.00057	−4.17	−3.10	−1.67
guanine nucleotide binding protein (G protein), alpha inhibiting activity polypeptide 1	GNAI1	0.00012	−4.15	−1.79	−4.70
Beta amyloid protein precursor	LOC100009546	0.00002	−4.15	1.09	−3.20
golgi integral membrane protein 4	GOLIM4	0.00004	−4.15	−1.69	−2.86
nuclear transport factor 2-like export factor 2	NXT2	0.00009	−4.15	1.04	−2.86
sodium channel, voltage-gated, type VII, alpha	SCN7A	0.00103	−4.14	−2.44	−3.92
transmembrane protein 123	TMEM123	0.00005	−4.13	−1.52	−2.83
protein tyrosine phosphatase domain containing 1	PTPDC1	0.00286	−4.11	1.13	−3.15
malic enzyme 1, NADP(+)-dependent, cytosolic	ME1	0.00007	−4.11	−1.52	−4.16
fibrillarin-like 1	FBLL1	0.00229	−4.11	−1.58	−4.00
Nuclear casein kinase and cyclin-dependent kinase substrate 1-like	LOC100355717	<0.00001	−4.10	1.60	−3.60
Ligand-dependent nuclear receptor-interacting factor 1	LRIF1	0.00001	−4.07	−1.07	−1.82
solute carrier family 44, member 1	SLC44A1	0.00007	−4.07	−1.06	−1.92
Single-stranded DNA binding protein 2-like	LOC100345861	0.00001	−4.07	1.01	−2.98
OMA1 homolog, zinc metallopeptidase (S. cerevisiae)	OMA1	0.00076	−4.06	−1.34	−2.37
glia maturation factor, beta	GMFB	0.00106	−4.05	−1.74	−2.63
transient receptor potential cation channel, subfamily M, member 7	TRPM7	0.00041	−4.05	−1.49	−3.21
translocase of outer mitochondrial membrane 5 homolog (yeast)	TOMM5^∧^	0.00014	−4.05	−5.67	−1.60
fibroblast growth factor 13	FGF13	0.00088	−4.00	−2.20	−4.12
Heterogeneous nuclear ribonucleoprotein A1	LOC100343627	0.00001	−4.00	2.43	−2.17

^∧^ Differentially expressed genes that are common between ‘hypertension only’ and ‘hypercholesterolemia plus sham’ groups (both vs. sham controls).^+^Differentially expressed genes that are common among ‘hypertension only’, ‘hypercholesterolemia plus sham’, and ‘hypertension plus hypercholesterolemia’ groups (all vs. sham controls).

**Figure 7:**
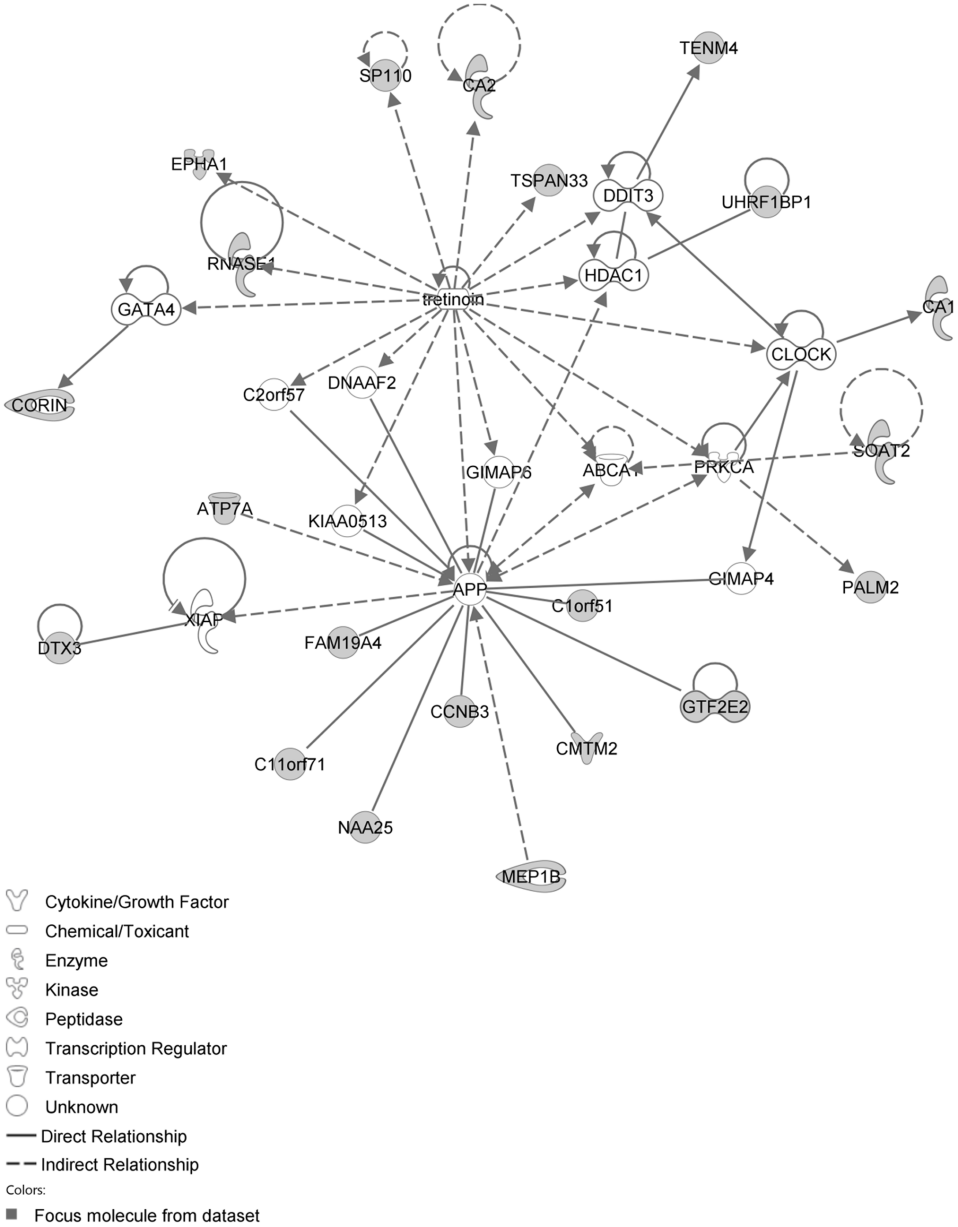
IPA network showing the network with the largest number of up-regulated focus genes in the hypercholesterolemia plus sham group, compared with sham operated controls.

**Figure 8:**
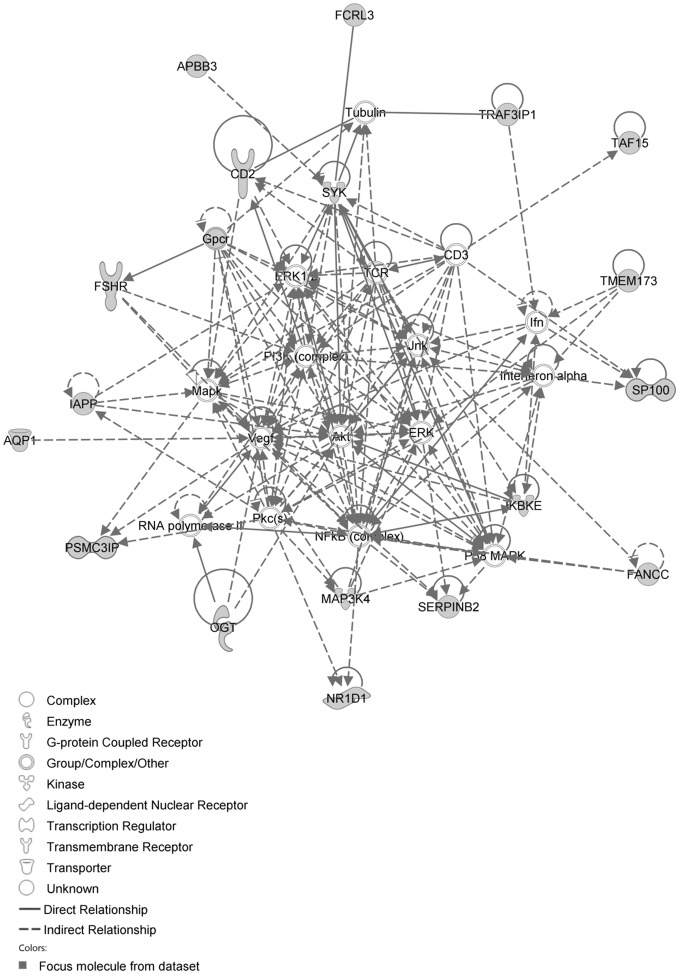
IPA network showing the network with the second largest number of up-regulated focus genes in the hypercholesterolemia plus sham group, compared with sham operated controls.

The IPA network with the ‘largest number of up-regulated focus genes’, contained 21 focus genes with functions in Cell Death and Survival, Lipid Metabolism, Small Molecule Biochemistry. Focus genes in this network were ATP7A, C11orf71, C1orf51, CA1, CA2, CCNB3, CMTM2, CORIN, DTX3, EPHA1, FAM19A4, GTF2E2, MEP1B, NAA25, PALM2, RNASE1, SOAT2, SP110, TENM4, TSPAN33 and UHRF1BP1. They were related to the ‘node molecules’, APP and tretinoin (Figure 7, Table 3). The next network of up-regulated focus genes had 18 focus genes, with functions in Organ Morphology, Reproductive System Development and Function, Developmental Disorder. Focus genes were APBB3, AQP1, CD2, FANCC, FCRL3, FSHR, IAPP, IKBKE, MAP3K4, NR1D1, OGT, PSMC3IP, SERPINB2, SP100, SYK, TAF15, TMEM173 and TRAF3IP1; they were related to P38 MAPK, ERK1/2, NFkB, SERPINB2, Akt, interferon alpha and VEGF (Figure 8, Table 3).

**Figure 9:**
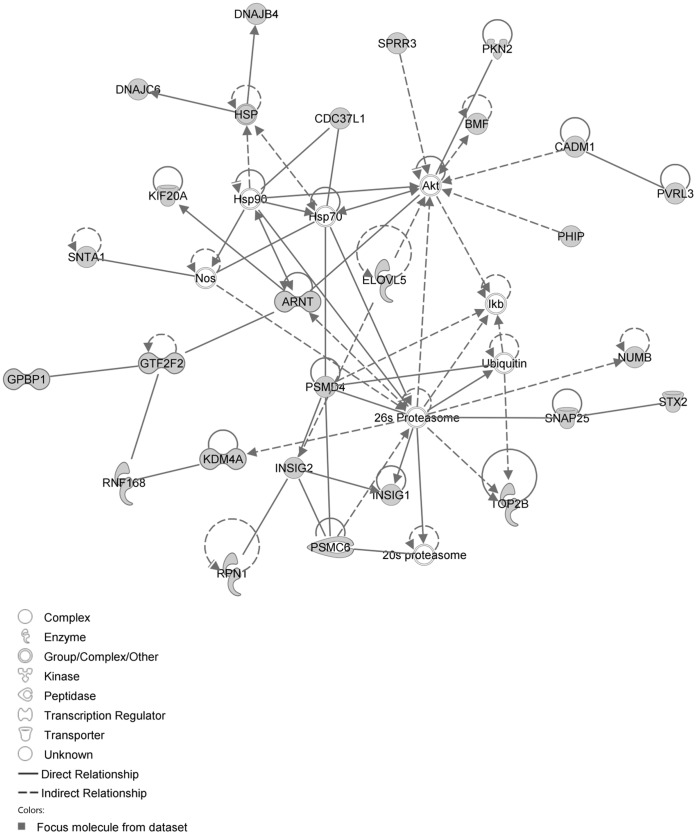
IPA network showing the network with the largest number of down-regulated focus genes in the hypercholesterolemia plus sham group, compared with sham operated controls.

**Figure 10:**
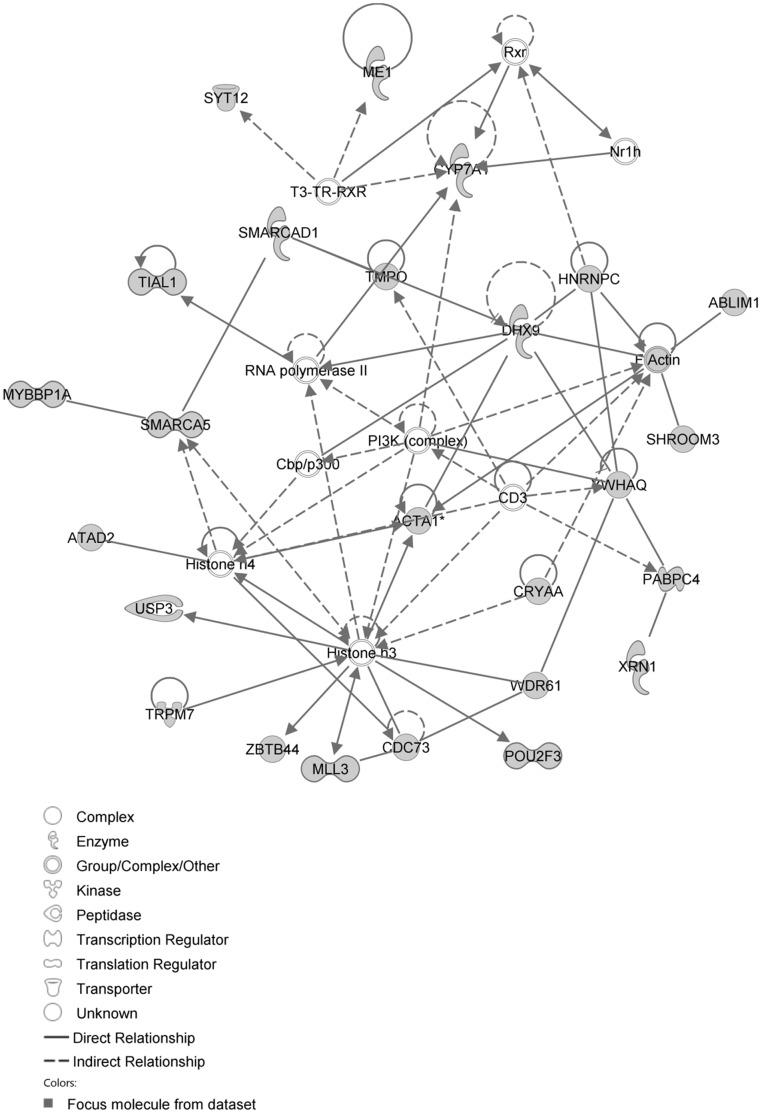
IPA network showing the network with the second largest number of down-regulated focus genes in the hypercholesterolemia plus sham group, compared with sham operated controls.

The network with the largest number of down-regulated focus genes contained 26 focus genes with functions in Tissue Development, Connective Tissue Disorders, and Developmental Disorder. Focus genes in this network were ARNT, BMF, CADM1, CDC37L1, DNAJB4, DNAJC6, ELOVL5, GPBP1, GTF2F2, INSIG1, INSIG2, KDM4A, KIF20A, NUMB, PHIP, PKN2, PSMC6, PSMD4, PVRL3, RNF168, RPN1, SNAP25, SNTA1, SPRR3, STX2 and TOP2B. They were related to Ubiquitin, 26s Proteasome and Akt (Figure 9, Table 4). The next network of down-regulated focus genes had 25 focus genes with functions in Organ Morphology, Visual System Development and Function, Lipid Metabolism. Focus genes were ABLIM1, ACTA1, ATAD2, CDC73, CRYAA, CYP7A1, DHX9, HNRNPC, ME1, MLL3, MYBBP1A, PABPC4, POU2F3, SHROOM3, SMARCA5, SMARCAD1, SYT12, TIAL1, TMPO, TRPM7, USP3, WDR61, XRN1, YWHAQ and ZBTB44; they were related to histone H3 and F Actin (Figure 10, Table 4).

#### 2.3. Microarray analyses of the hypertension plus hypercholesterolemia group

The gene expression profile in the MCA of the hypertension plus hypercholesterolemia group was compared with that of sham controls on a normal diet. After unknown and repeated genes were omitted, 222 up-regulated and 133 down-regulated genes (greater than 4-fold change) were found (Figure 2). Among the highly up-regulated genes in the MCA of the hypertension plus hypercholesterolemia group compared to sham controls were EPHA1, SP110, SLFN14 (Table 5). Among the highly down-regulated genes were FOXN1, TNFRSF11B and GAPDHS (Table 6).

**Table 5:** Up-regulated genes in the MCA of ‘hypertension plus hypercholesterolemia’ rabbits vs. sham controls with greater than 4-fold change.

Description	GeneSymbol	Corrected *p*-value	Hypertension plus hyperchole-sterolemia	Hypertension only	Hyperchole-sterolemia plus sham
			Fold change	Fold change	Fold change
EPH receptor A1	EPHA1	0.00008	20.31	3.03	8.98
SP110 nuclear body protein	SP110	<0.00001	15.47	2.06	8.42
schlafen family member 14	SLFN14	0.00614	13.64	1.33	10.65
Glucose-fructose oxidoreductase domain containing 2-like	LOC100351150^∧, +^	0.00001	12.42	4.01	6.34
Gonadotropin-releasing hormone receptor	GnRHR	0.00008	10.39	3.11	5.47
solute carrier family 9, subfamily A (NHE8, cation proton antiporter 8), member 8	SLC9A8	0.00175	10.32	3.55	6.14
carbonic anhydrase I [	CA1	0.00531	10.07	2.38	10.02
Gap junction alpha-3 protein-like	LOC100357902	0.00689	9.96	2.92	9.62
pleckstrin homology domain containing, family G (with RhoGef domain) member 6	PLEKHG6	0.00011	9.24	3.23	4.47
Uncharacterized protein C11orf71	C11orf71	0.00446	9.22	2.97	8.33
carboxypeptidase A5	CPA5	0.00007	9.11	3.29	4.56
Serine proteinase inhibitor, clade B, member 2 (Predicted)	SERPINB2^∧, +^	0.00005	9.10	5.63	6.99
kinesin family member 13B	KIF13B	0.00002	9.02	2.35	6.02
MRS2 magnesium homeostasis factor homolog (S. cerevisiae)	MRS2	<0.00001	8.85	2.38	3.90
Serine/threonine kinase 23, muscle-specific serine kinase 1 70 (Predicted)	SRPK3	0.00027	8.51	1.35	4.23
4-hydroxyphenylpyruvate dioxygenase-like	HPDL	0.00008	8.45	2.22	4.68
HEAT repeat containing 3	LOC100357872	0.00100	8.34	2.38	3.77
guanylate cyclase 2D, membrane (retina-specific)	GUCY2D^∧, +^	0.00008	8.34	4.64	4.11
membrane-spanning 4-domains, subfamily A, member 2	MS4A2	0.00891	8.29	2.43	3.81
Mast cell-expressed membrane protein 1	MCEMP1	0.00003	8.27	1.74	4.67
follicle stimulating hormone receptor	FSHR	0.00023	8.27	1.24	5.31
spectrin, alpha, erythrocytic 1 (elliptocytosis 2)	SPTA1	0.00375	8.20	2.75	6.03
Bardet-Biedl syndrome 5	LOC100342443	0.00004	8.15	2.76	5.62
Uncharacterized protein C1orf50	C1orf50	0.00006	8.01	5.81	3.80
transmembrane protein 212	TMEM212	0.00039	7.93	3.48	8.84
CKLF-like MARVEL transmembrane domain containing 2	CMTM2	0.00071	7.79	2.38	6.28
Double zinc ribbon and ankyrin repeat-containing protein 1	DZANK1^∧, +^	<0.00001	7.37	4.87	5.04
doublesex and mab-3 related transcription factor 2	DMRT2	0.00002	7.32	1.81	4.28
LY6/PLAUR domain containing 5	LYPD5	0.00004	7.31	2.68	4.70
kinesin family member 21A	KIF21A	0.00003	7.31	2.10	2.93
N(alpha)-acetyltransferase 25, NatB auxiliary subunit	NAA25^∧, +^	0.00099	7.23	1.04	3.68
General transcription factor IIB	LOC100359058	0.00015	7.00	2.64	3.67
relaxin/insulin-like family peptide receptor 2	RXFP2^∧, +^	0.00029	6.96	4.79	5.62
interleukin 20 receptor, alpha	IL20RA	<0.00001	6.96	2.30	3.79
family with sequence similarity 46, member C	FAM46C	0.00016	6.92	1.72	8.51
Fructose-1,6-bisphosphatase 1	FBP1	0.00008	6.82	1.96	2.85
transmembrane protease, serine 11E	TMPRSS11E	<0.00001	6.76	1.26	4.57
IDI1 protein-like	LOC100346274^∧, +^	0.00042	6.67	5.51	4.87
solute carrier family 18 (vesicular monoamine), member 1	SLC18A1	0.00074	6.64	1.42	3.45
IQ motif and ubiquitin domain containing	IQUB	0.00104	6.64	1.90	2.98
family with sequence similarity 71, member C	FAM71C	0.00026	6.63	2.04	3.24
protease, serine, 38	PRSS38	0.00058	6.51	1.95	5.74
signal-induced proliferation-associated 1 like 3	SIPA1L3^∧, +^	0.00001	6.50	4.80	8.45
secretoglobin, family 3A, member 1	SCGB3A1	0.00010	6.42	−1.18	3.56
STAM binding protein-like 1	STAMBPL1	0.00286	6.39	1.40	2.37
Fanconi anemia, complementation group C	FANCC^∧, +^	<0.00001	6.39	6.10	4.25
tripartite motif-containing 67	TRIM67	0.00175	6.39	2.15	3.25
Serum response factor binding protein 1-like	LOC100349313	0.00139	6.34	2.18	3.53
Interstitial collagenase Precursor (EC 3.4.24.7)(Matrix metalloproteinase-1)(MMP-1)	MMP1	0.00149	6.33	8.12	2.20
G protein-coupled receptor 119	GPR119	0.00058	6.30	2.41	2.00
RNA binding motif protein 6	RBM6	0.00003	6.15	1.73	4.85
2′,3′-cyclic nucleotide 3′ phosphodiesterase	CNP	<0.00001	6.14	1.42	3.45
nuclear receptor subfamily 1, group D, member 1	NR1D1	0.00562	6.11	2.35	4.98
cyclin B3	CCNB3	0.00012	6.05	1.68	4.39
steroidogenic acute regulatory protein	STAR	0.00007	6.05	3.36	3.31
CD4 molecule (CD4)	CD4	0.00004	6.02	1.17	2.77
DOMON domain-containing protein FRRS1L	FRRS1L	0.00003	5.99	1.37	3.57
Tubulin tyrosine ligase-like family, member 5	LOC100359240	0.00019	5.99	1.63	2.92
family with sequence similarity 19 (chemokine (C-C motif)-like), member A4	FAM19A4	0.00024	5.97	1.65	4.17
sterol O-acyltransferase 2	SOAT2	0.00003	5.94	3.01	7.18
aminolevulinate, delta-, synthase 2	ALAS2	0.00072	5.93	1.97	5.86
thymidine phosphorylase	TYMP	0.00005	5.87	1.17	3.87
dynein heavy chain domain 1	DNHD1	0.00134	5.87	2.10	3.49
insulin-like 5 (INSL5),	INSL5	0.00018	5.85	1.39	3.03
meprin A, beta	MEP1B^∧, +^	0.00291	5.85	5.37	5.12
arylformamidase	AFMID	0.00100	5.81	1.65	4.03
Islet amyloid polypeptide	IAPP	0.00004	5.78	1.40	4.17
Transcription elongation factor A (SII)-like 2-like	LOC100340955	0.00015	5.75	3.13	3.22
G protein-coupled receptor 52	GPR52	0.00010	5.75	2.26	4.40
solute carrier family 5 (sodium/glucose cotransporter), member 9 (SLC5A9)	SLC5A9	0.00002	5.73	3.82	3.28
Kruppel-like factor 13	KLF13	0.00007	5.69	1.11	3.32
Granulate-macrophage stimulating factor (Predicted)	GMCSF	0.00004	5.68	2.22	3.39
listerin E3 ubiquitin protein ligase 1	LTN1	0.00009	5.66	3.05	2.75
zinc finger, DHHC-type containing 23	ZDHHC23	0.00001	5.64	7.18	3.22
carboxylesterase 8 (putative)	CES4A	0.00023	5.63	1.47	3.83
C-type lectin domain family 1, member B	CLEC1B	0.00584	5.60	1.50	3.65
TNF receptor-associated factor 3 interacting protein 1	TRAF3IP1	0.00043	5.56	1.29	4.80
solute carrier family 15, member 5	SLC15A5	0.00075	5.55	1.80	2.70
glucosidase, beta, acid 3 (cytosolic)	GBA3	0.00012	5.55	2.27	2.77
tetraspanin 33	TSPAN33	0.00078	5.53	3.97	6.37
Proline-rich protein 3	PRR3	0.00129	5.50	1.24	3.46
chemokine (C-C motif) ligand 19	CCL19	0.00055	5.50	2.16	2.39
NADH dehydrogenase (ubiquinone) Fe-S protein 1, 75kDa	LOC100341941	0.00022	5.46	2.69	4.34
AchR epsilon subunit Fragment	CHRNE	0.00010	5.43	1.33	3.22
Transmembrane protein C5orf28	C5orf28	0.00319	5.38	2.31	2.49
dehydrogenase/reductase (SDR family) member 9	DHRS9	0.00168	5.37	3.41	3.41
FCH and double SH3 domains 1	FCHSD1	0.00015	5.37	2.85	3.24
leucine-rich repeat-containing G protein-coupled receptor 5	LGR5 *	0.00019	5.36	3.32	4.14
Uncharacterized protein C7orf72	C7orf72	0.00114	5.35	-1.29	2.68
family with sequence similarity 71, member F2	FAM71F2	0.00643	5.34	1.21	2.13
otopetrin 2	OTOP2	0.00006	5.31	3.74	3.23
ELOVL fatty acid elongase 3	ELOVL3	0.00012	5.30	1.83	3.05
solute carrier family 7 (anionic amino acid transporter), member 13	SLC7A13	0.00134	5.30	1.82	2.81
Uncharacterized protein C2orf71	C2orf71	0.00177	5.30	1.36	3.60
ankyrin and armadillo repeat containing	ANKAR^∧, +^	0.00006	5.23	7.05	7.99
solute carrier family 14 (urea transporter), member 1 (Kidd blood group)	SLC14A1	0.00027	5.21	1.61	3.38
zinc finger, CW type with PWWP domain 2	ZCWPW2	0.00011	5.21	2.31	5.29
Fc receptor-like A	FCRLA	0.00083	5.14	1.94	6.76
RAS guanyl releasing protein 4	RASGRP4	0.00058	5.11	2.61	3.68
family with sequence similarity 167, member A	FAM167A	0.00017	5.11	14.04	2.44
ATPase, aminophospholipid transporter, class I, type 8B, member 2	ATP8B2	0.00360	5.07	3.16	3.14
Cytochrome P450, family 4, subfamily A, polypeptide 5	CYP4A5	0.00061	5.05	1.03	3.12
ATPase, H+/K+ exchanging, alpha polypeptide (ATP4A)	ATP4A	<0.00001	5.02	1.47	3.53
CD2 molecule	CD2	0.00064	4.99	1.35	3.15
3-oxo-5-alpha-steroid 4-dehydrogenase 2-like	LOC100343882	0.00001	4.99	2.48	2.64
ankyrin repeat and SOCS box-containing 4	ASB4^∧^	<0.00001	4.97	6.36	3.70
phosphatidylcholine transfer protein	PCTP	0.00001	4.96	2.13	3.26
solute carrier family 39 (zinc transporter), member 12	SLC39A12	0.00006	4.96	4.23	2.48
transmembrane emp24 protein transport domain containing 6	TMED6	0.00040	4.96	2.10	3.19
actin-related protein T1	ACTRT1	0.00005	4.94	2.95	3.11
Wings apart-like homolog	LOC100348678	0.00041	4.94	2.27	2.92
Fructose-1,6-bisphosphatase 1	FBP1	0.00050	4.88	1.51	2.15
spleen tyrosine kinase	SYK	0.00072	4.88	1.52	4.13
tripartite motif-containing 65	TRIM65	0.00079	4.87	2.63	4.05
neuron navigator 2	NAV2	0.00080	4.87	1.80	2.69
potassium channel tetramerisation domain containing 19	KCTD19	0.00123	4.87	1.42	3.10
spermatogenesis associated, serine-rich 1	SPATS1	0.00201	4.83	2.77	2.57
solute carrier family 5 (sodium/glucose cotransporter), member 1	SLC5A1	0.00001	4.80	2.26	2.95
tripartite motif-containing 35	TRIM35	0.00084	4.80	1.38	3.16
cytochrome P450, family 26, subfamily C, polypeptide 1	CYP26C1	0.00224	4.79	-1.58	2.30
Polymeric immunoglobulin receptor	PIGR	0.00025	4.75	1.40	2.82
Serine incorporator 1	LOC100357075	0.00015	4.74	2.50	5.37
Ras and Rab interactor-like	RINL	0.00135	4.71	1.67	3.40
ubiquitin associated and SH3 domain containing B	UBASH3B	0.00803	4.70	2.95	3.29
UDP-N-acetyl-alpha-D-galactosamine:polypeptide N-acetylgalactosaminyltransferase 13 (GalNAc-T13)	GALNT13	<0.00001	4.70	2.52	1.93
transmembrane protein 173	TMEM173	0.00141	4.70	2.88	5.36
UHRF1 binding protein 1	UHRF1BP1	0.00074	4.69	2.63	5.70
PRP18 pre-mRNA processing factor 18 homolog (S. cerevisiae)	PRPF18	0.00144	4.68	1.10	2.28
Carbonic anhydrase 2 (EC 4.2.1.1)(Carbonic anhydrase II)(CA-II)(Carbonate dehydratase II)	CA2	0.00964	4.68	1.67	4.51
spinster homolog 3 (Drosophila)	SPNS3	0.00049	4.68	2.40	3.63
galactosidase, beta 1-like 3	GLB1L3	0.00003	4.67	1.24	3.03
iroquois homeobox 2	IRX2	0.00052	4.66	-1.04	3.40
collagen, type XVII, alpha 1	COL17A1	0.00028	4.65	1.07	2.60
patched homolog 2 (Drosophila)	PTCH2	0.00005	4.65	2.55	3.84
TAF15 RNA polymerase II, TATA box binding protein (TBP)-associated factor, 68 kDa	TAF15^∧, +^	0.00004	4.65	7.45	4.71
BCL2-like 10 (apoptosis facilitator)	BCL2L10	0.00018	4.64	1.67	5.34
Mix1 homeobox-like 1 (Xenopus laevis)	MIXL1	0.00093	4.64	5.80	3.54
Fibroblast growth factor binding protein 1-like	LOC100353835^∧, +^	0.00122	4.63	5.01	4.01
corin, serine peptidase	CORIN	0.00006	4.63	3.59	4.93
V-set and immunoglobulin domain containing 2	VSIG2	0.00168	4.60	3.51	5.53
Pregnancy-zone protein	PZP	0.00099	4.60	1.20	4.11
DEAQ box RNA-dependent ATPase 1	DQX1	0.00520	4.60	1.85	2.94
Potassium channel, subfamily K, member 1	KCNK1	0.00217	4.58	3.22	1.68
purinergic receptor P2Y, G-protein coupled, 10	P2RY10	0.00197	4.57	1.99	2.24
coiled-coil domain containing 54	CCDC54	0.00196	4.57	3.11	4.04
somatostatin receptor 2	SSTR2	0.00077	4.56	2.78	2.44
deltex homolog 3 (Drosophila)	DTX3	0.00013	4.56	3.47	4.92
zinc finger, DHHC-type containing 19	ZDHHC19	0.00241	4.55	1.65	3.32
ret proto-oncogene	RET	0.00036	4.55	1.88	2.71
MORN repeat containing 4	MORN4	<0.00001	4.53	1.45	4.14
Oxytocin receptor	OTXR	0.00517	4.52	1.10	2.58
serine peptidase inhibitor, Kazal type 1	SPINK1	0.00055	4.50	1.60	2.53
membrane protein, palmitoylated 2 (MAGUK p55 subfamily member 2)	MPP2	0.00409	4.50	2.36	2.89
solute carrier family 13 (sodium/sulfate symporters), member 1	SLC13A1	0.00325	4.49	1.59	1.58
follistatin-like 5	FSTL5	0.00071	4.49	3.17	2.31
Mitochondrial ribosomal protein S15-like	LOC100345026	0.00064	4.49	2.82	2.65
TERF1 (TRF1)-interacting nuclear factor 2	TINF2	0.00325	4.49	2.19	3.29
keratin associated protein 19-4	KRTAP19-4	0.00044	4.48	1.36	2.78
XK, Kell blood group complex subunit-related, X-linked	XKRX	0.00051	4.47	2.36	2.54
Chloride intracellular channel 2 (Predicted)	CLIC2	0.00279	4.46	1.60	5.43
FK506 binding protein 1B, 12.6 kDa	FKBP1B	0.00274	4.46	−1.46	2.32
phospholipase C, zeta 1	PLCZ1	0.00002	4.45	1.01	2.92
CD244 molecule, natural killer cell receptor 2B4	CD244	0.00003	4.44	−1.55	2.90
carboxyl ester lipase (bile salt-stimulated lipase) (CEL)	CEL	0.00016	4.44	1.98	2.68
ADAM metallopeptidase with thrombospondin type 1 motif, 18	ADAMTS18	0.00213	4.43	1.94	2.90
UDP-glucuronosyltransferase	UGT2C1	0.00035	4.42	3.99	2.75
fms-related tyrosine kinase 3	FLT3	0.00270	4.41	1.80	3.63
mitogen-activated protein kinase binding protein 1	MAPKBP1	0.00003	4.41	3.08	2.68
Ammonium transporter Rh type C (Rhesus blood group family type C glycoprotein)(Rh family type C glycoprotein)(Rh type C glycoprotein)	RHCG	0.00032	4.41	2.20	3.29
Kelch-like 1 (Drosophila)	KLHL1	0.00003	4.35	−1.02	2.80
leucine rich repeat transmembrane neuronal 4	LRRTM4	0.00002	4.34	3.58	2.67
proteoglycan 3	PRG3	0.00254	4.33	1.60	3.79
Ligand of numb-protein X 1-like	LOC100343259	0.00568	4.33	1.19	2.97
chymase like protein	LOC100008644	0.00060	4.32	−1.31	2.21
sex comb on midleg-like 4 (Drosophila)	SCML4	0.00854	4.32	1.40	2.94
BMS1-like, ribosome assembly protein	LOC100339194	0.00017	4.31	2.21	3.44
Uncharacterized protein C2orf61	C2orf61	0.00893	4.31	3.08	3.04
REC8 homolog (yeast)	REC8	<0.00001	4.31	1.93	5.52
Protein FAM55A	FAM55A	0.00326	4.30	1.16	1.70
cyclin-dependent kinase 15	CDK15	0.00129	4.28	1.93	3.69
transmembrane and coiled-coil domains 4	TMCO4	0.00007	4.28	2.43	5.42
Rac GTPase activating protein 1 pseudogene	RACGAP1P	0.00023	4.27	1.43	2.33
family with sequence similarity 170, member A	FAM170A	0.00008	4.23	2.43	2.99
Mitogen-activated protein kinase kinase kinase 4	MAP3K4	0.00788	4.22	2.40	3.16
ATPase, H+ transporting, lysosomal 70 kDa, V1 subunit A	ATP6V1A	0.00397	4.19	1.38	1.78
solute carrier family 9, subfamily A (NHE5, cation proton antiporter 5), member 5	SLC9A5	0.00057	4.19	2.33	5.36
2,3-bisphosphoglycerate mutase (BPGM)	BPGM	0.00098	4.18	1.21	4.00
FRY-like	FRYL	0.00291	4.18	1.46	2.56
clusterin-like 1 (retinal)	CLUL1	0.00072	4.17	2.09	2.68
Superiorcervical ganglia, neural specific 10	LOC100344464	0.00228	4.14	1.78	1.59
Histamine receptor H1	HRH1	0.00001	4.13	1.92	2.38
Cingulin (Predicted)	CGN	0.00236	4.12	1.43	3.44
nuclear factor of kappa light polypeptide gene enhancer in B-cells inhibitor, delta	NFKBID	0.00103	4.12	1.13	3.05
ribonuclease, RNase A family, 1 (pancreatic)]	RNASE1^∧, +^	0.00043	4.11	2.83	3.92
MLX interacting protein	MLXIP	0.00069	4.09	3.02	5.36
Nuclear prelamin A recognition factor	LOC100344024	0.00002	4.09	2.47	2.46
actin, beta-like 2	ACTBL2	0.00023	4.09	1.32	2.55
ghrelin receptor (LOC100101582)	LOC100101582	0.00062	4.08	1.40	2.17
olfactomedin 2	OLFM2	0.00385	4.07	3.66	2.50
cytochrome c oxidase assembly homolog 10 (yeast)	COX10	0.00160	4.07	2.06	3.97
ribonuclease L (2′,5′-oligoisoadenylate synthetase-dependent)	RNASEL	0.00028	4.06	−1.16	1.98
keratin associated protein 11-1	KRTAP11-1	0.00013	4.04	2.16	2.57
family with sequence similarity 53, member C	FAM53C	<0.00001	4.03	9.87	2.48
DnaJ (Hsp40) homolog, subfamily C, member 5 beta	DNAJC5B	0.00016	4.03	−1.01	2.35
receptor (chemosensory) transporter protein 1	RTP1	0.00012	4.03	−1.25	1.76
protocadherin 8	PCDH8	0.00005	4.02	3.20	3.09
Ubiquitin specific protease 39	LOC100344046	0.00343	4.02	2.49	2.75
corticotropin releasing hormone binding protein	CRHBP	0.00005	4.02	6.38	2.23
proline/serine-rich coiled-coil 1	PSRC1	0.00571	4.01	1.63	2.68
cadherin 17, LI cadherin (liver-intestine)	CDH17	0.00459	4.00	1.59	2.72

^∧^ Differentially expressed genes that are common between ‘hypertension only’ and ‘hypercholesterolemia plus sham’ groups (both vs. sham controls).^+^Differentially expressed genes that are common among ‘hypertension only’, ‘hypercholesterolemia plus sham’, and ‘hypertension plus hypercholesterolemia’ groups (all vs. sham controls).

**Table 6:** Down-regulated genes in the MCA of ‘hypertension plus hypercholesterolemia’ rabbits vs. sham controls with greater than 4-fold change.

Description	GeneSymbol	Corrected *p*-value	Hypertension plus hyperchole-sterolemia	Hypertension only	Hyperchole-sterolemia plus sham
			Fold Change	Fold Change	Fold Change
forkhead box N1	FOXN1^∧, +^	0.00048	−45.12	−26.20	−56.89
Tumor necrosis factor receptor superfamily, member 11b	TNFRSF11B	<0.00001	−33.58	1.69	−55.91
glyceraldehyde-3-phosphate dehydrogenase, spermatogenic	GAPDHS	<0.00001	−32.91	1.01	−33.66
Chaperonin containing TCP1, subunit 2 (beta)	CCT2	<0.00001	−30.96	−1.91	−35.56
Tweety homolog 1 (Drosophila)	TTYH1	0.00108	−27.02	−5.16	−12.82
family with sequence similarity 184, member B	FAM184B	<0.00001	−25.72	−1.32	−68.05
3′ (2′), 5′-bisphosphate nucleotidase 1	BPNT1	<0.00001	−25.01	1.05	−52.27
ubiquinol-cytochrome c reductase, Rieske iron-sulfur polypeptide 1 pseudogene 1	UQCRFS1P1	<0.00001	−23.74	1.08	−40.99
Progesterone receptor membrane component 1	LOC100357097	<0.00001	−21.91	1.04	−38.66
microtubule associated tumor suppressor 1	MTUS1	<0.00001	−20.40	−1.88	−41.71
transmembrane protein 38B	TMEM38B	<0.00001	−18.80	1.05	−31.53
beta tropomyosin (LOC100125984)	LOC100125984	<0.00001	−17.13	−2.30	−97.92
ORM1-like 3 (S. cerevisiae)	ORMDL3	<0.00001	−16.75	−1.24	−17.49
DCN1, defective in cullin neddylation 1, domain containing 4 (S. cerevisiae)	DCUN1D4	<0.00001	−15.90	−1.65	−18.27
Lysosomal-associated membrane protein 2-like	LOC100350946	<0.00001	−15.88	−1.78	−26.85
v-Ki-ras2 Kirsten rat sarcoma viral oncogene homolog	KRAS	<0.00001	−15.47	−1.18	−22.11
androgen-induced 1	AIG1	0.00002	−14.63	−3.30	−35.48
Eukaryotic translation initiation factor 2 subunit 1	EIF2S1	0.00001	−14.40	−2.04	−16.08
hematopoietic prostaglandin D synthase	HPGDS^∧, +^	0.00043	−13.63	−12.38	−21.44
Nucleolar protein 11	NOL11	0.00032	−13.01	2.09	−11.98
zinc finger protein 642	ZFP69	0.00058	−12.02	−1.58	−20.24
mitochondrial ribosomal protein L1	MRPL1	<0.00001	−12.00	−1.06	−15.71
WW domain containing adaptor with coiled-coil	WAC	0.00001	−11.91	−3.12	−13.20
Solute carrier family 2, facilitated glucose transporter member 3	SLC2A3	0.00019	−11.60	−1.81	−10.95
crystallin, alpha A (CRYAA)	CRYAA	<0.00001	−10.81	−1.50	−60.93
Lupus La protein	SSB	<0.00001	−10.68	−1.63	−40.45
somatostatin	SST	0.00169	−10.49	1.22	−4.19
Manganese superoxide dismutase	SOD-2	<0.00001	−9.93	−1.46	−51.78
mitochondrial ribosomal protein L15	MRPL15^∧, +^	<0.00001	−9.57	−6.97	−23.01
Proteasome (prosome, macropain) 26s subunit, non-ATPase, 4	PSMD4^∧, +^	<0.00001	−9.55	−1.65	−12.56
IQ motif containing GTPase activating protein 3	IQGAP3	0.00007	−9.37	1.25	−10.54
cellular disintegrin ADAM 6d (ADAM6)	ADAM6	0.00009	−9.13	−1.03	−3.07
mRNA for prolactin receptor	PRLR	0.00001	−8.55	1.23	−1.32
zinc finger protein 330	ZNF330	0.00012	−8.54	−1.48	−7.41
ELAV (embryonic lethal, abnormal vision, Drosophila)-like 4 (Hu antigen D)	ELAVL4	0.00033	−8.49	1.79	−3.26
Intraflagellar transport 140-like	LOC100355837	<0.00001	−8.45	3.58	−5.61
SWI/SNF related, matrix associated, actin dependent regulator of chromatin, subfamily a, member 5	SMARCA5	<0.00001	−8.08	1.08	−19.50
NADH dehydrogenase-like	LOC100347823	<0.00001	−7.93	2.02	−7.55
zinc finger RNA binding protein	ZFR	<0.00001	−7.90	−1.47	−64.53
Cytoplasmic beta-actin	LOC100009506	<0.00001	−7.80	−1.19	−20.51
stathmin-like 2	STMN2	0.00129	−7.60	1.78	−4.10
SRY (sex determining region Y)-box 2-like	LOC100341629	0.00001	−7.56	5.05	1.43
synaptosomal-associated protein, 25kDa	SNAP25	0.00087	−7.28	1.08	−4.33
general transcription factor IIE, polypeptide 2, beta 34 kDa	GTF2E2	<0.00001	−7.27	1.06	−7.79
Solute carrier family 9, subfamily A (NHE2, cation proton antiporter 2), member 2	SLC9A2	0.00007	−7.21	−2.62	−36.54
olfactomedin 3	OLFM3	0.00005	−6.99	−1.25	−3.51
pyruvate dehydrogenase kinase, isozyme 4	PDK4	0.00711	−6.98	−2.51	−3.47
myelin-associated oligodendrocyte basic protein	MOBP	0.00776	−6.94	−1.01	−3.86
synaptotagmin XII	SYT12	0.00001	−6.91	1.14	−5.36
Titin	TTN	0.00001	−6.90	−2.26	−13.60
BPI fold containing family B, member 6	BPIFB6	0.00001	−6.83	−1.79	−5.92
guanylate cyclase 1, soluble, beta 3	GUCY1B3	0.00010	−6.79	−2.52	−11.97
prefoldin subunit 5	PFDN5	<0.00001	−6.79	−1.78	−84.52
proteolipid protein 1 (PLP1)	PLP1	0.00339	−6.50	−1.79	−6.44
sodium channel, voltage-gated, type VII, alpha	SCN7A	0.00021	−6.43	−1.77	−12.29
TBC1 domain family, member 15	TBC1D15	0.00297	−6.40	−1.87	−4.23
DnaJ (Hsp40) homolog, subfamily C, member 6	DNAJC6	0.00001	−6.36	−1.48	−9.39
zinc finger protein 326	ZNF326	<0.00001	−6.29	1.09	−18.36
neurocalcin delta	NCALD	0.00037	−6.13	−1.93	−9.22
cullin 3	CUL3	<0.00001	−6.10	1.05	−73.37
caudal type homeobox 1	CDX1	0.00001	−6.08	−1.18	−10.42
ring finger protein 222	RNF222	0.00001	−6.02	−1.78	−8.36
syntrophin, alpha 1	SNTA1	<0.00001	−5.98	−1.04	−9.97
Creatine kinase, muscle	CKM	0.00005	−5.96	2.01	−1.29
Sin3A-associated protein, 30 kDa	SAP30	<0.00001	−5.90	1.30	−43.67
signal recognition particle 54 kDa	SRP54	<0.00001	−5.72	1.62	−16.53
OTU domain containing 6A	OTUD6A	0.00011	−5.70	−3.59	−28.99
complement component 1, q subcomponent-like 3	C1QL3	0.00232	−5.67	−2.89	−9.59
TRAF family member-associated NFKB activator	TANK	0.00001	−5.66	−1.28	−20.39
interferon induced with helicase C domain 1	IFIH1	0.00001	−5.60	1.21	−10.96
Paired box protein Pax-4 (Predicted)	PAX4	0.00001	−5.45	2.04	−2.45
IQ motif containing E	IQCE	<0.00001	−5.18	1.75	−7.52
kinesin family member 20A	KIF20A	0.00003	−5.16	−2.21	−6.68
nucleolar protein 10	NOL10	<0.00001	−5.15	1.08	−14.02
Aryl hydrocarbon receptor nuclear translocator (ARNT protein)(Dioxin receptor, nuclear translocator)(Hypoxia-inducible factor 1 beta)(HIF-1 beta)	ARNT	<0.00001	−5.12	1.04	−13.43
Ribosomal protein L7-like	LOC100339887	0.00001	−5.10	−1.23	−6.70
serine threonine kinase 39	STK39	0.00026	−5.10	−2.06	−4.41
SPHK1 interactor, AKAP domain containing	SPHKAP	0.00629	−4.99	−2.87	−3.70
NADP-dependent oxidoreductase domain-containing protein 1 EC = 1.-.-.-	NOXRED1	0.00004	−4.98	−1.28	−11.39
neurogranin (protein kinase C substrate, RC3)	NRGN	0.00291	−4.98	−1.08	−2.47
Acidic ribosomal phosphoprotein P0	36B4	0.00003	−4.94	1.43	−6.27
ring finger protein 38	RNF38	0.00023	−4.93	−3.33	−9.14
TIA1 cytotoxic granule-associated RNA binding protein-like 1	TIAL1	0.00001	−4.88	2.34	−13.80
GDNF family receptor alpha 4	GFRA4	0.00025	−4.88	1.29	−2.93
inositol polyphosphate phosphatase-like 1	INPPL1	0.00005	−4.87	−1.14	−5.96
POU class 2 homeobox 3	POU2F3	<0.00001	−4.78	1.89	−7.48
DnaJ (Hsp40) homolog, subfamily B, member 4	DNAJB4	<0.00001	−4.76	−1.86	−9.11
sulfotransferase family 4A, member 1 (SULT4A1)	SULT4A1	0.00285	−4.75	−1.75	−3.88
poly(rC) binding protein 3	PCBP3	<0.00001	−4.72	1.19	−8.33
insulin induced gene 2	INSIG2	<0.00001	−4.71	−3.78	−21.07
guanine nucleotide binding protein (G protein), alpha inhibiting activity polypeptide 1	GNAI1	0.00012	−4.70	−1.79	−4.15
KIAA1549	KIAA1549	0.00019	−4.67	−1.05	−2.55
myosin, light chain 6B, alkali, smooth muscle and non-muscle	MYL6B	0.00002	−4.66	−1.58	−7.23
exostoses (multiple)−like 3	EXTL3	0.00001	−4.65	−4.65	−3.53
Heterogeneous nuclear ribonucleoprotein C (hnRNP C)	HNRNPC	<0.00001	−4.61	−3.29	−35.05
shroom family member 3	SHROOM3	<0.00001	−4.60	−3.82	−7.95
chaperonin containing TCP1, subunit 2 (beta) (CCT2)	CCT2	<0.00001	−4.57	−1.23	−28.67
enabled homolog (Drosophila)	ENAH	0.00002	−4.57	−1.23	−16.36
Bcl2 modifying factor	BMF	<0.00001	−4.55	−1.04	−9.18
ribophorin I	RPN1	0.00002	−4.51	−1.30	−15.81
zinc finger CCCH-type containing 7B	ZC3H7B	0.00176	−4.48	−3.56	−3.09
inhibin, beta E	INHBE	0.00005	−4.42	1.58	−1.73
sialic acid binding Ig-like lectin 14	SIGLEC14	<0.00001	−4.41	1.14	−6.80
gap junction protein, delta 3, 31.9kDa	GJD3	<0.00001	−4.40	1.35	−7.33
interferon-related developmental regulator 1	IFRD1	<0.00001	−4.37	1.33	−51.14
TPA-induced transmembrane protein	TTMP	0.00014	−4.35	−1.23	−10.35
POU class 3 homeobox 4	POU3F4	0.00030	−4.33	1.01	−3.55
microtubule associated monoxygenase, calponin and LIM domain containing 1	MICAL1	0.00010	−4.30	−1.87	−4.81
Ribosomal protein L36a-like	LOC100358069	0.00001	−4.28	−1.04	−4.82
cysteine-rich protein 3	CRIP3	<0.00001	−4.26	1.45	−10.99
transmembrane protein 109 (TMEM109)	TMEM109	<0.00001	−4.26	−1.07	−39.14
Phospholamban	PLN	0.00002	−4.25	−2.00	−7.94
ISL LIM homeobox 2	ISL2	0.00007	−4.24	−1.36	−5.75
protein phosphatase 1, regulatory (inhibitor) subunit 8	PPP1R8	<0.00001	−4.24	1.09	−7.27
dehydrogenase/reductase (SDR family) member 13	DHRS13	<0.00001	−4.23	1.02	−9.82
sarcolemmal associated protein-3 (LOC100009199)	SLAP	<0.00001	−4.19	−1.47	−9.38
Ribosomal protein S7-like	LOC100345715	0.00012	−4.17	−1.91	−4.96
Eukaryotic translation initiation factor 1	LOC100345195	0.00004	−4.16	−1.06	−5.61
sema domain, immunoglobulin domain (Ig), transmembrane domain (TM) and short cytoplasmic domain, (semaphorin) 4D	SEMA4D	<0.00001	−4.16	1.19	−3.24
malic enzyme 1, NADP(+)-dependent, cytosolic	ME1	0.00007	−4.16	−1.52	−4.11
Poly(A) binding protein, cytoplasmic 4 (inducible form)	PABPC4	0.00009	−4.16	1.10	−5.67
WD repeat domain 61	WDR61	<0.00001	−4.14	1.14	−30.33
guanine nucleotide binding protein (G protein), gamma 3	GNG3	<0.00001	−4.14	1.07	−5.64
CD302 molecule	CD302	<0.00001	−4.14	−1.14	−17.51
fibroblast growth factor 13	FGF13	0.00088	−4.12	−2.20	−4.00
pancreatic polypeptide receptor 1 (PPYR1)	PPYR1	0.00003	−4.12	−1.10	−7.22
membrane-associated ring finger (C3HC4) 6, E3 ubiquitin protein ligase	MARCH6	0.00091	−4.11	−3.08	−3.82
ADP-ribosylation factor 4-like	LOC100341862	0.00015	−4.11	−1.20	−2.92
bone morphogenetic protein 7	BMP7	0.00001	−4.08	−1.21	−11.02
potassium channel, subfamily K, member 18	KCNK18	0.00022	−4.05	1.14	−2.91
Aggrecanase-2	ADAMTS-11^∧, +^	0.00387	−4.02	−1.79	−4.44

^∧^ Differentially expressed genes that are common between ‘hypertension only’ and ‘hypercholesterolemia plus sham’ groups (both vs. sham controls).^+^Differentially expressed genes that are common among ‘hypertension only’, ‘hypercholesterolemia plus sham’, and ‘hypertension plus hypercholesterolemia’ groups (all vs. sham controls).

**Figure 11:**
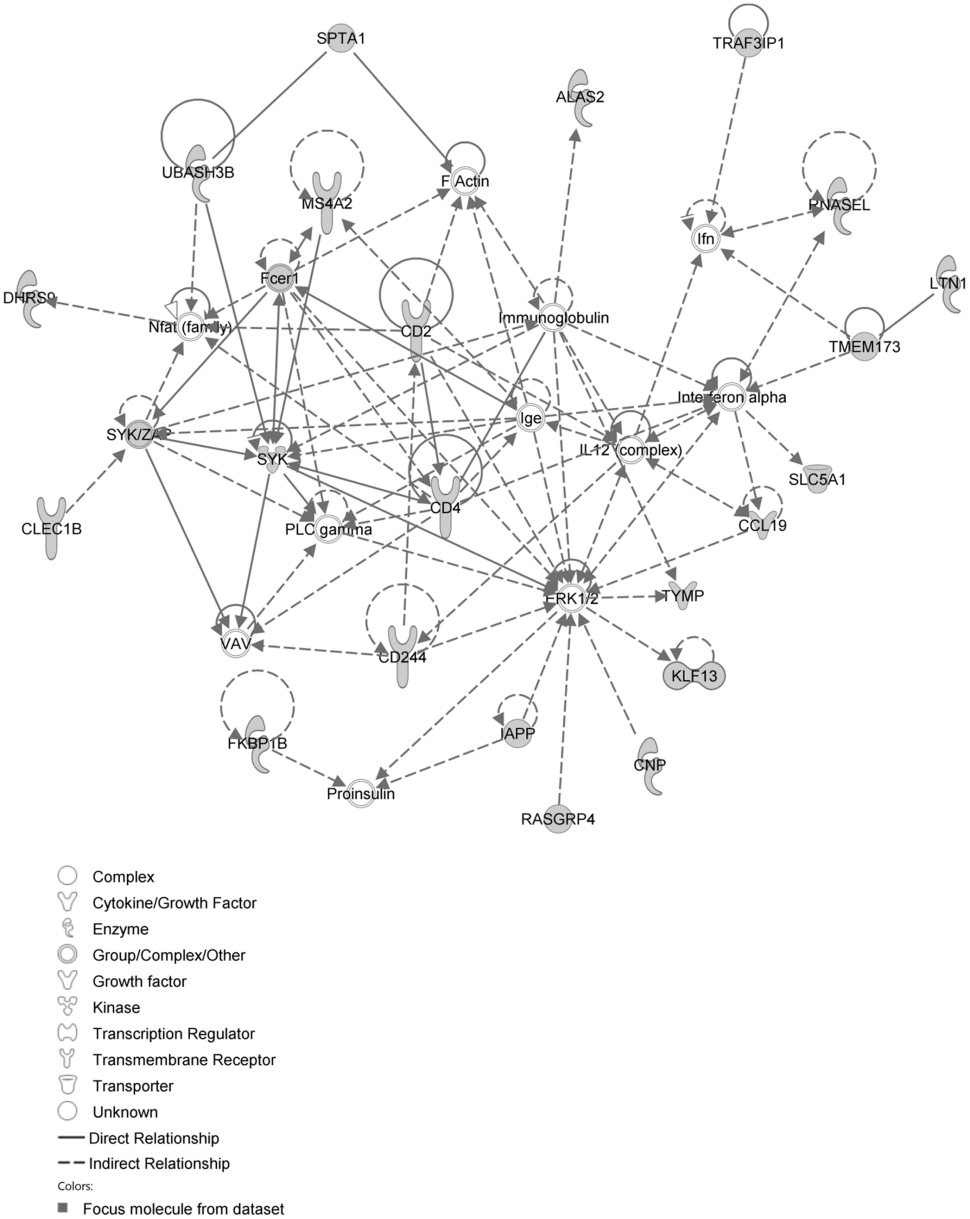
IPA network showing the network with the largest number of up-regulated focus genes in the hypertension plus hypercholesterolemia group, compared with sham operated controls.

**Figure 12:**
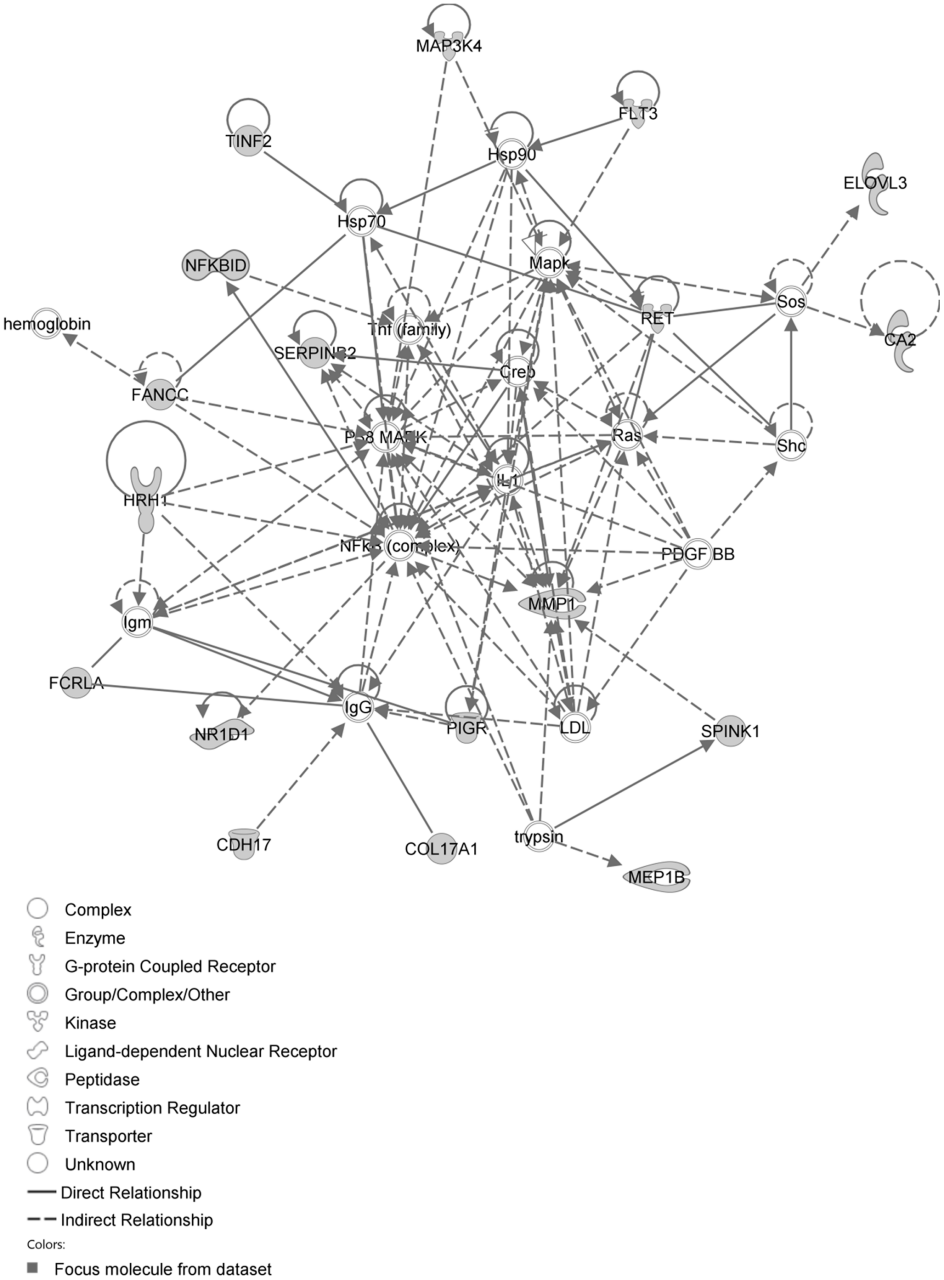
IPA network showing the network with the second largest number of up-regulated focus genes in the hypertension plus hypercholesterolemia group, compared with sham operated controls.

The IPA network with the ‘largest number of up-regulated focus genes’, contained 22 focus genes with functions in Tissue Development, Connective Tissue Disorders, Developmental Disorder. Focus genes in this network were ALAS2, CCL19, CD2, CD4, CD244, CLEC1B, CNP, DHRS9, FKBP1B, IAPP, KLF13, LTN1, MS4A2, RASGRP4, RNASEL, SLC5A1, SPTA1, SYK, TMEM173, TRAF3IP1, TYMP and UBASH3B. They were related to ERK1/2, Interferon alpha, IL12 complex and SYK (complex), CD2, CD4 and CD244 (Figure 11, Table 5). The next network of up-regulated focus genes had 18 focus genes, with functions in Organ Morphology, Visual System Development and Function, Lipid Metabolism. Focus genes were CA2, CDH17, COL17A1, ELOVL3, FANCC, FCRLA, FLT3, HRH1, MAP3K4, MEP1B, MMP1, NFkBID, NR1D1, PIGR, RET, SERPINB2, SPINK1 and TINF2; they were related to P38 MAPK, NFkB, SERPINB2, MMP1 and TNF (Figure 12, Table 5).

**Figure 13:**
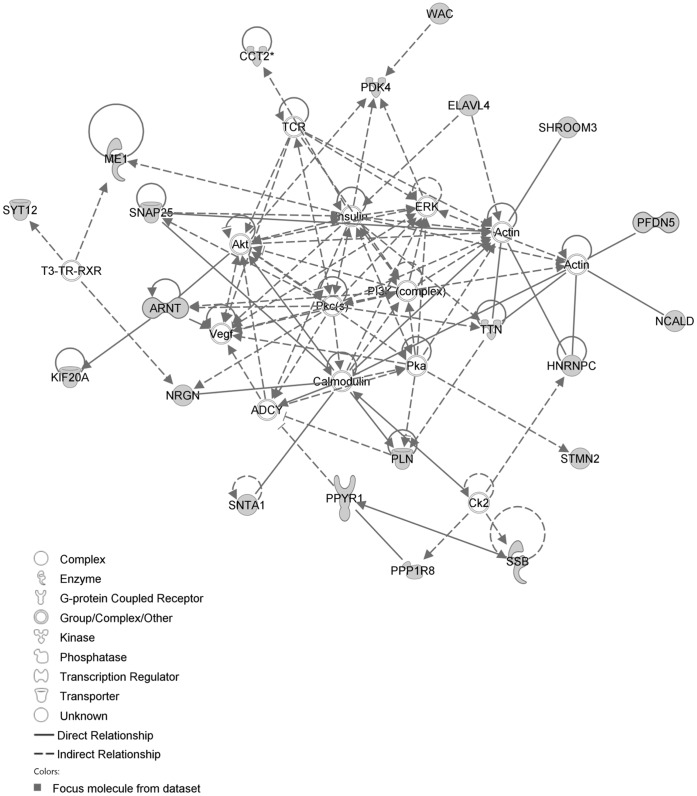
IPA network showing the network with the largest number of down-regulated focus genes in the hypertension plus hypercholesterolemia group, compared with sham operated controls.

**Figure 14:**
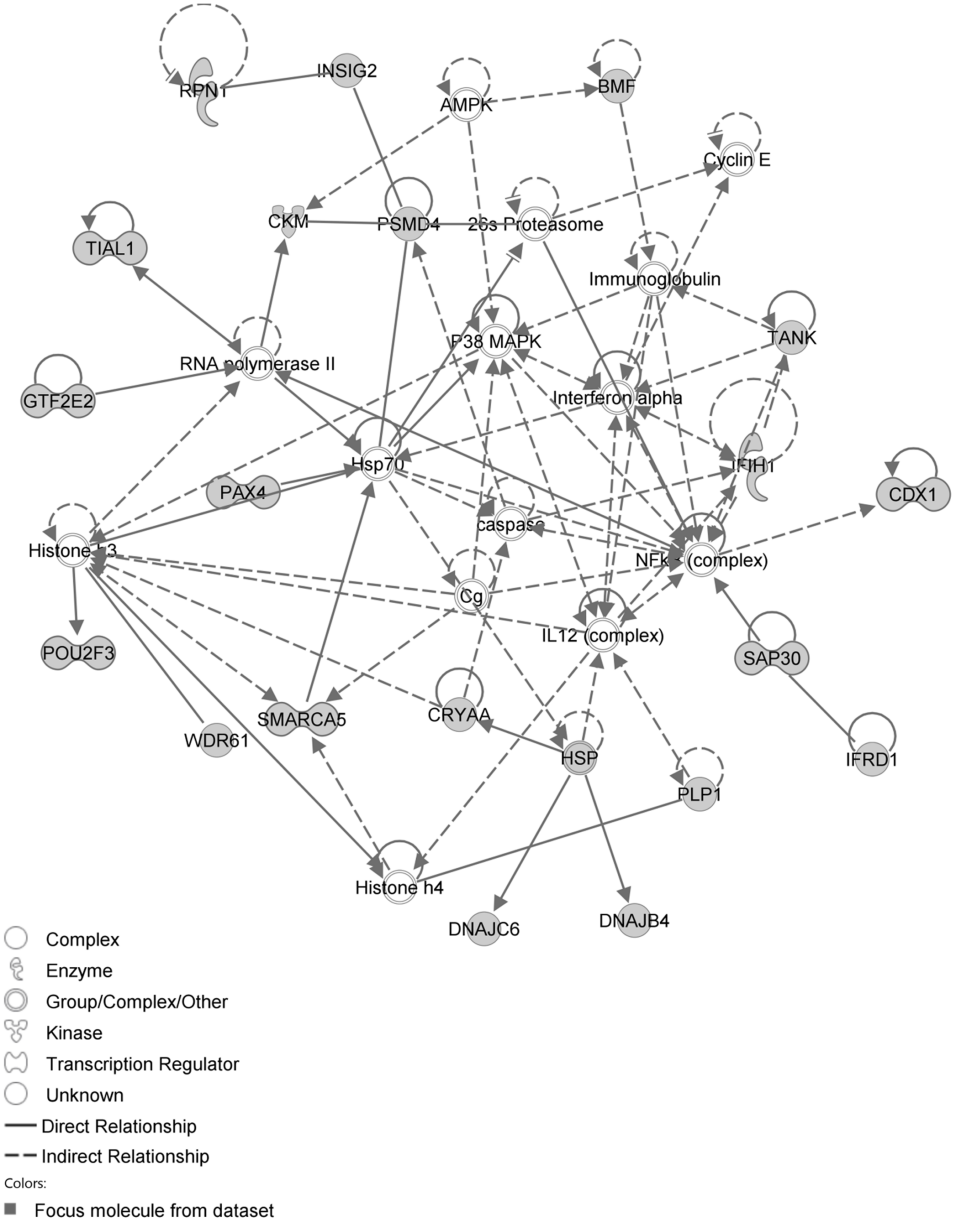
IPA network showing the network with the second largest number of down-regulated focus genes in the hypertension plus hypercholesterolemia group, compared with sham operated controls.

The network with the largest number of down-regulated focus genes contained 21 focus genes with functions in Cardiovascular Disease, Cellular Assembly and Organization, Post-Translational Modification. Focus genes in this network were ARNT, CCT2, ELAVL4, HNRNPC, KIF20A, ME1, NCALD, NRGN, PDK4, PFDN5, PLN, PPP1R8, PPYR1, SHROOM3, SNAP25, SNTA1, SSB, STMN2, SYT12, TTN and WAC. They were related to ERK, Akt, PKC, Vegf, actin and insulin. (Figure 13, Table 6). The next network of down-regulated focus genes had 20 focus genes with functions in Endocrine System Disorders, Gastrointestinal Disease, and Hereditary Disorder. Focus genes were BMF, CDX1, CKM, CRYAA, DNAJB4, DNAJC6, GTF2E2, IFIH1, IFRD1, INSIG2, PAX4, PLP1, POU2F3, PSMD4, RPN1, SAP30, SMARCA5, TANK, TIAL1 and WDR61; they were related to P38 MAPK, NFkB, 26S proteasome, histone H3, interferon alpha and IL12 (Figure 14, Table 6).

#### 2.4. Microarray analyses of the common area between hypertension only- and hypercholesterolemia plus sham groups

The gene expression profile in the common area between the hypertension only- and hypercholesterolemia plus sham groups was also compared with that of sham controls on a normal diet (Fig. 2A). After unknown and repeated genes were omitted, 18 up-regulated and 13 down-regulated genes (greater than 4-fold change) were found (Figure 2). Among the highly up-regulated genes in the MCA of the hypertension only group compared to sham controls were Nibrin like (LOC100352398), TAF15 and ANKAR (Table 7). Among the highly down-regulated genes in the MCA of the hypertension only group were FOXN1, ribosomal protein S3a-like and ADAMTS17 (Table 8).

**Table 7:** Up-regulated genes in the MCA that are common between ‘hypertension only’ and ‘hypercholesterolemia plus sham’ rabbits (both vs. sham controls) with greater than 4-fold change (see Fig. 2).

Gene	Gene Symbol	Corrected *p*-value	Hypertension only	Hyperchole-sterolemia plus sham	Hypertension plus hyperchole-sterolemia
			Fold Change	Fold Change	Fold Change
Nibrin-like	LOC100352398	0.00171	7.92	4.71	3.74
TAF15 RNA polymerase II, TATA box binding protein (TBP)-associated factor, 68 kDa	TAF15	0.00004	7.45	4.71	4.65
ankyrin and armadillo repeat containing	ANKAR	0.00006	7.05	7.99	5.23
N(alpha)-acetyltransferase 25, NatB auxiliary subunit	NAA25	<0.00001	6.43	8.91	18.66
Fanconi anemia, complementation group C	FANCC	<0.00001	6.10	4.25	6.39
Serine proteinase inhibitor, clade B, member 2 (Predicted)	SERPINB2	0.00005	5.63	6.99	9.10
IDI1 protein-like	LOC100346274	0.00042	5.51	4.87	6.67
meprin A, beta	MEP1B	0.00291	5.37	5.12	5.85
Fibroblast growth factor binding protein 1-like	LOC100353835	0.00122	5.01	4.01	4.63
ankyrin repeat and SOCS box-containing 4	ASB4	0.00027	4.98	4.06	3.51
Ankyrin repeat-containing protein C20orf12	DZANK1	<0.00001	4.87	5.04	7.37
signal-induced proliferation-associated 1 like 3	SIPA1L3	0.00001	4.80	8.45	6.50
relaxin/insulin-like family peptide receptor 2	RXFP2	0.00029	4.79	5.62	6.96
guanylate cyclase 2D, membrane (retina-specific)	GUCY2D	0.00008	4.64	4.11	8.34
testis-specific kinase 2	TESK2	0.00571	4.50	5.11	2.41
Ribonuclease, RNase A family, 1 (pancreatic)	RNASE1	<0.00001	4.17	5.62	4.82
DIS3 mitotic control homolog (S. cerevisiae)-like 2	DIS3L2	0.00024	4.17	4.32	3.96
Glucose-fructose oxidoreductase domain containing 2-like	LOC100351150	0.00001	4.01	6.34	12.42

**Table 8:** Down-regulated genes in the MCA that are common between ‘hypertension only’ and ‘hypercholesterolemia plus sham’ rabbits (both vs. sham controls) with greater than 4-fold change.

Gene	Gene Symbol	Corrected *p*-value	Hypertension only	Hyperchole-sterolemia plus sham	Hypertension plus hyperchole-sterolemia
			Fold Change	Fold Change	Fold Change
forkhead box N1	FOXN1	0.00048	−26.20	−56.89	−45.12
Ribosomal protein S3a-like	LOC100354966	<0.00001	−16.54	−5.74	−1.83
ADAM metallopeptidase with thrombospondin type 1 motif, 17	ADAMTS17	0.00001	−15.85	−7.25	−3.06
peptidylprolyl isomerase G (cyclophilin G)	PPIG	<0.00001	−12.61	−6.20	−1.81
hematopoietic prostaglandin D synthase	HPGDS	0.00043	−12.38	−21.44	−13.63
large subunit GTPase 1 homolog (S. cerevisiae)	LSG1	<0.00001	−7.69	−20.38	−1.93
mitochondrial ribosomal protein L15	MRPL15	<0.00001	−6.97	−23.01	−9.57
translocase of outer mitochondrial membrane 5 homolog (yeast)	TOMM5	0.00014	−5.67	−4.05	−1.60
ribosomal protein L26	RPL26	0.00016	−5.27	−14.60	−2.81
family with sequence similarity 177, member A1	FAM177A1	0.00016	−4.41	−10.75	−1.95
Proteasome (prosome, macropain) 26s subunit, non-ATPase, 4	PSMD4	0.00005	−4.34	−17.67	−11.67
Aggrecanase-2	ADAMTS-11	0.00005	−4.26	−9.31	−6.51
BRCA2 and CDKN1A interacting protein	BCCIP	<0.00001	−4.06	−14.00	−2.58

**Figure 15:**
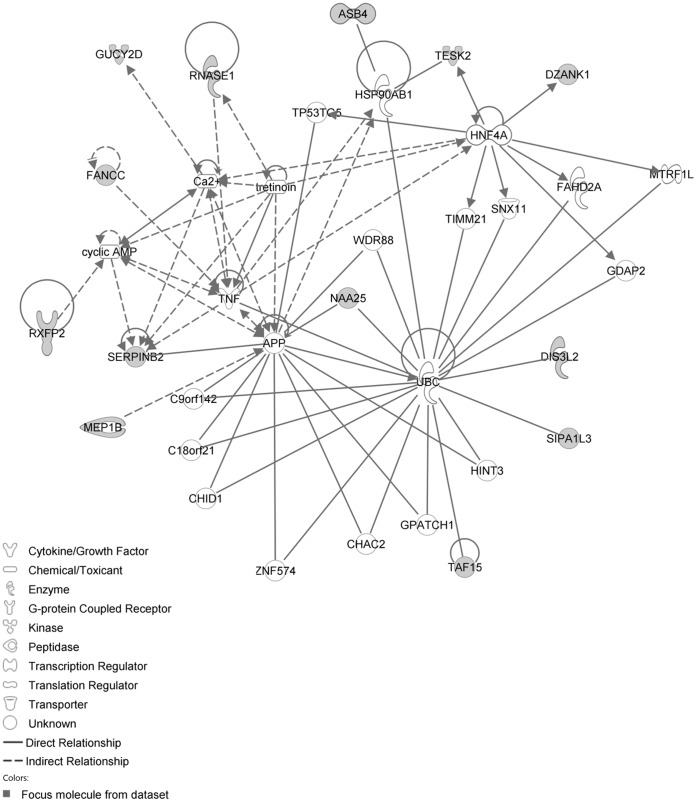
IPA network showing the network with the largest number of up-regulated focus genes in the common area between the hypertension only and hypercholesterolemia plus sham group, compared with sham operated controls.

A single network of up-regulated focus genes was found, which contained 13 focus genes with functions in Drug Metabolism, Endocrine System Development and Function, Lipid Metabolism. Focus genes in this network were ASB4, DIS3L2, DZANK1, FANCC, GUCY2D, MEP1B, NAA25, RNASE1, RXFP2, SERPINB2, SIPA1L3, TAF15 and TESK2. They were related to UBC, APP, SERPINB2, TNF and HNF4A (Figure 15, Table 7).

**Figure 16:**
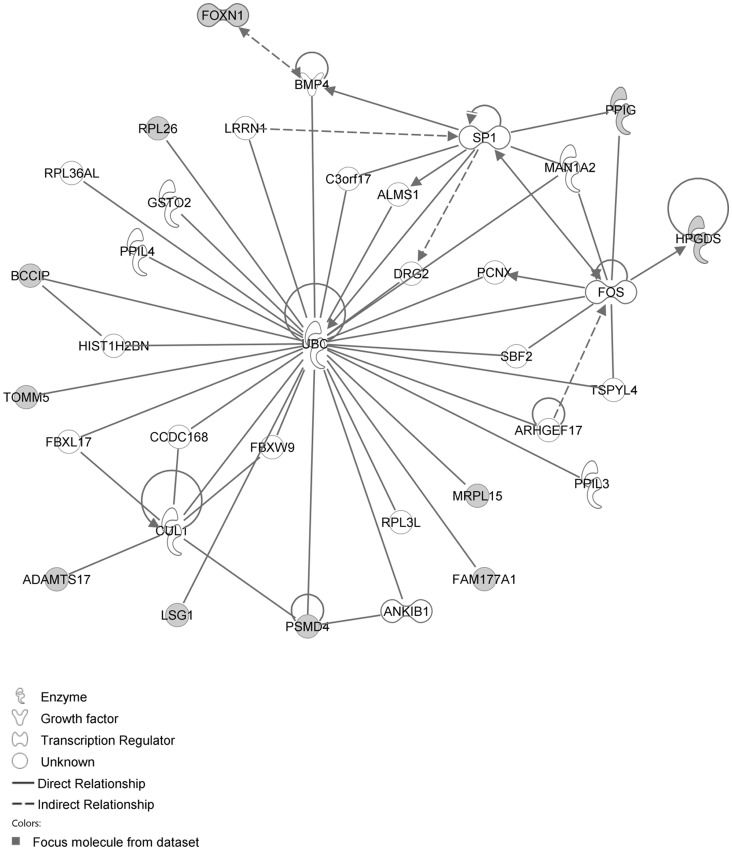
IPA network showing the network with the largest number of down-regulated focus genes in the common area between the hypertension only and hypercholesterolemia plus sham group, compared with sham operated controls.

A single network of down-regulated focus genes was found, which contained 11 focus genes with functions in Cell Morphology, Embryonic Development, and Cellular Compromise. Focus genes were ADAMTS17, BCCIP, FAM177A1, FOXN1, HPGDS, LSG1, MRPL15, PPIG, PSMD4, RPL26 and TOMM5. They were related to UBC (Figure 16, Table 8).

#### 2.5. Microarray analyses of the ‘exclusive area’ in the hypertension plus hypercholesterolemia group

The gene expression profile in the exclusive area of the hypertension plus hypercholesterolemia group was compared with that of sham controls on a normal diet (Fig. 2A). After unknown and repeated genes were omitted, 132 up-regulated and 22 down-regulated genes (greater than 4-fold change) were found (Figure 2). Among the highly up-regulated genes in the MCA of the hypertension plus hypercholesterolemia compared to sham controls were SLFN, MRS2 and HEAT repeat containing (LOC100357872) (Table 9). Among the highly down-regulated genes in the MCA of the hypertension group were SST, ADAM6, and PRLR (Table 10). The panel of genes was analyzed by IPA.

**Table 9:** Up-regulated genes in the MCA that are exclusive to ‘hypertension plus hypercholesterolemia’ rabbits vs. sham controls with greater than 4-fold change.

Gene	GeneSymbol	Corrected *p*-value	Hypertension plus hypercholesterolemia
			Fold change
schlafen family member 14	SLFN14	0.00614	13.64
MRS2 magnesium homeostasis factor homolog (S. cerevisiae)	MRS2	0.00000	8.85
HEAT repeat containing 3	LOC100357872	0.00100	8.34
membrane-spanning 4-domains, subfamily A, member 2	MS4A2	0.00891	8.29
kinesin family member 21A	KIF21A	0.00003	7.31
N(alpha)-acetyltransferase 25, NatB auxiliary subunit	NAA25	0.00099	7.23
General transcription factor IIB	LOC100359058	0.00015	7.00
interleukin 20 receptor, alpha	IL20RA	0.00000	6.96
solute carrier family 18 (vesicular monoamine), member 1	SLC18A1	0.00074	6.64
IQ motif and ubiquitin domain containing	IQUB	0.00104	6.64
family with sequence similarity 71, member C	FAM71C	0.00026	6.63
secretoglobin, family 3A, member 1	SCGB3A1	0.00010	6.42
STAM binding protein-like 1	STAMBPL1	0.00286	6.39
tripartite motif-containing 67	TRIM67	0.00175	6.39
Serum response factor binding protein 1-like	LOC100349313	0.00139	6.34
G protein-coupled receptor 119	GPR119	0.00058	6.30
2′,3′-cyclic nucleotide 3′ phosphodiesterase	CNP	0.00000	6.14
steroidogenic acute regulatory protein	STAR	0.00007	6.05
CD4 molecule (CD4)	CD4	0.00004	6.02
DOMON domain-containing protein FRRS1L	FRRS1L	0.00003	5.99
Tubulin tyrosine ligase-like family, member 5	LOC100359240	0.00019	5.99
thymidine phosphorylase	TYMP	0.00005	5.87
dynein heavy chain domain 1	DNHD1	0.00134	5.87
Insulin-like 5	INSL5	0.00018	5.85
Transcription elongation factor A (SII)-like 2-like	LOC100340955	0.00015	5.75
Solute carrier family 5 (sodium/glucose cotransporter), member 9	SLC5A9	0.00002	5.73
Kruppel-like factor 13	KLF13	0.00007	5.69
Granulate-macrophage stimulating factor (Predicted)	GMCSF	0.00004	5.68
listerin E3 ubiquitin protein ligase 1	LTN1	0.00009	5.66
carboxylesterase 8 (putative)	CES4A	0.00023	5.63
C-type lectin domain family 1, member B	CLEC1B	0.00584	5.60
solute carrier family 15, member 5	SLC15A5	0.00075	5.55
glucosidase, beta, acid 3 (cytosolic)	GBA3	0.00012	5.55
Proline-rich protein 3 (MHC class I region proline-rich protein CAT56)	PRR3	0.00129	5.50
chemokine (C-C motif) ligand 19	CCL19	0.00055	5.50
AchR epsilon subunit	CHRNE	0.00010	5.43
Transmembrane protein C5orf28	C5orf28	0.00319	5.38
dehydrogenase/reductase (SDR family) member 9	DHRS9	0.00168	5.37
FCH and double SH3 domains 1	FCHSD1	0.00015	5.37
Uncharacterized protein C7orf72	C2orf72	0.00114	5.35
family with sequence similarity 71, member F2	FAM71F2	0.00643	5.34
otopetrin 2	OTOP2	0.00006	5.31
elongation of very long chain fatty acids (FEN1/Elo2, SUR4/Elo3, yeast)-like 3	ELOVL3	0.00012	5.30
solute carrier family 7, (cationic amino acid transporter, y+ system) member 13	SLC7A13	0.00134	5.30
Uncharacterized protein C2orf71	C2orf71	0.00177	5.30
solute carrier family 14 (urea transporter), member 1 (Kidd blood group)	SLC14A1	0.00027	5.21
RAS guanyl releasing protein 4	RASGRP4	0.00058	5.11
ATPase, class I, type 8B, member 2	ATP8B2	0.00360	5.07
Cytochrome P450, family 4, subfamily A, polypeptide 5	CYP4A5	0.00061	5.05
Pregnancy-zone protein	PZP	0.00652	5.02
ATPase, H+/K+ exchanging, alpha polypeptide (ATP4A)	ATP4A	0.00000	5.02
CD2 molecule	CD2	0.00064	4.99
3-oxo-5-alpha-steroid 4-dehydrogenase 2-like	LOC100343882	0.00001	4.99
phosphatidylcholine transfer protein	PCTP	0.00001	4.96
transmembrane emp24 protein transport domain containing 6	TMED6	0.00040	4.96
actin-related protein T1	ACTRT1	0.00005	4.94
Wings apart-like homolog	LOC100348678	0.00041	4.94
Fructose-1,6-bisphosphatase 1	FBP1	0.00050	4.88
neuron navigator 2	NAV2	0.00080	4.87
potassium channel tetramerisation domain containing 19	KCTD19	0.00123	4.87
spermatogenesis associated, serine-rich 1	SPATS1	0.00201	4.83
Solute carrier family 5 (sodium/glucose cotransporter), member 1	LOC100009262	0.00001	4.80
tripartite motif-containing 35	TRIM35	0.00084	4.80
cytochrome P450, family 26, subfamily C, polypeptide 1	CYP26C1	0.00224	4.79
Polymeric immunoglobulin receptor Precursor (Poly-Ig receptor)(PIGR)	PIGR	0.00025	4.75
Ras and Rab interactor-like	RINL	0.00135	4.71
ubiquitin associated and SH3 domain containing B	UBASH3B	0.00803	4.70
UDP-N-acetyl-alpha-D-galactosamine:polypeptide N-acetylgalactosaminyltransferase 13 (GalNAc-T13)	GALNT13	0.00000	4.70
PRP18 pre-mRNA processing factor 18 homolog (S. cerevisiae)	PRPF18	0.00144	4.68
spinster homolog 3 (Drosophila)	SPNS3	0.00049	4.68
galactosidase, beta 1-like 3	GLB1L3	0.00003	4.67
iroquois homeobox 2	IRX2	0.00052	4.66
collagen, type XVII, alpha 1	COL17A1	0.00028	4.65
patched 2	PTCH2	0.00005	4.65
DEAQ box RNA-dependent ATPase 1	DQX1	0.00520	4.60
Potassium channel, subfamily K, member 1	KCNK1	0.00217	4.58
purinergic receptor P2Y, G-protein coupled, 10	P2RY10	0.00197	4.57
somatostatin receptor 2	SSTR2	0.00077	4.56
zinc finger, DHHC-type containing 19	ZDHHC19	0.00241	4.55
ret proto-oncogene	RET	0.00036	4.55
Oxytocin receptor	OXTR	0.00517	4.52
serine peptidase inhibitor, Kazal type 1	SPINK1	0.00055	4.50
membrane protein, palmitoylated 2 (MAGUK p55 subfamily member 2)	MPP2	0.00409	4.50
solute carrier family 13 (sodium/sulfate symporters), member 1	SLC13A1	0.00325	4.49
follistatin-like 5	FSTL5	0.00071	4.49
Mitochondrial ribosomal protein S15-like	LOC100345026	0.00064	4.49
TERF1 (TRF1)-interacting nuclear factor 2	TINF2	0.00325	4.49
keratin associated protein 19-4	KRTAP19-4	0.00044	4.48
XK, Kell blood group complex subunit-related, X-linked	XKRX	0.00051	4.47
FK506 binding protein 1B, 12.6 kDa	FKBP1B	0.00274	4.46
phospholipase C, zeta 1	PLCZ1	0.00002	4.45
CD244 molecule, natural killer cell receptor 2B4	CD244	0.00003	4.44
carboxyl ester lipase (bile salt-stimulated lipase) (CEL)	CEL	0.00016	4.44
ADAM metallopeptidase with thrombospondin type 1 motif, 18	ADAMTS18	0.00213	4.43
UDP-glucuronosyltransferase	UGT2C1	0.00035	4.42
fms-related tyrosine kinase 3	FLT3	0.00270	4.41
mitogen-activated protein kinase binding protein 1	MAPKBP1	0.00003	4.41
Ammonium transporter Rh type C (Rhesus blood group family type C glycoprotein)(Rh family type C glycoprotein)(Rh type C glycoprotein)	RHCG	0.00032	4.41
Kelch-like 1 (Drosophila)	KLHL1	0.00003	4.35
leucine rich repeat transmembrane neuronal 4	LRRTM4	0.00002	4.34
proteoglycan 3	PRG3	0.00254	4.33
Ligand of numb-protein X 1-like	LOC100343259	0.00568	4.33
Chymase like protein	LOC100008644	0.00060	4.32
sex comb on midleg-like 4 (Drosophila)	SCML4	0.00854	4.32
BMS1-like, ribosome assembly protein	LOC100339194	0.00017	4.31
Uncharacterized protein C2orf61	C2orf61	0.00893	4.31
Protein FAM55A	LOC100009342	0.00326	4.30
cyclin-dependent kinase 15	CDK15	0.00129	4.28
Rac GTPase activating protein 1 pseudogene	RACGAP1P	0.00023	4.27
family with sequence similarity 170, member A	FAM170A	0.00008	4.23
Mitogen-activated protein kinase kinase kinase 4	MAP3K4	0.00788	4.22
ATPase, H+ transporting, lysosomal 70 kDa, V1 subunit A	ATP6V1A	0.00397	4.19
FRY-like	FRYL	0.00291	4.18
clusterin-like 1 (retinal)	CLUL1	0.00072	4.17
Superiorcervical ganglia, neural specific 10	LOC100344464	0.00228	4.14
histamine receptor H1	HRH1	0.00001	4.13
Cingulin (Predicted)	Cgn	0.00236	4.12
nuclear factor of kappa light polypeptide gene enhancer in B-cells inhibitor, delta	NFKBID	0.00103	4.12
ribonuclease, RNase A family, 1 (pancreatic)	RNASE1	0.00043	4.11
Nuclear prelamin A recognition factor	LOC100344024	0.00002	4.09
actin, beta-like 2	ACTBL2	0.00023	4.09
Ghrelin receptor	LOC100101582	0.00062	4.08
olfactomedin 2	OLFM2	0.00385	4.07
COX10 homolog, cytochrome c oxidase assembly protein, heme A: farnesyltransferase (yeast)	COX10	0.00160	4.07
ribonuclease L (2′,5′-oligoisoadenylate synthetase-dependent)	RNASEL	0.00028	4.06
keratin associated protein 11-1	KRTAP11-1	0.00013	4.04
DnaJ (Hsp40) homolog, subfamily C, member 5 beta	DNAJC5B	0.00016	4.03
receptor (chemosensory) transporter protein 1	RTP1	0.00012	4.03
protocadherin 8	PCDH8	0.00005	4.02
Ubiquitin specific protease 39	LOC100344046	0.00343	4.02
proline/serine-rich coiled-coil 1	PSRC1	0.00571	4.01
cadherin 17, LI cadherin (liver-intestine)	CDH17	0.00459	4.00

**Table 10:** Down-regulated genes in the MCA that are exclusive to ‘hypertension plus hypercholesterolemia’ rabbits vs. sham controls with greater than 4-fold change.

Gene	GeneSymbol	Corrected *p*-value	Hypertension plus hypercholesterolemia
			Fold change
somatostatin	SST	0.00169	−10.49
cellular disintegrin ADAM 6d (ADAM6)	ADAM6	0.00009	−9.13
prolactin receptor	PRLR	0.00001	−8.55
ELAV (embryonic lethal, abnormal vision, Drosophila)−like 4 (Hu antigen D)	ELAVL4	0.00033	−8.49
Proteolipid protein 1	PLP1	0.00578	−8.28
stathmin-like 2	STMN2	0.00129	−7.60
olfactomedin 3	OLFM3	0.00005	−6.99
pyruvate dehydrogenase kinase, isozyme 4	PDK4	0.00711	−6.98
myelin-associated oligodendrocyte basic protein	MOBP	0.00776	−6.94
Paired box protein Pax-4 (Predicted)	PAX4	0.00001	−5.45
SPHK1 interactor, AKAP domain containing	SPHKAP	0.00629	−4.99
neurogranin (protein kinase C substrate, RC3)	NRGN	0.00291	−4.98
GDNF family receptor alpha 4	GFRA4	0.00025	−4.88
Sulfotransferase family 4A, member 1	SULT4A1	0.00285	−4.75
KIAA1549	KIAA1549	0.00019	−4.67
zinc finger CCCH-type containing 7B	ZC3H7B	0.00176	−4.48
inhibin, beta E	INHBE	0.00005	−4.42
POU class 3 homeobox 4	POU3F4	0.00030	−4.33
sema domain, immunoglobulin domain (Ig), transmembrane domain (TM) and short cytoplasmic domain, (semaphorin) 4D	SEMA4D	0.00000	−4.16
membrane-associated ring finger (C3HC4) 6, E3 ubiquitin protein ligase	MARCH6	0.00091	−4.11
ADP-ribosylation factor 4-like	LOC100341862	0.00015	−4.11
potassium channel, subfamily K, member 18	KCNK18	0.00022	−4.05

**Figure 17:**
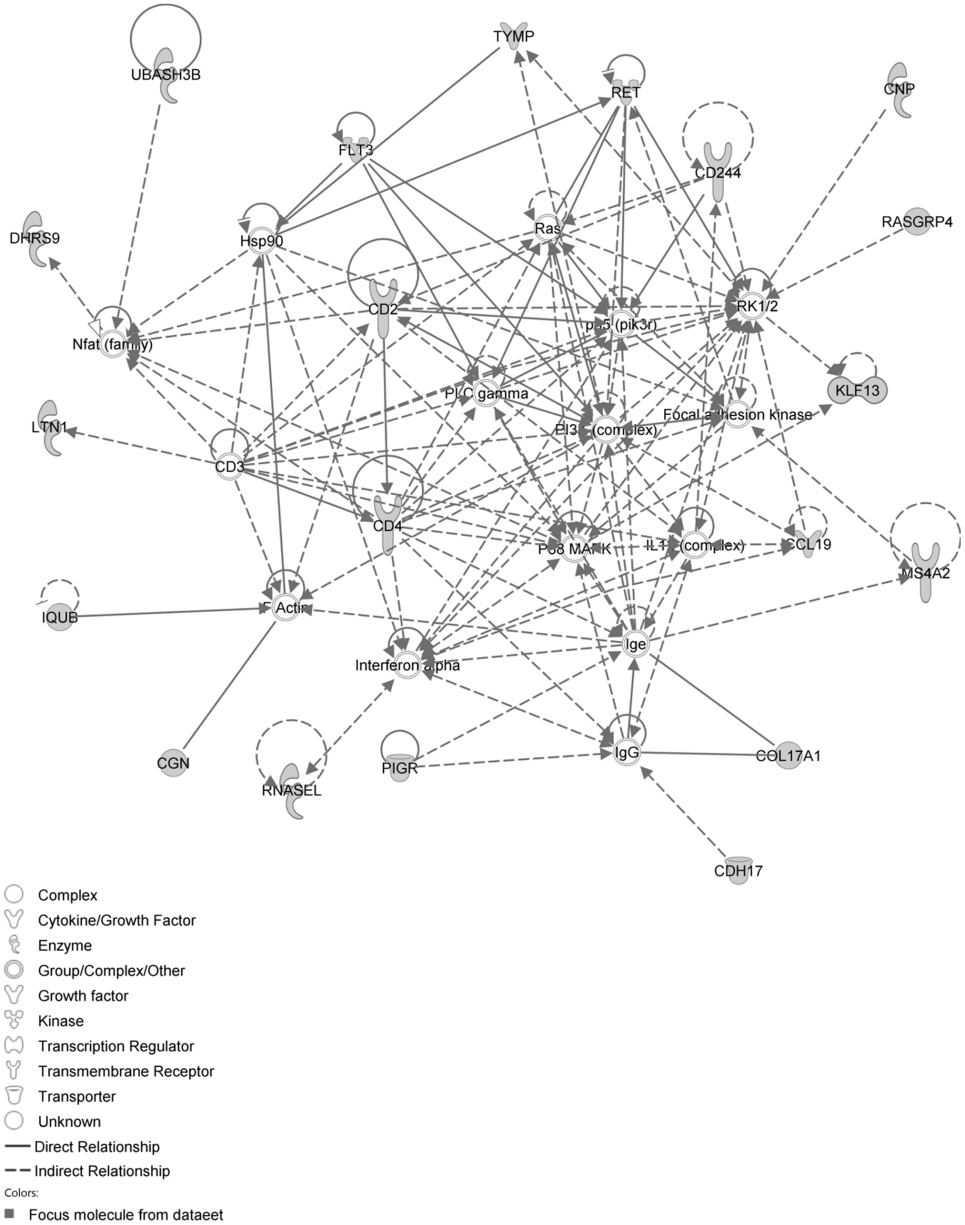
IPA network showing the network with the largest number of up-regulated focus genes in the hypertension plus hypercholesterolemia group (exclusive area), compared with sham operated controls.

**Figure 18:**
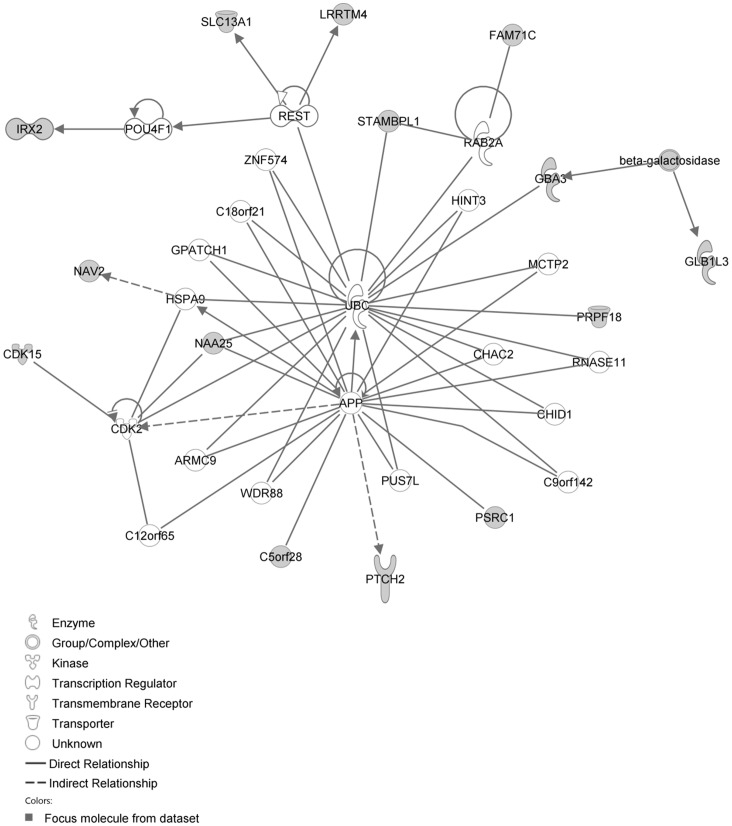
IPA network showing the network with the second largest number of up-regulated focus genes in the hypertension plus hypercholesterolemia group (exclusive area), compared with sham operated controls.

The IPA network with the largest number of up-regulated focus genes contained 20 focus genes with functions in Cell Death and Survival, Cellular Compromise, Inflammatory Response. Focus genes in this network were CCL19, CD2, CD4, CD244, CDH17, CGN, CNP, COL17A1, DHRS9, FLT3, IQUB, KLF13, LTN1, MS4A2, PIGR, RASGRP4, RET, RNASEL, TYMP and UBASH3B. They were related to P38 MAPK, ERK 1/2, interferon alpha and CD2, CD4 and CD244 (Figure 17, Table 9). The next network of up-regulated focus genes had 14 focus genes with functions in Cell Cycle, Nervous System Development and Function, Cell Death and Survival. Focus genes were C5orf28, CDK15, FAM71C, GBA3, GLB1L3, IRX2, LRRTM4, NAA25, NAV2, PRPF18, PSRC1, PTCH2, SLC13A1 and STAMBPL1; they were related to UBC and APP (Figure 18, Table 9).

**Figure 19:**
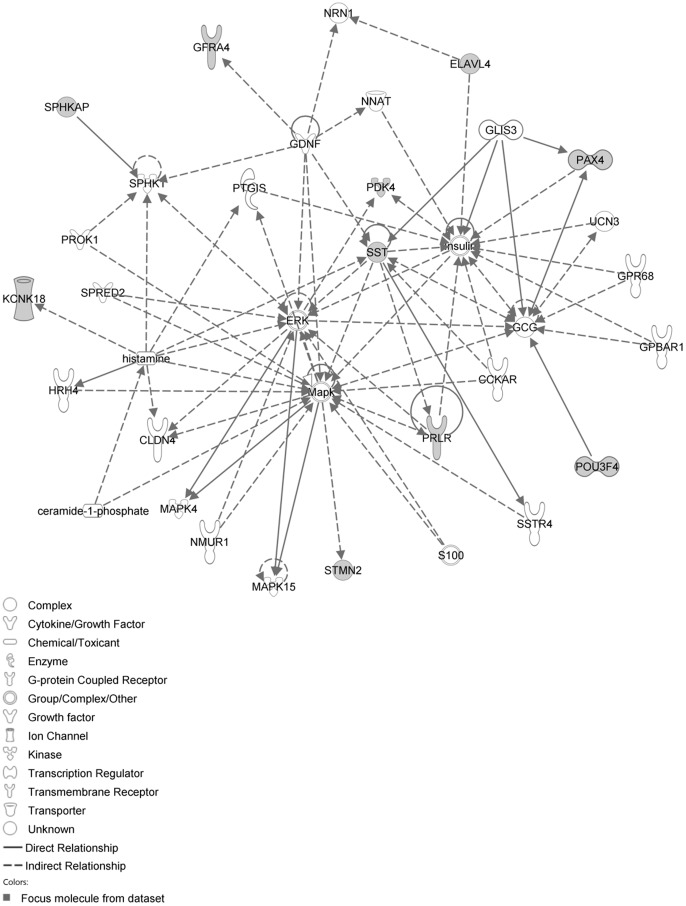
IPA network showing the network with the largest number of down-regulated focus genes in the hypertension plus hypercholesterolemia group (exclusive area), compared with sham operated controls.

**Figure 20:**
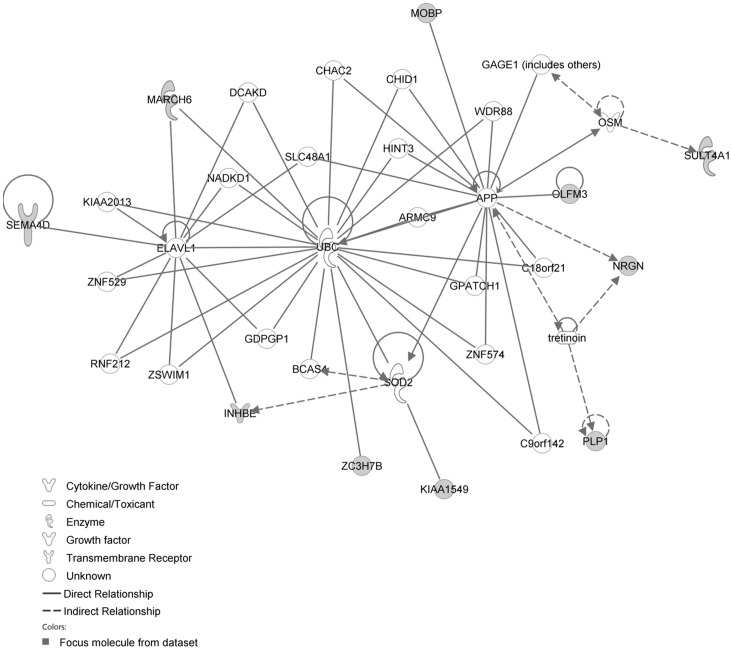
IPA network showing the network with the second largest number of down-regulated focus genes in the hypertension plus hypercholesterolemia group (exclusive area), compared with sham group.

The network with the largest number of down-regulated focus genes contained 10 focus genes with functions in Digestive System Development and Function, Hepatic System Development and Function, Organ Morphology. Focus genes in this network were ELAVL4, GFRA4, KCNK18, PAX4, PDK4, POU3F4, PRLR, SPHKAP, SST and STMN2. They were related to MAPK, ERK and insulin (Figure 19, Table 10). The next network of down-regulated focus genes also had 10 focus genes with functions in Metabolic Disease, Gene Expression, and Cellular Compromise. Focus genes were INHBE, KIAA1549, MARCH6, MOBP, NRGN, OLFM3, PLP1, SEMA4D, SULT4A1 and ZC3H7B; they were related to UBC and APP (Figure 20, Table 10).

### 3. Electron Microscopy of the MCA

**Figure 21:**
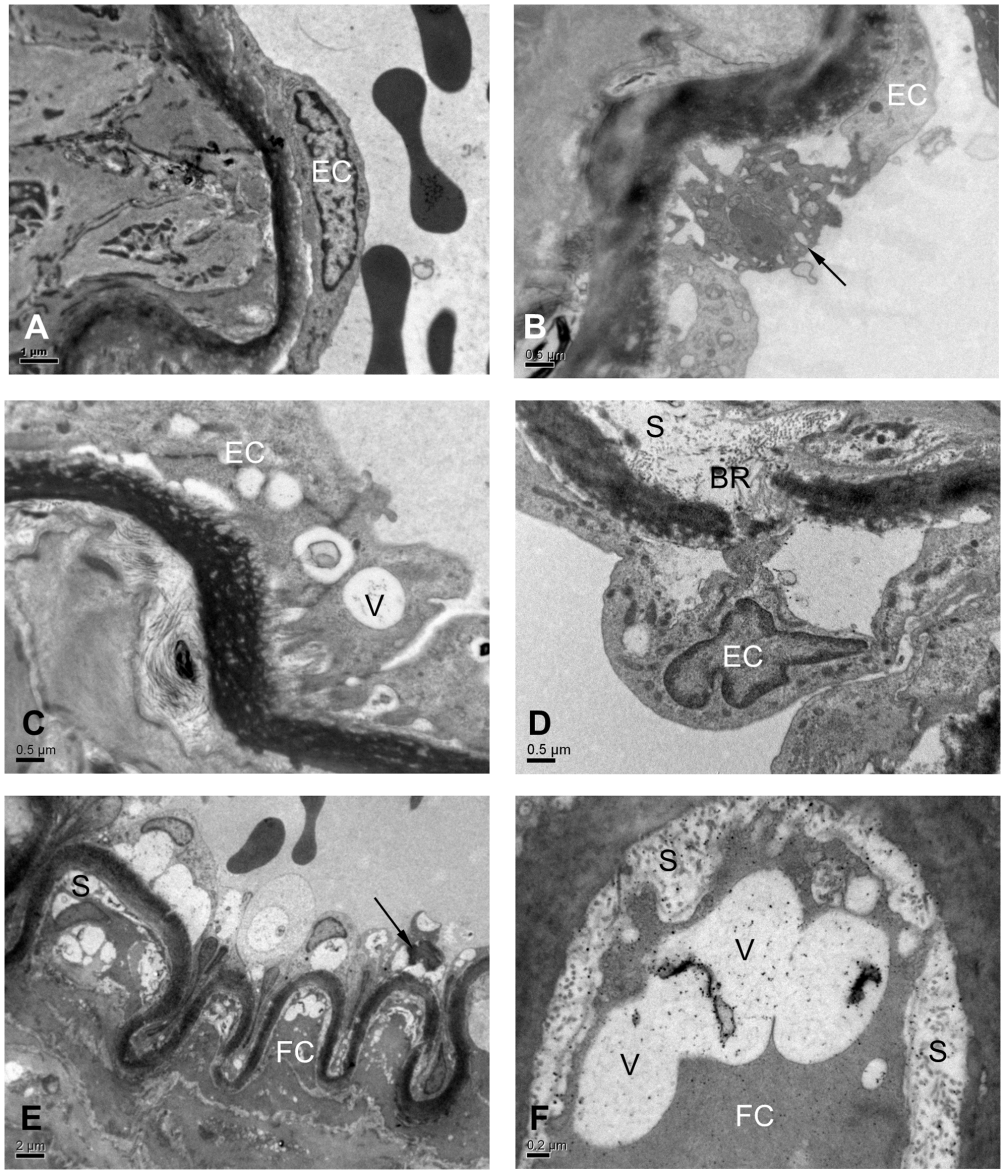
Electron micrographs of the MCA. (A) Sham operated rabbit on a normal diet showing continuous endothelial cells (EC) (B) Hypertension only rabbit, showing a pyknotic cell (arrow) in the endothelial layer (EC). (C) Hypercholesterolemia plus sham rabbit, showing large intracellular vacuoles (V) in endothelial cells. (D) Hypertension plus hypercholesterolemia rabbit, showing breaks in the basement membrane (BR), and a large extracellular space (S) between the basement membrane and the underlying smooth muscle cells. (E) Hypertension plus hypercholesterolemia rabbit, showing a pyknotic cell among the endothelial layer (arrow), and presence subendothelial foam cells (FC). (F) Higher magnification of a foam cell in E, showing intracellular vacuoles, and extracellular spaces (S) containing collagen fibrils. Scale: A = 1 µm, B–D = 0.5 µm, E = 2 µm, F = 0.2 µm.

The MCA of sham operated rabbits on a normal diet showed continuous healthy appearing endothelial cells (Figure 21A). In comparison, the MCA of hypertension only rabbits contained pyknotic endothelial cells (Figure 21B), while that of hypercholesterolemia plus sham rabbits showed large intracellular vacuoles in endothelial cells (Figure 21C). The above changes were exacerbated in the hypertension plus hypercholesterolemia rabbits, and pyknotic endothelial cells, breaks in the basement membrane, and large extracellular spaces were present between the basement membrane and underlying smooth muscle cells (Figure 21D, E). In addition, subendothelial foam cells were observed (Figure 21E, F) consistent with early atherosclerotic changes. The tunica media and tunica adventitia had a normal appearance.

### 4. Vascular Changes in the Aorta

#### 4.1. RT-PCR

**Figure 22:**
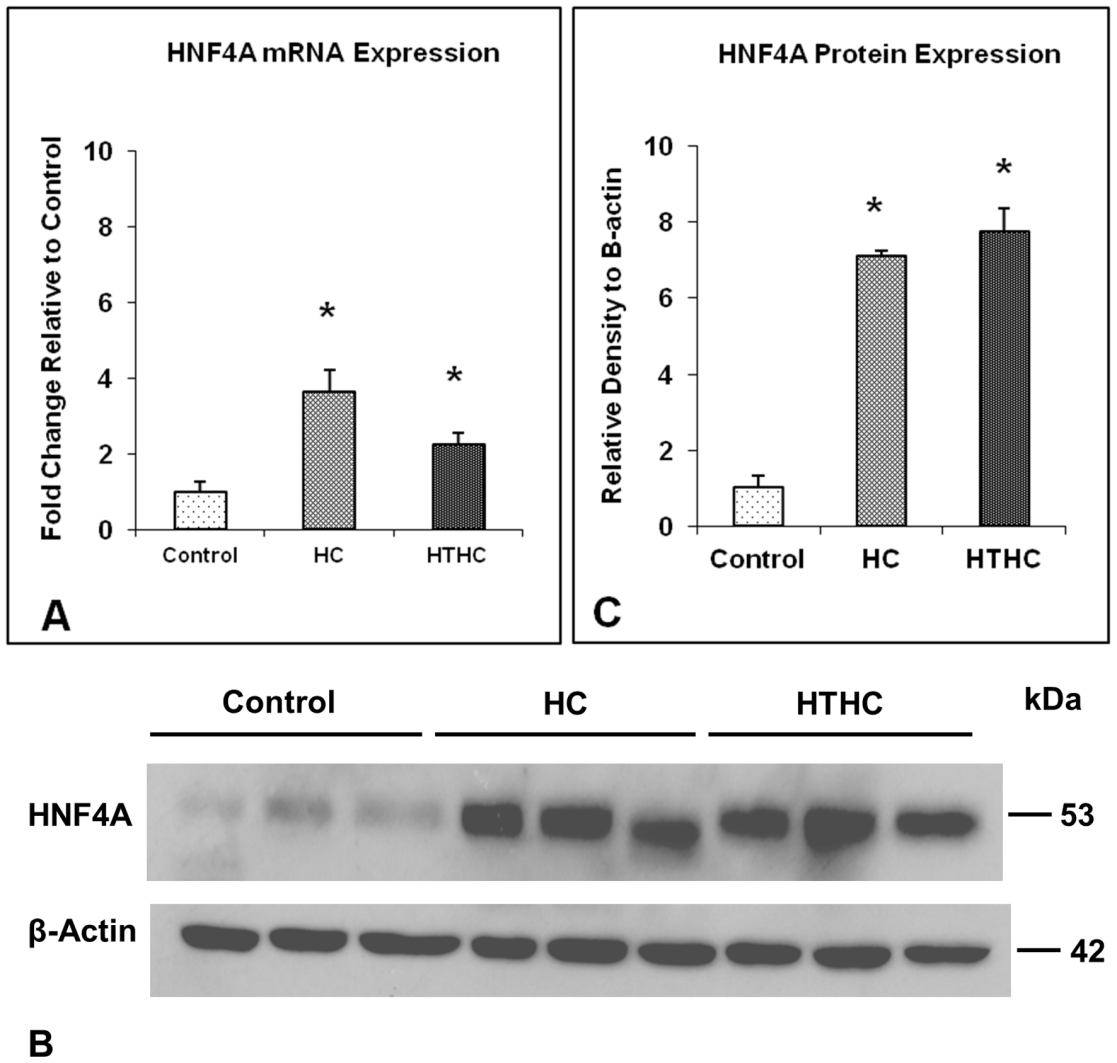
HNF4A expression in the aorta. (A) Real-time RT-PCR analyses of HNF4A in the aorta of control, hypercholesterolemia plus sham, and hypertension plus hypercholesterolemia rabbits. The mean and standard error are shown. *p<0.05 vs. controls by one-way ANOVA with Bonferroni’s multiple comparison post-hoc test (n = 4 in each group). (B) Western blot analyses of HNF4A in the aorta of control, hypercholesterolemia plus sham and hypertension plus hypercholesterolemia rabbits. (C) Densitometric analyses of HNF4A protein, normalized to ß-actin. The mean and standard error are shown. *p<0.05 vs. control by one-way ANOVA with Bonferroni’s multiple comparison post-hoc test (n = 3 in each group). Abbreviations as in Fig. 1.

HNF4A mRNA expression was increased in the aorta of the hypercholesterolemia plus sham-operated group- and hypertension plus hypercholesterolemia group (3.64 and 2.25-fold change respectively), compared to controls on a normal diet (Figure 22A).

#### 4.2. Western blot analyses

The antibody to HNF4A detected a 53 kDa band in homogenates of the aorta consistent with the expected molecular weight of the protein (Figure 22B). Increased density of the HNF4A band relative to beta actin was found in homogenates from hypercholesterolemia plus sham- and hypertension plus hypercholesterolemia group compared to controls, indicating up-regulation of HNF4A protein expression after exposure to hypertension and/or hypercholesterolemia (Figure 22C).

#### 4.3. Histochemistry and immunohistochemistry

**Figure 23:**
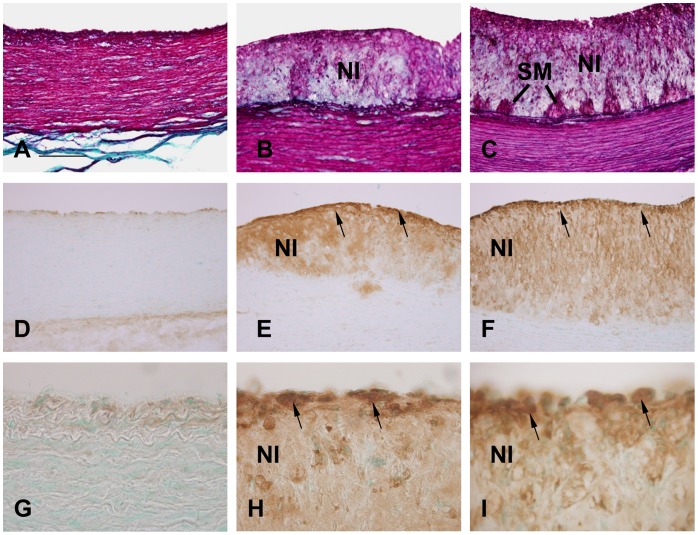
Histochemical and immunohistochemical staining of the aorta from rabbits exposed to stroke risk factors. A, D, G: Rabbits on normal diet. B, E, H: Hypercholesterolemia plus sham group. C, F, I: Hypertension plus hypercholesterolemia group. A-C: Aorta of rabbits stained by Masson’s Trichrome. Increased thickness of the neointima (NI) is seen in the hypercholesterolemia plus sham group (B, arrow). The changes are exacerbated in the hypertension plus hypercholesterolemia group (C, arrow). SM = smooth muscle cells in the neointima. D, E, F: Aorta of rabbits immunostained with a mouse monoclonal antibody to HNF-4A. Very little or no labeling is present in normal rabbits, but dense staining is observed in endothelial cells in the hypercholesterolemia plus sham, and hypertension plus hypercholesterolemia rabbits (arrows). G, H,I: Higher magnification of the aorta of rabbits immunostained with mouse monoclonal antibody to HNF-4A, showing dense staining in endothelial cells of hypercholesterolemia plus sham, and hypertension plus hypercholesterolemia rabbits (arrows). Scale: A-F = 200 µm, G-I = 70 µm.

The general structure of the aorta was examined by Masson’s Trichrome staining (Figure 23A–C). Hypercholesterolemia plus sham rabbits as well as the hypertension plus hypercholesterolemia rabbits showed neointimal formation along part of the circumference of the vessel. This was associated with migration of red-staining, smooth muscle cells from the tunica media into the neointima (Figure 23C). The changes were more pronounced in the hypertension plus hypercholesterolemia than the hypercholesterolemia plus sham rabbits (Figure 23A–C). The tunica media and tunica adventitia had a normal appearance.

Immunostaining of the aorta with HNF4A antibody showed that the endothelial layer of hypercholesterolemia plus sham group and the hypertension plus hypercholesterolemia group were densely stained for HNF4A, compared to controls (Figure 23D–I). Immunolabel was observed in the nucleus and cytoplasm of endothelial cells and other cells near the endothelial layer. No staining was observed in the tunica media or adventitia (Figure 23D-I). These results indicate that increased HNF4A gene expression in the aorta occurred mainly in endothelial cells.

## Discussion

The present study was carried out to elucidate differential gene expression changes in the MCA of rabbits exposed to two stroke risk factors, i.e. hypertension and/or hypercholesterolemia. Of the DEGs in the MCA that were altered by a single risk factor, hypertension alone vs. sham controls on a normal diet, FAM167A had the highest fold change, followed by CERS3 and FAM53C. FAM167A encodes a ubiquitously expressed gene, the function of which remains unknown. CERS3 (ceramide synthase 3) catalyzes the condensation of sphinganine and fatty acyl-coenzyme A to form dihydroceramide, which is oxidized to ceramide [30]. Among the down-regulated DEGs in the MCA of the hypertension only group were FOXN1, NSRP1 and THUMPD3. Forkhead transcription factor is essential for thymus development [31] and keratinocyte differentiation [32].

Of the DEGs in the MCA that were up-regulated in the hypercholesterolemia plus sham group vs. sham controls on a normal diet, SLFN14 had the largest fold change, followed by CA1 and Gap protein alpha-3 protein-like (LOC100357902). SLFN14 is part of the Schlafen family of proteins which have growth regulatory properties [33]. CA1 (carbonic anhydrase I) is a member of the carbonic anhydrase family that catalyzes the hydration and dehydration of CO_2_/H_2_CO_3_ [34] and gene mutation is associated with rheumatoid arthritis [35].

Among the DEGs that were down-regulated in the hypercholesterolemia plus sham group vs. sham controls were beta tropomyosin (LOC100125984), PFDN5 and CUL3. The latter is a member of the cullin protein family [36], [37] involved in ubiquitination [38], and gene polymorphism is associated with hypertension [39].

Of the DEGs in the MCA that were up-regulated in the hypertension plus hypercholesterolemia group vs. sham controls on a normal diet, EPHA1 had the largest fold change, followed by SP110, and SLFN14. EPHA1 (Ephrin receptor A1) has recently been identified in large-scale genome-wide association studies to be one of the risk genes for late onset Alzheimer’s disease (AD) [40], [41]. SLFN14 has been mentioned in the hypertension only group.

Among the DEGs that were down-regulated in the hypertension plus hypercholesterolemia group were FOXN1, TNFRSF11B, and GAPDHS. TNFRSF11B (tumor necrosis factor receptor superfamily, member 11b) is the gene encoding osteoprotegerin, a member of the tumor necrosis factor receptor superfamily of cytokines [42] involved in bone resorption [43] and vascular diseases [44], [45]. Gene polymorphism of TNFRSF11B is a risk factor for ischemic stroke [46]. FOXN1 has been mentioned in the hypertension only group.

Of the DEGs in the that were up-regulated in common between the hypertension only- and hypercholesterolemia plus sham groups (both vs. sham controls), Nibrin like (LOC100352398) showed the largest fold change, followed by TAF15, and ANKAR. TAF15 (TATA box binding protein associated factor 15) is a member of the FET family of RNA-binding proteins [47]. ANKAR (ankyrin and armadillo repeat containing) is one of the genes affected in aortic dilatation/dissection [48].

Among the DEGs that were down-regulated in common, between the hypertension only, and hypercholesterolemia plus sham groups (both vs. sham controls) were FOXN1, Ribosomal protein S3-like (LOC100354966) and ADAMTS17 (ADAM metallopeptidase with thrombospondin type 1 motif, 17). The ADAMTS family of genes is involved in cancer, arthritis and coagulation [49], and variants of ADAMTS17 are associated with pediatric stroke [50].

The network in the MCA with largest number of up-regulated focus genes affected by hypertension showed many focus genes related to the ‘node molecule’, ubiquitin, a regulatory protein that directs other proteins to the proteasome [51]. Apart from chronic neurodegenerative diseases, the ubiquitin-proteasome system is implicated in brain ischemia by inducing cell damage or leukocyte infiltration into the brain [51]. The network with the second largest number of up-regulated molecules was related to P38 MAPK and ERK. P38 MAPK (mitogen-activated protein kinase) is a member of the MAPK family involved in stress-related signal transductions [52], and sustained activation can result in apoptosis in various cell types [53], [54], [55]. Inhibition of P38 activity is reported to reduce infarct volume and neurological deficits [52], [56] as well as cytokine expression after stroke [52]. ERK1/2 (extracellular signal-regulated kinase 1/2) is a well-characterized member of the MAPK family that is activated by mitogens or stressors, and plays an important role in cell differentiation and proliferation [57], [58]. Phosphorylated ERK1/2 is increased after cerebral ischemia/reperfusion, and the ERK pathway is involved in both neuroprotection and cell death [58]. Other focus genes in this network were related to NF-κB, SERPINB2, MMP1 and APP. NF-κB (nuclear factor-kappa B) is a central regulator of inflammation and apoptosis [59] and is active in many chronic inflammatory diseases including atherosclerosis [60]. It could have damaging effects in cerebral ischemia [61], [62], and inhibition of NF-κB decreases neointimal formation [63], [64], [65] and reduces infarct volume and neurological deficits after stroke [66]. On the other hand, NF-κB activation could also be neuroprotective [67], [68], as it participates in cell death/survival pathways through regulation of pro- and anti-apoptotic genes [69], [70]. SERPINB2 (serpin peptidase inhibitor, clade B (ovalbumin), member 2), also known as plasminogen activator inhibitor (PAI) type 2 is a physiological inhibitor of urokinase plasminogen activator (uPA) [71]. Increased SERPINB2 expression is found in the AD brain [72], and after brain ischemia or trauma, particularly in the basement membrane and endothelial cells of vessels adjacent to the lesion [73]. MMP1 (matrix metallopeptidase 1) belongs to a family of protein-digesting enzymes that degrades the extracellular matrix in both physiological and pathological conditions including stroke [74]. MMP1 is increased in atherosclerotic plaques [75] and gene polymorphism is suggested to influence the risk of coronary heart disease [76]. APP (β-amyloid precursor protein) can be processed by an amyloidogenic pathway to form A-beta. The latter and vascular risk factors [77], [78] play important roles in the pathogenesis of AD [79], [80], and endothelial dysfunction in APP overexpressing mice increases the susceptibility of the brain to AD pathology [81] and cerebral ischemia [82].

The network in the MCA with the largest number of down-regulated focus genes affected by hypertension was related to MAPK, ERK 1/2, Akt, 26s proteasome, histone and PKC, while the network with the second largest number of down-regulated focus genes was related to UBC. Akt is a serine/threonine kinase that is activated by PI3K in various growth factors-mediated signaling cascades [83]. The PI3K/Akt signaling pathway is important in mediating cell survival [83], [84] and Akt activity is shown to confer neuroprotection after ischemic brain injury [85], [86]. PKC (protein kinase C) is a serine-threonine kinase family that is important in regulating cellular functions, and several isozymes of PKC such as PKC<$>\raster="rg1"<$>, PKCδ and PKCγ, are associated with cerebral ischemic and reperfusion injury [87], [88]. The use of PKCδ peptide inhibitors is reported to alleviate reperfusion injury and reduce stroke infarct size [87], [88], [89]. 26s Proteasome is an essential component of the ubiquitin-proteasome system which functions to degrade cellular proteins [90]. The exact role of the ubiquitin-proteasome system in cerebral ischemia is at present unclear, and deleterious effects of proteasome malfunction, as well as beneficial effects of proteasome inhibition on cerebral ischemia have been reported [51], [90]. Histones affect gene transcription by binding to DNA; hence changes in histone H3 may affect the expression of downstream molecules in vessels during hypercholesterolemia. Interestingly, promising outcomes from the use of histone deacetylase (HDACs) inhibitors have been reported in preclinical stroke models [91].

Gene network analysis of the MCA after hypercholesterolemia (plus sham operation), surprisingly, showed very similar networks as that the hypertension only group. This was despite a relatively low percentage of genes in the common area between these two conditions (20.8% of hypertension genes and 6.8% of hypercholesterolemia genes were in the common area, respectively). The results suggest recruitment of different focus genes that are related to similar ‘node molecules’, as in the following example: the network in the MCA with largest number of up-regulated focus genes affected by hypercholesterolemia showed many molecules related to APP. This is similar to hypertension only rabbits, and could indicate synergism of the two risk factors in affecting the expression of molecules related to APP. Other focus genes were related to tretinoin or all-trans retinoic acid (ATRA) [92], a molecule known to modulate A-beta associated memory deficits and neuropathological alterations in animal models of AD [92], [93].

The network with the second largest number of up-regulated focus genes also had many similarities with the hypertension only group, with P38 MAPK, ERK 1/2, NFkB, SERPINB2 and Akt being central players, together with focus genes related to interferon alpha and VEGF. Interferon alpha is a member of a family of nonspecific antiviral agents with immunomodulatory and cytostatic properties [94]. Although other isoforms of the interferon family such as interferon beta and gamma are associated with atherosclerosis [94], a possible role of interferon alpha in this condition is yet unknown. Vegf (vascular endothelial growth factor) is involved in conditions such as atherosclerosis, cerebral edema, brain and vascular repair following ischemia [95], and plasma vegf values are increased immediately after stroke [96].

The network in the MCA with largest number of down-regulated focus genes affected by hypercholesterolemia is related to ubiquitin, 26s proteasome and Akt, and the network with the second largest number of down-regulated focus genes is related to histone H3 and F actin. These changes in ubiquitin, proteasome, and histone H3 are very similar to that of hypertension only animals. Down-regulation of genes related to actin may affect process outgrowth or motility of vascular cells, and actin cytoskeleton signaling is one of the functional pathways that are related to male-specific ischemic stroke genes [97].

The network in the MCA with largest number of up-regulated focus genes affected by hypertension plus hypercholesterolemia showed many molecules related to ERK 1/2, interferon alpha, IL12, SYK, CD2, CD4, and CD244. IL12 (interleukin 12) is a proinflammatory and immunomodulatory cytokine [98] released in response to tissue injury [99]. Elevated serum levels of IL12 are observed in patients with acute myocardial infarction [100], traumatic brain injury [101] and ischemic stroke [102]; in addition, IL 12 signaling is related to female-specific ischemic stroke genes [97]. SYK (spleen tyrosine kinase) is a non-receptor tyrosine kinase [103] involved in signaling cascades in platelets [104]. The use of inhibitors of SYK is a potential treatment for occlusive vascular disease, due to its effect in modulating platelet aggregation and thrombus formation [104]. SYK also phosphorylates tau [105] and α-synuclein [106]. CD2 (CD2 molecule) is a member of the immunoglobulin superfamily and mediates the activation of T and natural killer cells [107]. CD4 (CD4 molecule) is expressed on the surface of T cells [108] and CD4^+^ T cells are regulators of humoral and cellular immune response [109]. Lack of CD4^+^ T cells is associated with decreased lesion size after stroke [110]. CD244 (CD244 molecule, natural killer cell receptor 2B4) is part of the ‘signaling lymphocyte activation molecule’ (SLAM) family of receptors [111] and is present on natural killer cells, activated CD8^+^ T cells, and monocytes [112], [113]. It is postulated that CD244 may be involved in a pathway that promotes inflammatory neurological disease [114]. The network with the second largest number of up-regulated focus genes was related to P38 MAPK, NFkB, SERPINB2, MMP1 and Tnf (family). TNF (tumor necrosis factor) plays a key role in increasing the expression of inflammation related genes in atherosclerosis [115], and expression is increased in brain during ischemia [116] or in patients who suffer intracerebral hemorrhage [117]. Antagonism of the TNF-α receptor modulates neurovascular injury and improves neurobehavioral outcomes, after intracerebral hemorrhage in mice [118].

The network in the MCA with largest number of down-regulated focus genes affected by hypertension plus hypercholesterolemia showed many molecules related to ERK, Akt, PKC, Vegf, actin, and insulin. Diabetes mellitus and insulin resistance increase the risk of ICLAD and stroke [119], [120], [121], [122], [123]. Moreover, patients with diabetes show poorer post-stroke functional outcomes in terms of mortality [124], [125] and cognition [126]. These findings may be related to the effect of insulin on activation of NFkB and generation of pro-inflammatory factors involved in atherogenesis [127]. The network with the second largest number of down-regulated focus genes was related to P38 MAPK, NFkB, 26s proteasome, histone H3, interferon alpha and IL12.

The common area between the hypertension only- and hypercholesterolemia plus sham groups showed up-regulated focus genes related to UBC, APP, SERPINB2, TNF and HNF4A, and down-regulated focus genes related to UBC. HNF4A (hepatocyte nuclear factor 4 alpha) is a ligand activated nuclear transcription factor that regulates the expression of many genes involved in lipid transport and glucose metabolism and are associated with cell cycle, immunity, apoptosis, stress response and cancer [128]. Increased expression of HNF4A was shown by RT-PCR and Western blot, and the protein immunolocalized to endothelial cells in the aorta. Since HNF4A suppresses hepatocyte proliferation in adult mice [129], increased expression may likewise affect the turnover of endothelial cells. The effect of this on atherosclerosis is unclear, although excess proliferation of vascular smooth muscle cells is known to have an atherogenic effect [130].

The hypertension plus hypercholesterolemia ‘exclusive’ area showed up-regulated and down-regulated pathways, related to many of the node molecules mentioned above. This area is tentatively interpreted as containing genes that are exacerbated by two risk factors, and the effects tends towards recruitment of additional molecules into existing networks rather than initiation of new networks.

An ischemic cerebrovascular event or transient ischemic attack is a risk factor for a subsequent event [21], [22], [131]. The reasons for this are multifactorial [132] and may be partly due to presence of existing atherosclerotic lesions. The present findings extend these concepts to the gene network level, and delineates pathways related to NF-κB and TNF that are involved in inflammation and atherosclerosis [60], [133], as well as focus genes related to ubiquitin, proteasome, histone, HNF4A, insulin and APP. It is hoped that these could provide a framework for better understanding of pathophysiological mechanisms, and development of new therapies for ICLAD.
